# Factors influencing unrelated stem cell donation a mixed‐methods integrated systematic review

**DOI:** 10.1111/bjhp.12758

**Published:** 2024-10-24

**Authors:** Jessica Forbes, Paul Rice, Jenny Groarke, Emma Berry, Henrietta Graham, Lisa Graham‐Wisener

**Affiliations:** ^1^ Centre for Improving Health‐Related Quality of Life, School of Psychology Queen's University Belfast Belfast Northern Ireland; ^2^ School of Psychology National University of Ireland Galway Galway Ireland; ^3^ The Centre of Lifestyle Medicine and Behaviour, School of Sport Exercise and Health Sciences Loughborough University Loughborough UK

**Keywords:** haematopoietic, behaviour, donation, stem‐cell, unrelated

## Abstract

**Purpose:**

There is an imbalance between demand for and availability of stem cell donors worldwide. The purpose of this systematic review is to provide the first comprehensive understanding of facilitators and barriers influencing unrelated stem cell donation (USCD) in adults, through a data synthesis of qualitative and quantitative evidence. Identification of the facilitators and barriers associated with stem cell donation intention and behaviour is essential to inform the development of behaviour change interventions to meet the current demand.

**Methods:**

Four databases were searched (Embase, PsycINFO, MEDLINE and CINAHL) and the last search was in February 2021. The search was limited to studies written in English and published from 1980 to present. Screening, quality assessment, data extraction and data synthesis incorporating the COM‐B model were undertaken in line with the Joanna Briggs Institute methodology for an integrated mixed‐methods review.

**Results:**

Fifty studies were included in the review, analysis and mapping produced four integrated findings. Donation‐related knowledge was a facilitator and conversely, lack of knowledge was a barrier to donation related behaviours. Perceived convenience, positive social influences, religious beliefs and the accessibility of positive donation‐related social norms promoted positive donation related behaviours. Altruism and sense of duty were the most commonly cited motives for donation related behaviours.Through mapping to the COM‐B model, Communication/Marketing, and Service Provision are the primary policy categories that can be used to change donation related behaviours.

**Conclusion:**

Future interventions should focus on targeted education regarding unrelated stem cell donation and creating recruitment campaigns that emphasise the life‐saving potential of donation.

**Statement of contribution**

**What is already known on this subject?**

- There is an imbalance between demand for and availability of stem cell donors worldwide.
- Haematopoietic stem‐cell transplantation is a potentially curative treatment for selected individuals.
- The majority of haematopoietic stem cell transplants are from unrelated donors, but the current demand is not being met.

**What does this study add?**

- Donation‐related knowledge, social influence, and altruism influence intention to donate.
- Higher levels of ambivalence increase attrition from bone marrow registries.
- Lack of information and lack of contact from registry staff increase attrition from registries.
- Theory and evidence based recommendations for future interventions that target behaviour change in potential future donors by focusing on communication and service provision.

## INTRODUCTION

Allogenic haematopoietic stem‐cell transplantation (HSCT) is a potentially lifesaving therapy for several blood cancers, such as leukaemia and myeloma, as well as a range of non‐malignant conditions (Billen et al., 2017; Garcia et al., 2013; Gragert et al., 2014). HSCT can involve donation of peripheral blood stem cells or bone marrow, from genetically related or unrelated donors. HSCT from unrelated donors has been shown to be successful for individuals who lack a suitable family donor (Chen et al., 2013; Gragert et al., 2014; Passweg et al., 2017). Requests for HSCT donations doubled in the decade prior to 2016 because of advancements in treatment (Balassa et al., 2019; Lown & Shaw, 2013), yet availability of unrelated stem cell donation (USCD) is not meeting demand (Switzer et al., 2013).

Donors must be matched according to multiple factors including human leukocyte antigen (HLA) tissue type to increase the chances of successful treatment (Kollman et al., 2016). This means stem cell registries require access to large numbers of reliable unrelated donors. Recruitment to and maintenance of a donor registry can be expensive, for example, the annual cost of maintaining the United States (US) National Marrow Donation Program (NMDP) registry of 4 million unrelated donors for HSCT is $30 million (Kollman et al., 2001, 2016). Therefore, registries must be efficient in targeting and recruiting unrelated donors.

Donor registries face several challenges, including the ageing of current registrants, a lack of donors from ethnic minority groups, a lack of male donors, and a high attrition rate, particularly among donors from ethnic minority groups (Balassa et al., 2019; Fingrut et al., 2018). Increased age has been associated with medical unsuitability for donation and is an established factor for chronic graft versus host disease, a possible complication of donation (Balassa et al., 2019; Shouval et al., 2019). Depending on local regulations the upper age limit is normally between 55 and 60, with the most common reason for attrition from register being age (Foeken, et al., 2010). Attrition of unrelated donors has a detrimental impact on both the recipient's health outcome and the registry's financial status (Lown & Shaw, 2013; Switzer et al., 2004). Therefore, registries need to focus on composition, in addition to size. A common challenge faced by registries worldwide is low recruitment of individuals from ethnic minority groups (Fingrut et al., 2017; Lown & Shaw, 2013; Switzer et al., 2013). Consequently, ethnic minority recipients are less likely to find a suitable donor match. Male donors are underrepresented on worldwide registries but are more commonly selected by transplant centres due to less post‐operative complications and poor outcomes following female‐donor male recipient sex mismatches (Fingrut et al., 2018). Conversely, male donors (aged 18–32 years) report multiple benefits with fewer post‐donation complications compared to females and young donors report increased overall time on the register (Fingrut et al., 2017). Registries worldwide are strategically targeting younger populations, males, and ethnic minority groups in their donation recruitment drives. To improve the size and composition of worldwide USCD registries, there is a need to understand the facilitators, barriers and demographics influencing donation behaviour across the USCD pathway which encompasses intention to register, intention to donate and donation. It is necessary to investigate facilitators and barriers that influence donation behaviour in both the general population and the key groups identified; younger adults, ethnic minorities and males.

Systematic reviews have provided a comprehensive understanding of facilitators, barriers and demographics influencing blood and organ donation (Barnieh et al., 2017; Klinkenberg et al., 2019), but this does not exist for USCD. Existing systematic reviews on stem cell donation have focused solely on related donors (Zomerdijk et al., 2019) or have synthesized qualitative studies of related and unrelated donors together (Garcia et al., 2013). However, relative to related donors, unrelated donors have various motivations towards donation and weigh the costs and benefits of donation from different perspectives (Switzer et al., 1999; Zomerdijk et al., 2019).

In addition to the need to understand facilitators and barriers influencing USCD, there is a need to ensure this understanding is theoretically informed and therefore more likely to be useful in developing effective evidenced‐based interventions. The Behaviour Change Wheel (BCW; Michie et al., 2014) is a theoretical model for understanding the causal mechanisms that drive behaviour and provides a structured process for mapping these on to intervention content to change behaviour. The first component in the BCW for understanding the causal mechanisms of behaviour includes the COM‐B Model. The COM‐B model proposes that behaviour is the result of three interacting factors; capability (knowledge, skills, and abilities to engage in a behaviour), motivation (brain processes which drive our decisions and behaviours, this includes habitual processes, emotional responding, as well as analytical decision making) and opportunity (social and environmental factors which make the behaviour possible) (Michie et al., 2011). The COM‐B model has been widely used across health literature to assess facilitators and barriers influencing health‐related behaviour, including blood and organ donation (Ferguson et al., 2019; Marshall et al., 2019; NICE, 2014). The BCW has two outer layers that map on the COM‐B: ‘Intervention Functions’, which can address deficits in capability, opportunity, motivation, and behaviour and ‘Policy Categories’ which can be used to deliver intervention functions (Michie et al., 2014).

This systematic review has focused on the use of the terms facilitators and barriers to describe variables that influence stem cell donation behaviours. In the wider literature, facilitators and barriers are commonly grouped together under the term factors. This term can be exceedingly ambiguous with the same factor having both a positive and negative effect in relation to the behaviour. Facilitators and barriers identified in the study will be mapped to the COM‐B model to identify causal mechanisms that underpin USCD behaviour. Mapping of the findings to ‘Intervention Functions’ and ‘Policy Categories’ as part of the wider BCW will allow an evidence‐based understanding of facilitators and barriers that influence USCD behaviour and will provide theory and evidence‐based recommendations which can be used by recruitment drives to elicit targeted behaviour change in USCD to improve registry recruitment and retention of donors.

## AIM OF REVIEW

The aim of this systematic review is to provide the first comprehensive understanding of the evidence‐base for facilitators and barriers influencing unrelated stem cell donation.

The research questions are as follows:

1. What are the demographic variables influencing unrelated stem cell donation (USCD: joining a stem cell registry and completing stem cell donation), and leaving a stem cell registry (attrition) and do unrelated younger adults, males and individuals from ethnic minority groups differ in relation to facilitators and barriers to USCD and attrition?
2. What are they key facilitators and barriers influencing unrelated donors joining a stem cell registry, completing stem cell donation and leaving a stem cell registry (attrition) and how do these relate to the COM‐B model?
3. Based on the mapping of facilitators and barriers aligned to COM‐B components to the ‘Intervention Functions’ and ‘Policy Categories’ of the Behaviour Change Wheel (BCW), what are the theory‐ and evidence‐based recommendations for improving the design of future interventions to elicit targeted behaviour change in individuals and groups to increase USCD and improve retention on the register?

## METHOD

### Design

A mixed methods integrated Joanna Briggs Institute (JBI) systematic review was conducted to answer the research questions and reported in accordance with PRISMA guidelines (Lizarondo et al., 2019; Moher et al., 2009). Systematic reviews provide a summary of the medical literature by synthesizing the results of multiple primary studies (Gopalakrishnan & Ganeshkumar, 2013). The review was preregistered at [https://www.crd.york.ac.uk/prospero/display_record.php?ID=CRD42020186317].

### Inclusion criteria

Inclusion and exclusion criteria are viewed fully in File S1 and are constructed by considering the Population, Phenomena of Interest and Context, and Types of Studies.

The population were adults (≥18 years of age) of any gender who either (i) had not yet registered to become an unrelated donor, (ii) had registered to become an unrelated stem cell donor (but had not yet donated) and (iii) had donated stem cells for the first time and were able to describe their experiences retrospectively. Potential volunteer donors must have passed a minimum age established by law or their 18th birthday when no regulation exists (WMDA, 2020).

The phenomena of interest included studies focused on any USCD behaviour. Donation behaviour encompasses joining the register, donation, and attrition from the register. Here, this includes intention and willingness to register, donate, and leave the register. Attrition is generally measured at two different stages, one after initial registration and the final stage close to donation. Existing literature focuses heavily on donation beliefs which are primarily intention to donate and willingness to donate. Although these are not behaviour, intention and willingness to donate have been significantly associated with donation (Hyde & White, 2013; Lindsey, 2005) and so will be used as proxies for behaviour in this review. Due to the large number of different terms used by each study to measure donation‐related behaviour, to compile the information, intention to register, intention to donate and willingness to register were merged as ‘intention to donate’. Inclusion was limited to studies that investigate the facilitators and barriers influencing USCD up to and including retrospective experiences of first‐time donation. Facilitators and barriers have been used, instead of the term factors, to improve clarity and provide a better understanding of the mapping to the COM‐B model.

Regarding context, this review considered studies that investigated the facilitators and barriers influencing USCD in all countries worldwide. Studies that have been conducted in both low‐ and high‐income countries were considered. Studies conducted across a range of different health care and non‐health care settings were considered.

The types of studies considered for inclusion were empirical studies endorsing first person accounts and peer reviewed literature to support identification of studies of high methodological quality where possible. The study designs included were (i) quantitative (observational, quasi‐experimental and experimental research), (ii) qualitative (structured/unstructured interview, open‐ended survey questions and focus groups), and (iii) mixed method studies (provided quantitative and qualitative data components could be extracted separately). Based on the specific research questions, the listed eligible designs were deemed to enable the researchers to synthesize data from similar data sets and findings. Intervention studies, grey literature, and conference proceedings were excluded. Systematic reviews, expert reviews, case studies and opinion papers were excluded. Due to the scale of the search and the limited resources available to the team to support translation studies not in English were excluded. Studies published in English from 1980 to present were included as bone marrow and stem cell transplantation became globally practised during this time.

### Search strategy

First, an initial limited search of MEDLINE and CINAHL was undertaken to identify articles on the topic. An analysis of the text words used in the titles and abstracts of relevant articles alongside the index terms used to describe the articles were collated. These were used to develop the full search strategy for MEDLINE (Ovid), EMBASE (Elsevier), PsycINFO (Ovid) and CINAHL (EBSCOhost). The search strategy (File S2) was adapted for each database and the formal searches were undertaken on 24.07.2020 and updated on the 25.02.2021. Every effort was taken to contact authors via email for unretrievable studies.

### Study selection

All identified citations were collated and uploaded to EndNote Version X9 (Clarivate Analytics). Duplicate records were removed, and the citations were exported to online software tool Rayyan for screening (Ouzzani et al., 2016). A two‐step screening method was followed initially focusing on the titles and abstracts followed by a full text screening. This was completed by two independent reviewers (J.F. and P.R.) for assessment against the inclusion criteria for the review. Any disagreements that arose between the independent reviewers at each stage of the study selection process were resolved through discussion, or with additional reviewers (L.G.W.). Citation and reference list harvesting were completed on all included studies.

### Assessment of methodological quality

The methodological quality of the included studies were assessed independently by two reviewers using the platform JBI SUMARI (Lockwood et al., 2017). The methodological tools and full assessment of included studies are viewed fully in File S5. Eligible quantitative and qualitative studies selected for retrieval were assessed by two independent reviewers for methodological validity using the appropriate JBI Critical Appraisal Checklist for each study design (Moola et al., 2020; Tufanaru et al., 2020). Any disagreements that arose between the reviewers (J.F. and P.R.) were resolved through discussion or resolved by the third reviewer (L.G.W.). Data extraction and meta‐aggregation were carried out on all studies, regardless of their methodological quality. The quality of the available data has been considered in the discussion section of this review.

### Data extraction

Quantitative and qualitative data were extracted using a modified version of the JBI data extraction tool (Lockwood et al., 2017). Specific data about the populations, donation stage of participants, study methods, phenomena of interest, context, and findings related to facilitators, barriers and demographic variables influencing donation intention and behaviour were extracted. The data extraction was completed for each study by one reviewer (J.F.) and cross checked by another reviewer (P.R.).

Quantitative data extracted comprised of descriptive data and results of inferential statistical tests. Effect sizes and p values were extracted to indicate whether results were statistically significant and the size of effect. Where possible, univariate analyses for individual predictors were extracted to provide unadjusted effect sizes (e.g., correlation coefficients) to enable comparability across studies. When this was not possible, adjusted effect sizes (e.g., beta coefficients) were reported. Qualitative data extraction comprised of themes and subthemes with corresponding illustrative quotes and was assigned a level of credibility (unequivocal, credible, or not supported) by the reviewers (J.F. and P.R.) according to JBI methodology (Lockwood et al., 2020). Illustrative quotes were extracted to provide context when themes did not convey meaning. To facilitate the integration of data, the outcome measures ‘willingness to donate/register’ and ‘intention to donate/register’ were merged. This was completed by two reviewers and the data were checked for accuracy, consistency, and rigour by a third reviewer. Any disagreements that arose between the reviewers (J.F. and P.R.) were resolved through discussion and the authors of studies were contacted to request missing or additional data, when required.

### Data transformation

The quantitative data were ‘qualitized’ (Moola et al., 2020). Quantitative data‐based outcomes and statistical tests were transformed into textual description and narrative interpretation by the reviewers (J.F. and PR.). Qualitizing quantitative data is less error prone than codifying qualitative data (Lizarondo et al., 2019). The data was checked for accuracy, consistency, and rigour by a third reviewer. The authors used ‘intention to donate’ as a proxy for synthesized findings on facilitators and barriers that influenced people's attitude (willingness) towards or intention to register or donate.

### Data meta‐aggregation

The convergent approach for integration and synthesis of mixed methods systematic reviews was followed which allows qualitative and quantitative data to be combined towards reaching a more comprehensive understanding (Lizarondo et al., 2019). Qualitized findings were assembled into categories with qualitative findings, based on similarity of meaning. The findings were then aggregated across categories to form synthesized findings. To develop a theory‐ and evidence‐based understanding of the facilitators and barriers influencing USCD behaviour, this part of the analysis was informed by the COM‐B model with categories of findings mapped to the COM‐B components. Repeated, detailed examination of the assembled categories of findings was undertaken by two authors to map the integrated findings to the relevant COM‐B component. Demographic variables were not mapped to the COM‐B. The structure of the synthesis of assembled data to provide integrated findings follows JBI guidance (Lizarondo et al., 2019).

Mapping to the COM‐B model allowed the use of the BCW to examine possible intervention functions and policy categories to provide theory‐ and evidence‐based suggestions to change future USCD behaviour. The COM‐B model informed the aggregation (Michie et al., 2014). Each stage of the data meta‐aggregation process was completed by two reviewers and the data was checked for accuracy, consistency, and rigour by a reviewer. Demographic facilitators and barriers are presented outside of the COM‐B model in Table 1.

### Mapping to the Behaviour Change Wheel

For each COM‐B component, there is a corresponding intervention function that is most likely to be effective in changing behaviour (Michie et al., 2014). Policies are decisions made by authorities that support the delivery of intervention functions. The relationship between COM‐B components and intervention functions, and intervention functions and policy categories, are evidenced in the matrices developed by Michie et al. (2014). The meta‐aggregation process and mapping to the COM‐B model enables an understanding of the targeted behaviour. To develop theory‐ and evidence‐based recommendations for future USCD interventions, intervention functions and policy categories were also identified. Behavioural analysis and diagnosis regarding USCD was undertaken by one author (author's initials removed) and corroborated by the wider research team. The matrices (Michie et al., 2014) were first used to map each COM‐B component to the relevant intervention function and policy category. As recommended by Michie et al. (2014), to select which intervention functions and policy categories are most appropriate to elicit behaviour change, their Affordability, Practicality, Effectiveness and cost effectiveness, Acceptability, Side effects/safety, and Equity (APEASE) were evaluated. The final stage of interpreting behaviour change involves formulating content and interventions options based on identifying specific behaviour change techniques and mode of delivery related to local context (Michie et al., 2014). This is outside the scope of this systematic review, as it related to designing and interpreting behaviour change over time in a local context.

### Changes since pre‐registration of systematic review

Due to the quantity of studies, it was not feasible to investigate facilitators and barriers influencing second time donation. Additionally, both reviewers (J.F. and P.R.) noted the facilitators and barriers influencing second time donation were categorically different, incorporating previous experience, in comparison to facilitators and barriers influencing first time donation. For qualitative studies, the findings were extracted as the facilitators and barriers were not adequately represented at the theme level.

## RESULTS

### Study inclusion

**FIGURE 1:**
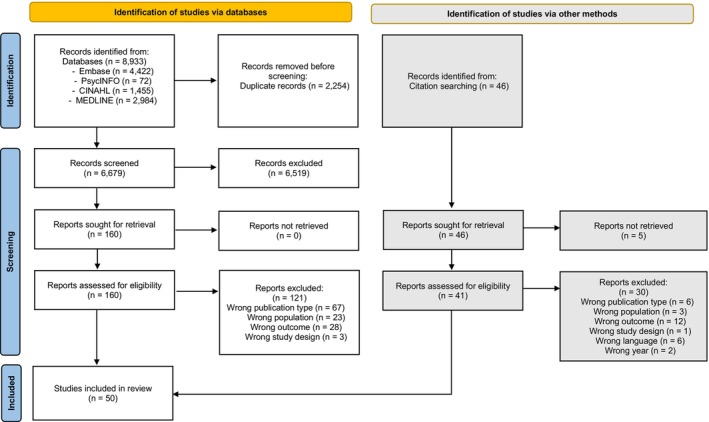
PRISMA 2020 Flow Diagram. From: Page et al., 2021.

The search identified 6679 potentially relevant articles after the removal of duplicates (Figure 1). After screening titles, abstracts, and full text articles, 39 studies met the inclusion criteria and were included in the review. A further 11 studies met the inclusion criteria following citation searching of the original included studies. A total of 50 studies were included in the review.

### Characteristics of included studies

The characteristics and main findings of the 50 included studies are summarized in File S3. Of these, 16 studies recruited participants already registered as potential USCD, and five recruited participants who had already donated stem cells. The remaining studies recruited blood donors (five studies), students (13 studies) or the general public (11 studies). The majority of participants in these studies were not on a registry but did meet the eligibility criteria for their national registry in terms of age (generally under 40 years old) and health status, with the exception being Onitilo et al. (2004) where most were over 50 years old. 

The majority of studies were conducted in the US (26/50), followed by the United Kingdom (5/50). A detailed breakdown of the country of origin of all studies can be viewed in File S4. There were 44 quantitative studies of which 34 were cross sectional, five randomized control trials, two retrospective, one longitudinal, one quasi‐experimental and one case control. There were six qualitative studies, five were thematic analysis and one grounded theory. The studies included measured a variety of stages of donation, including beliefs and intentions. The studies could be divided into three broad categories: 31 studies on behaviour, 19 studies on proxies for behaviour and 12 studies focused on registered donors. The behaviour most focused on was joining the register with 14 studies, eight studies focused on donation behaviour, five studies on motives for registration and four on attrition from the register. The studies on proxies for behaviour included six on intention to donate, five on willingness to donate, four on intention to register, three on availability after joining the register and one on willingness to join.

### Methodological quality

The methodological quality of studies ranged from low to high, of which 28 studies were of high methodological quality and 17 were medium methodological quality. Five studies were of low quality and were kept in the review. The majority of findings from low quality studies were consistent with results of higher quality studies, therefore the authors are confident the low quality did not influence the results. Additionally, this was a large review with the majority of studies being of higher quality.

### Findings

#### Integrated findings

There are four overall integrated findings which are based on a merged summary of all the available evidence from the studies in the review and related to the three behaviours listed above. Demographics sit outside of the COM‐B model and are presented in Table 1. Tables 2, 3, 4 report the integrated findings alongside the qualitative and quantitative significant review findings, framed by the COM‐B model. Facilitators and barriers influencing all three behaviours have been classified under each section of the COM‐B model. Refer to the tables for the comprehensive mapping of all facilitators and barriers which may not be explicitly mentioned in the text results, each table used is presented in File S6. The integrated findings are presented and mapped to the COM‐B model, to allow theory‐ and evidence‐ based suggestions for behaviour change to be made using the BCW.

**TABLE 1:** Integrated Finding 1 in relation to Demographic Variables and the related facilitators and barriers synthesized influencing unrelated stem cell donation (including joining a registry, donation and attrition from a registry) identified from published literature.

Integrated finding 1	Demographic facilitators/barriers	Findings from Qualitized (QZ) and qualitative (QT) data
Predictors of intention to donate were: being married, being a college student or having a higher level of educational attainment. Barriers associated with higher levels of attrition appear to be older age and unemployment.	Age Facilitator	**QZ** Being younger was associated with a greater willingness (Abdrbo et al., 2017; Briggs et al., 1986; Kwok et al., 2015; Milaniak et al., 2020; Onitilo et al., 2004). **QZ** Being older was associated with a greater willingness (Hazzazi et al., 2019; Sarason et al., 1993).
	Age Barrier	**QZ** Older people had higher attrition rates overall at the initial stage after registration (Monaghan et al., 2021) and the final stage closer to donation (Lown et al., 2014). **QZ** Older people had higher attrition rates on medical grounds at the final stage closer to donation (Balassa et al., 2019). **QZ** Attrition on personal grounds was more common in the 30–40 age group at the final stage closer to donation (Balassa et al., 2019).
	Gender Facilitator	**QZ** Being female was associated with a greater willingness (Galanis et al., 2008; Narayanan et al., 2016; Studts et al., 2010). **QZ** Being male was associated with a greater willingness (Briggs et al., 1986).
	Gender Barrier	**QZ** Females had higher rates of attrition overall and on medical grounds at the final stage closer to donation (Lown et al., 2014).
	Ethnicity Facilitator	**QZ** Attitude predicted intention to donate more strongly for Black Americans than for White Americans but similarly for Hong Kong Chinese and American Chinese (Bagozzi et al., 2001).
	Ethnicity Barrier	**QZ** Non‐white people had higher rates of attrition than white people at the initial stage after registration (Switzer et al., 2004) and the final stage closer to donation (Lown et al., 2014; Switzer et al., 2004). **QZ** Africans or African‐Caribbean groups had higher rates of attrition than White northern Europeans and other ethnic groups at the final stage closer to donation (Lown et al., 2014). **QZ** Asian, Jewish, and Mediterranean groups had higher rates of attrition than White northern European groups at the final stage closer to donation (Lown et al., 2014). **QZ** African and African Caribbean groups had higher rates of being non‐contactable than white northern Europeans and Asians the final stage closer to donation and subsequent attrition (Lown et al., 2014). **QZ** Asians had higher rates of attrition for personal reasons and lower rates of attrition on medical grounds at the final stage closer to donation (Lown et al., 2014). **QZ** Those who considered that their ethnicity was a facilitator in their decision to register had higher rates of attrition after the initial stage of registration (Switzer et al., 1999, 2004). **QZ** Ethnic minorities were more likely to have religious objections to donation and mistrust in how the HSC would be used than white people (Anthias et al., 2020; Switzer et al., 2013). **QT** *Ethnic background* “The lack of ethnic minority donors and my irritation that there was stigma attached to donating blood in the African community.” P.428 *Unequivocal* (La Casta et al., 2019). In relation to donor registration.
	Marital Status Facilitator	**QZ** Being married was associated with a greater willingness (Abdrbo et al., 2017; Glasgow & Bello, 2007; Studts et al., 2010). **QZ** Being married was associated with lower rates of attrition at the second stage of donor registration (Switzer et al., 2004).
	Family Facilitator	**QZ** Having children was associated with a greater willingness (Galanis et al., 2008). **QZ** Having no or few children was associated with a greater willingness (Briggs et al., 1986). **QZ** Having siblings was associated with a greater willingness (Tuszynska‐Bogucka, 2019). **QZ** Having higher family affection was associated with greater willingness (Tuszynska‐Bogucka, 2019).
	Education Facilitator	**QZ** A higher level of education was associated with a greater willingness (Galanis et al., 2008; Hazzazi et al., 2019; Kwok et al., 2015; Laver et al., 2001; Onitilo et al., 2004; Tuszynska‐Bogucka, 2019).
	Personality Facilitator	**QZ** High conscientiousness was associated with greater willingness (Tuszynska‐Bogucka, 2019).
	Blood Donor Facilitator	**QZ** Willingness was associated with being a blood donor (Beatty et al., 1989; Galanis et al., 2008; Hazzazi et al., 2019; Narayanan et al., 2016; Norvilitis & Riley, 2001). **QZ** 7.8% cited ‘being a blood donor’ as a motivation (Aurelio et al., 2011). **QZ** Willingness was associated with having fewer previous blood donations (Briggs et al., 1986) and no experience of apheresis (Briggs et al., 1986; McCullough et al., 1986). **QZ** Attrition rates were lower among blood donors at the initial stage after registration (Switzer et al., 1999, 2004) and the final stage closer to donation (Anthias et al., 2020; Lown et al., 2014; Monaghan et al., 2021). **QZ** Being a registered organ donor was associated with greater intention to donate (Glasgow & Bello, 2007).
	Blood Donor Barrier	**QZ** Having a poorer blood donor reliability score was associated with attrition at the final stage closer to donation, both overall and on personal grounds (Balassa et al., 2019). **QZ** Being a blood donor was associated with less willingness (McCullough et al., 1986). **QZ** Having experienced pain or side‐effects as result of giving blood is associated with unwillingness to join registry (Hyde et al., 2014).
	Income Barrier	**QZ** Lower income was associated with higher rates of attrition at a late stage of the donation process (Switzer et al., 2013).
	Occupation Facilitator	**QZ** Willingness was associated with being a college student (Abdrbo et al., 2017); and having a professional rather than clerical or manual occupation (Aurelio et al., 2011).
	Occupation Barrier	**QZ** Being unemployed was associated with higher attrition at the final stage closer to donation (Anthias et al., 2020; Switzer et al., 2013).

*Note*: Integrated Findings are a merged summary of all the available evidence from studies in the review on facilitators and barriers to donation‐related behaviour (joining the register, donation and attrition from the registry).

Qualitized (QZ)–Quantitative data‐based outcomes and statistical tests were transformed into textual description and narrative interpretation.

Qualitative (QT)–Qualitative data comprised of themes and subthemes taken from qualitative studies included in the systematic review.

**TABLE 2:** Integrated Finding 2 in relation to the Capability component of the COM‐B model and the related facilitators and barriers synthesized influencing unrelated stem cell donation (including joining a registry, donation and attrition from a registry) identified from published literature.

Integrated finding 2	COM‐B categories‐capability	Facilitators/barriers	Findings from Qualitized (QZ) or qualitative (QT) data
Donation‐related knowledge was a facilitator of intention to donate, joining the registry, and donation of USCD. Lack of donation‐related knowledge was a barrier to joining the registry and donation and led to attrition from the registry.	Capability was defined as the individual's psychological and physical capacity to engage in the activity of USCD (Michie et al., 2011). Having the necessary knowledge was part of psychological capability.	Knowledge Facilitator	**QZ** Greater knowledge was associated with greater willingness (Abdrbo et al., 2017; Galanis et al., 2008; Hazzazi et al., 2019; Kwok et al., 2015; Norvilitis & Riley, 2001; Sikora et al., 2014; Vasconcellos et al., 2011). **QZ** Knowledge about the following specific donation‐related issues was associated with greater willingness: the sample required (Hazzazi et al., 2019); bone marrow transplantation (BMT) process and risks (Hyde et al., 2014); donor eligibility (Narayanan et al., 2016); the need to be registered before donating (Ting et al., 2020); side‐effects (Hazzazi et al., 2019; Narayanan et al., 2016; Ting et al., 2020); that BMT saves lives (Laver et al., 2001; Onitilo et al., 2004); that ethnicity of donors is important (Laver et al., 2001); knowing what is the National Marrow Donation Programme (NMDP) (Onitilo et al., 2004).
		Knowledge Barrier	**QT** A lack of knowledge acted as a barrier to recruitment to the registry and contributed to fear about the process “you can make the procedure well known so people know what they are getting into and aren't so afraid to do it.” p.7 *Unequivoca*l (Kaster et al., 2014). **QZ** 64.1% of those unwilling to donate cited inadequate knowledge as a barrier (Kwok et al., 2015). **QZ** 42.5% of those unwilling to donate cited lack of awareness of the registry as a barrier (Abdrbo et al., 2017). **QZ** 52.2% of those unwilling to donate and 44.6% of those willing to donate but not register cited inadequate knowledge as a barrier (Abdrbo et al., 2017). **QZ** 33.3% of those unwilling to donate cited ‘not knowing how’ as their reason (Bagcivan et al., 2020). **QZ**. 14.9% of students who did not take part in an on‐campus drive said they were unaware of the need for bone marrow donors (Norvilitis & Riley, 2001). **QZ** ‘Lack of information on donation’ and ‘lack of information on risks’ were the two highest ranked barriers to enrolment (Bart et al., 2014).
		Information Facilitator	**QZ** The belief that they ‘know more about how to register’ was associated with willingness to join registry (Hyde et al., 2014).
		Information Barrier	**QZ** Attrition was associated with feeling ‘less informed’ (Switzer et al., 2004). Attrition from both the initial stage after registration and the final stage closer to donation. **QZ** Those who believed they had not received adequate information from registry staff had a higher rate of attrition (Switzer et al., 2004). Attrition from both the initial stage after registration and the final stage closer to donation. **QZ** Attrition from the register after the initial stage was associated with ‘not remembering joining when contacted’, and ‘not knowing why they were contacted’ (Switzer et al., 2004). **QZ** Attrition at a late stage of the donation process was higher among those who felt staff did not explain the BMT process (Anthias et al., 2020). **QT**. *Ability to withdraw* “Potential registrants asked if they could say “no” if they ever matched with a patient (Dasgupta, 2018)”. This alleviated anxiety and provided reassurance for potential new registrants.
		Mental Health Barrier	**QZ** Willingness was associated with lower personal distress (Milaniak et al., 2020). **QZ** Attrition at the final stage close to donation was higher among those with poorer mental health‐related quality of life **(**Anthias et al., 2020; Switzer et al., 2013); higher depression (Switzer et al., 2004, 2013); and higher anxiety (Switzer et al., 2013).

*Note*: Integrated Findings are a merged summary of all the available evidence from studies in the review on facilitators and barriers to donation‐related behaviour (joining the register, donation and attrition from the registry).

Qualitized (QZ)–Quantitative data‐based outcomes and statistical tests were transformed into textual description and narrative interpretation.

Qualitative (QT)–Qualitative data comprised of themes and subthemes taken form qualitative studies included in the systematic review.

**TABLE 3:** Integrated Finding 3 in relation to the Opportunity component of the COM‐B model and the related facilitators and barriers synthesized influencing unrelated stem cell donation (including joining a registry, donation and attrition from a registry) identified from published literature.

Integrated finding 3	COM‐B categories‐opportunity	Facilitators/barriers	Findings from Qualitized (QZ) or qualitative (QT) data
Perceived convenience was a facilitator of registration. Positive social influences, religious beliefs and the accessibility of positive donation‐related social norms promoted behavioural intention to donate, joining the register and donation of USCD. Physical barriers such as cost, lack of time and inconvenient location were obstacles to registration.	Opportunity was defined as the factors outside the individual that make the behaviour possible or prompt it (Michie et al., 2011). Opportunity was distinguished into physical opportunity afforded by the environment and social afforded by the cultural surroundings that dictates the way that one thinks about things.	Time on registry Barrier	**QZ** Being on the registry longer was associated with higher attrition at the initial stage after registration (Switzer et al., 1999) and the final stage closer to donation (Lown et al., 2014).
		Lack of time Barrier	**QZ** ‘Lack of time’ was the fourth highest ranked barrier to enrolment (Bart et al., 2014). **QZ** 52% of medical students said that the time commitment involved was a concern (Narayanan et al., 2016). **QZ** 38.3% selected ‘lack of time’ as the reason they did not take part in an on‐campus drive (Norvilitis & Riley, 2001). **QZ** The belief ‘too many constraints on my time’ was associated with lower intention (Hyde et al., 2014).
		Ineligibility Barrier	**QZ** 26.6% of registrants cited ‘waited to reach adequate age’ as a prior barrier (Branach et al., 2018).
		Cost Barrier	**QZ** 25% of medical students said that financial cost associated with USCD was a concern (Narayanan et al., 2016).
		Opportunity Barrier	**QZ** 50% of registrants cited ‘lack of possibility’ as a prior barrier (Branach et al., 2018).
		Convenience Facilitator	**QT** *Facilitators* “ease and convenience important factors during registration” p.6 *Credible* Kaster et al. (2014). **QT** *Facilitators* “it would have to be made easier for me to be able to be a donor” p.6 *Unequivocal* Kaster et al. (2014). This refers to joining the registry and follow through process with donation once on the register. **QT** *It was convenient* “If they weren't there that day I probably wouldn't have joined up” p.428 *Unequivocal* La Casta et al. (2019). In relation to donor registration. **QZ** 21.2% of students who took part in a drive cited ‘convenience’ as a motivation (Norvilitis & Riley, 2001).
		Convenience Barrier	**QZ** Unwillingness to join registry was associated with the belief that a high amount of effort is required (Hyde et al., 2014).
		Geographic location Facilitator	**QZ** Registering at a community drive was associated with lower attrition from the registry at the initial stage of the donation process (Switzer et al., 2004).
		Geographic location Barrier	**QZ** Attrition varied by region in China. The eastern region had the lowest rate and North‐Eastern the highest. The proportion that received information and education was lower in the North‐East compared to other regions (Li et al., 2021). **QZ** Attrition during the initial stage after registration was associated with registering at a recruitment event (Monaghan et al., 2021). **QZ** Attrition at a late stage of the donation process was associated with registering at an educational institution (Anthias et al., 2020; Switzer et al., 2004). **QZ** 8.5% of those who did not take part selected the barrier ‘inconvenient location’ (Norvilitis & Riley, 2001).
		Communi‐cation with registry Facilitator	**QT** *Recruiting strategies* the use of persuasion ‘“participants felt persuading potential donors was pertinent “people at the drive that [they] finally convinced to sign up, that were hesitant”’ p.5 Unequivocal. (Kaster et al., 2014).
		Communi‐cation with registry Barrier	**QZ** Attrition is associated with feeling pressured when contacted about matching; not receiving informational mailings; and not receiving information about the recipient (Switzer et al., 2004). Attrition from both the initial stage after registration and the final stage closer to donation. **QZ** Attrition at a late stage of the donation process was associated with less reported contact with registry staff (Anthias et al., 2020). **QZ** Attrition during the initial stage after registration was associated with registering at: a drive for a specific patient (Switzer et al., 1999, 2004)
	
		Type of Donation Barrier	**QZ** Peripheral blood stem cell as the stem cell source was associated with higher rates of attrition at the final stage closer to donation for any reason (and on medical and personal grounds) compared to bone marrow as the stem cell source (Balassa et al., 2019).
		Friends & Family influence Facilitator	**QZ** Previously discussing tissue or organ donation with family was associated with willingness to join the registry (Galanis et al., 2008). **QZ** 12.1% said they attended a drive because a friend was attending (Norvilitis & Riley, 2001). **QZ** 8.1% of registrants said that family or friends influenced their decision (Branach et al., 2018). **QZ** The motive ‘accompanied by a friend or relative’ was ranked 8 out of 12 possible motives by registered donors to join the registry (Bart et al., 2014). **QZ** Willingness was associated with attending a drive with friends (Norvilitis & Riley, 2001). **QZ** Willingness to join the register was associated with having a friend or relative already registered (Galanis et al., 2008). **QT** *Friends and peer influence* “My boyfriend had the same feeling, 2 or 3 months later we both decided to donate.” P.36 *Unequivocal*, (Holroyd & Molassiotis, 2000). **QT** *Encouraged by friends/family* “A work colleague had joined, and he suggested it to me, so I joined” *Unequivocal* p.428 (La Casta et al., 2019) **QT** *Social influence* “From the time I was a little kid, I was indoctrinated‐you had duty.” p. 290 *Unequivocal* (Simmons et al., 1993). **QZ** Being discouraged by others was associated with unwillingness, whereas being encouraged is associated with willingness (McCullough et al., 1986). **QZ** Attrition after the initial stage of registration was lower among those who were encouraged by others (Switzer et al., 1999).
		Friends & Family influence Barrier	**QZ** Family was cited as a barrier by 42.7% (Kwok et al., 2015) and 11.4% (Abdrbo et al., 2017) of those unwilling to donate and by 11.1% of those willing to donate but not register (Abdrbo et al., 2017). **QT** “I tried not to tell my parents…when they found some mail, then they did their best to dissuade me.” *Unequivocal*, (Holroyd & Molassiotis, 2000). **QT** *Being pressured* “I think I felt quite pressured and by the people going around asking to donate.” p.429 *Unequivocal* (La Casta et al., 2019). In relation to donor registration. **QZ** Attrition is higher among those discouraged from joining by family, friends or health care professionals (HCPs) at the initial stage after registration (Switzer et al., 1999) and the final stage closer to donation (Anthias et al., 2020; Switzer et al., 2004). **QZ** Attrition after the initial stage of registration was higher among those who had not discussed their decision to register with relatives nor with HCPs and among those who had joined with others (Switzer et al., 1999).
		Subjective norms Facilitator	**QZ** Willingness to donate was associated with ‘lower perceived importance of approval of others’ (Glasgow & Bello, 2007), higher perceived subjective norm (Hyde & White, 2013; Ting et al., 2020) and perceived injunctive norm (Lee‐Won et al., 2016) and the belief that others approve (Hyde et al., 2014; Ting et al., 2020). **QZ** Higher social desirability was associated with greater willingness (Norvilitis & Riley, 2001). **QZ** Subjective norms predicted intention to donate for Hong Kong and American Chinese and White Americans but not for Black Americans (Bagozzi et al., 2001). **QZ** 26% cited normative motives as their reason for registering (Switzer et al., 1997).
Subjective norms Barrier	**QZ** External influences were associated with intention not to donate bone marrow (Glasgow & Bello, 2007). External influences include family, friends, church members and the church opinions and thoughts.
	
		Media influence/ response to a campaign Facilitator	**QZ** 54.5% of students who registered were influenced by signs around campus (Norvilitis & Riley, 2001). **QZ** 12.9% of registrants were influenced by a public campaign (Branach et al., 2018). **QZ** The motive ‘approached by donor centre’ was ranked 6th out of 12 possible motives by registrants to join the registry (Bart et al., 2014). **QT** *Public messages and social identity informing the decision to donate* “We all talked about the plight of Little Gordon, at school, it was so sad” p.34 *Unequivocal* (Holroyd & Molassiotis, 2000). **QT** *Responded to a public appeal* “His parents were on the television and appealing to people to go and join, so I went.” p. 428 *Unequivocal* (La Casta et al., 2019). In relation to donor registration.
		Media influence/response to a campaign Barrier	**QT** *Media, health professionals and work* “unfavourable newspaper coverage of the safety of hospital procedures had caused them concerns”. p.35 *Credible* (Holroyd & Molassiotis, 2000).
		Religious Beliefs Facilitator	**QT** *Personal values* “To save another human life regardless of race, colour, ethnicities, and religion which is taught by my religion” p. 428 *Unequivocal* (La Casta et al., 2019). In relation to donor registration. **QT** *Self‐image as a member of a religious group* “I can carry out my Christian ethic.” p. 291 *Unequivocal (*Simmons et al., 1993). **QT** *Religious messages* “I was taught by Jesus that I had to affect the life of others.” *Unequivocal* (Holroyd & Molassiotis, 2000). **QT** *Religious identification and the decision to donate* “I teach girls in a catholic school and teach them to try and save lives” p.8 *Unequivocal* (Billen et al., 2017). **QZ** ‘In accordance with my principles’ was ranked as the fifth most important motivation for enrollment (out of 12) (Bart et al., 2014). **QZ** Religious beliefs were a motivation for 1.2% (Aurelio et al., 2011) and 2.4% of registrants (Branach et al., 2018). **QZ** Willingness was associated with the belief that personal or religious beliefs influenced the decision to register (McCullough et al., 1986). **QZ** Being a Methodist was associated with intention to donate (Glasgow & Bello, 2007). **QZ** Willingness to join registry was associated with having higher moral norms (Hyde & White, 2013). **QZ** Reporting social or religious motives was associated with lower rates of attrition after the initial stage of registration (Switzer et al., 1999).
Religious Beliefs Barrier	**QZ** Religion was cited as a barrier by 6.3% (Kwok et al., 2015) and 4.5% (Abdrbo et al., 2017) of those unwilling to donate and by 4.3% of those willing to donate but not register (Abdrbo et al., 2017). **QZ** Having religious objections is associated with higher rates of attrition at the initial stage after registration (Switzer et al., 1999) and the final stage closer to donation (Anthias et al., 2020; Switzer et al., 2013).

*Note*: Integrated Findings are a merged summary of all the available evidence from studies in the review on facilitators and barriers to donation‐related behaviour (joining the register, donation and attrition from the registry).

Qualitized (QZ)–Quantitative data‐based outcomes and statistical tests were transformed into textual description and narrative interpretation.

Qualitative (QT)–Qualitative data comprised of themes and subthemes taken form qualitative studies included in the systematic review.

**TABLE 4:** Integrated Finding 4 in relation to the Motivation component of the COM‐B model and the related facilitators and barriers synthesized influencing unrelated stem cell donation (including joining a registry, donation and attrition from a registry) identified from published literature.

Integrated finding 4.	COM‐B categories–Motivation	Facilitators/barriers	Findings from Qualitized (QZ) or qualitative (QT) data
Altruism was the most commonly cited motive for intention to donate, joining the registry and donation. Sense of duty, often stemming from religious, cultural, or familial values, was associated with intention to donate, joining the register and donation in a large number of studies. Fear and health‐based concerns were barriers to USCD. Motivations to donate ranged from intrinsic to extrinsic. Non‐intrinsic motivation appears to be associated with later attrition.	Motivation was defined as all those brain processes that energize and direct behaviour and conscious decision‐making (Michie et al., 2011). This is divided into reflective motivation (involving evaluations and plans) and automatic motivation (involving emotions or impulses from associative learning and/or innate dispositions).	Altruism Facilitator	**QT** *Desire to save a life* “A small act on my part can save someone's life.” p. 428 *Unequivocal* (La Casta et al., 2019). In relation to donor registration. **QT** *Intrinsic motivation‐altruism* “I just wanted to help someone” p.7 *Unequivocal* (Billen et al., 2017). This refers to post‐donation beliefs. **QT** *Goodness “*Participants felt that [the message] *saving lives* and *helping people* was very critical in encouraging individuals to join” *Credible* p.7 (Kaster et al., 2014 *)*. **QT** *Altruism* “It was simply a chance to save a person” p.36 *Unequivocal* (Holroyd & Molassiotis, 2000). **QZ** Willingness regarding intention to donate was associated with a greater belief in helping others (Glasgow & Bello, 2007). **QZ** 94.4% (Branach et al., 2018), 69.7% (Norvilitis & Riley, 2001), 67.8% (Aurelio et al., 2011), and 37% (Switzer et al., 1997) of registrants and 80% of donors (Stroncek et al., 1989) reported the motive of wanting to help people. **QZ** 71.8% of registrants reported wanting to do a good deed (Branach et al., 2018). **QZ** The motive ‘the prospect of saving lives’ was ranked as the most important motive join the registry and ‘the prospect of increasing a patient's chances’ the third most important reason for enrollment (Bart et al., 2014).
		Sense of Duty/Motive Facilitator	**QT** *Sense of duty* “Someone needs help, and you do it, don't you?” p. 428 *Unequivocal* (La Casta et al., 2019). In relation to donor registration. **QT**. ‘“a moral concern for the state “I have an obligation to save others on the condition that it is a helping action. Being human means that we should love one another.”’ p. 36 *Credible*, (Holroyd & Molassiotis, 2000) **QT** *Recruiter as the recruitee* “I need to join because otherwise I shouldn't be telling other people to join.” p. 4 *Unequivocal* (Kaster et al., 2014). **QT** *Self‐image as fortunate* “I feel like I'm giving something back… I can ease the burden for someone who hadn't had it as fortunate” p. 291 *Unequivocal* (Simmons et al., 1993). **QT** *Sense of duty* “I couldn't live with myself if I didn't go ahead” p.1 *Unequivocal* (Billen et al., 2017). This refers to post donation beliefs. **QZ** The motive ‘sense of duty’ was cited by 63.6% (Norvilitis & Riley, 2001), 5.4% (Aurelio et al., 2011) and 35.5% (Branach et al., 2018) of registrants. **QZ** The motive ‘nobleness of act’ motive was cited by 4.6% of registrants (Aurelio et al., 2011).
		Beliefs about inaction Facilitator	**QZ** Higher induced anticipated guilt was perceived to increase intention to donate (Lindsey, 2005). **QZ** Greater anticipated regret was associated with willingness to join the registry (Hyde & White, 2013).
		Ambi‐valence Facilitator	**QZ** Lower ambivalence was associated with greater willingness to donate after joining the register and donating (Vekaria et al., 2020). **QZ** Lower ambivalence was associated with lower attrition at the early stage after registration (Switzer et al., 2004) and the final stage closer to donation (Anthias et al., 2020; Switzer et al., 2004, 2013;Vekaria et al., 2020).
	
		Ambi‐valence Barrier	**QZ** Higher ambivalence was associated with attrition at the final stage closer to donation (Vekaria et al., 2020). **QZ** Attrition from the register after the initial stage of registration was higher among those who reported delaying the decision to join the register (Switzer et al., 1999), and attrition at a late stage of the donation process among those who report feeling less satisfied with their decision (Switzer et al., 2013). ***Q*T** *Identifying concerns* ‘The process seemed scary’ p. 89 *Unequivocal* (Dasgupta, 2018). **QT** *Fear of indefinite commitment* potential registrants asked if they could say “no” if they ever matched with a patient” or if “they could have more time to think about registering” p.89 *Unequivocal* (Dasgupta, 2018).
		Health concerns Barrier	**QZ** Willingness was associated with: less concern of complications (Vasconcellos et al., 2011): having fewer concerns regarding side‐effects (Hazzazi et al., 2019; Ting et al., 2020). **QZ** Higher perceived risk of the process of USCD was perceived to lead to lower intention to donate in potential registrants (Briggs et al., 1986; Mclaren et al., 2012). **QZ** Health concerns was cited as a barrier by 57.2% for lack of intention to donate (Kwok et al., 2015), 22.7% (Bagcivan et al., 2020) of those unwilling to donate and 41.1% (Abdrbo et al., 2017) of those unwilling to donate and by 35.7% of those not willing to register (Abdrbo et al., 2017). **QZ** 6.4% of registrants cited ‘health concerns’ as a prior barrier (Branach et al., 2018). **QZ** Belief in severe health risks was ranked as the 8th most important, out of 12, barriers to enrolment (Bart et al., 2014). **QZ** Attrition was associated with having more medical‐based concerns at the initial stage after registration (Switzer et al., 1999, 2004) and the final stage of close to donation (Anthias et al., 2020; Switzer et al., 2004, 2013). **QZ** Attrition after the initial stage of registration was higher among those who believed that survival chances were low (Switzer et al., 1999). **QZ** Attrition at the final stage closer to donation was associated with poorer perceived physical health‐related quality of life (Anthias et al., 2020). **QZ** Attrition at a late stage of the donation process was higher among those who believed that donation could result in serious complication (Switzer et al., 2013).
		Fears of the process Facilitator	**QZ** Willingness was associated with less fear of the BMD process (Galanis et al., 2008; Glasgow & Bello, 2007).
		Fears of the process Barrier	**QZ** 32.3% of registrants cited fear of anaesthesia and 14.5% cited fear of infection as a prior barrier (Branach et al., 2018). **QZ** 19.1% identified fear of needles as a barrier to registration (Norvilitis & Riley, 2001). **QZ** 11.3% of registrants cited fear of the donation procedure as a prior barrier (Branach et al., 2018). **QZ** Fear of medical procedures was the third most important barrier to enrolment (Bart et al., 2014). **QZ** Reduced willingness to join registry was associated with fear of blood/needles (Hyde et al., 2014; Norvilitis & Riley, 2001); and fear of medical procedures (Norvilitis & Riley, 2001). **QZ** Higher attrition after the initial stage of registration was associated with fear of pain, needles, and anaesthesia (Switzer et al., 1999).
Trust in the health system Facilitator	**QZ** Greater trust in the health system was associated with willingness to register (Galanis et al., 2008). **QZ** Greater trust in the health system was associated with greater intention to donate (Glasgow & Bello, 2007).
	
		Trust in the health system Barrier	**QZ** Lack of trust in the health system was cited as a barrier by 35.3% (Kwok et al., 2015), and 42.4% (Abdrbo et al., 2017) of those unwilling to donate and by 33.5% of those willing to donate but not register (Abdrbo et al., 2017). **QZ** Mistrust about the fairness of haematopoietic stem cells (HSC) allocation was associated with greater attrition at the initial stage after registration (Switzer et al., 1999) and the final stage closer to donation (Anthias et al., 2020; Switzer et al., 2013) **QZ** Mistrust about the fairness of HSC allocation was associated with lower intention to donate (Ting et al., 2020).
		Negative donation‐related beliefs Barrier	**QT** *Apathy* “why bother spending any time signing up if I am not going to be chosen” p. 7 *Unequivocal* (Kaster et al., 2014). This refers to joining the registry and follow through process with donation once on the register. **QZ** The belief that ‘bone marrow donation (BMD) is an invasion of the body’ was associated with reduced willingness to join the registry (Hyde et al., 2014). **QZ** 25.5% of students who did not take part in a drive gave the reason ‘not interested’ while 10.6% gave the reason ‘lack of belief in the usefulness of BMD’ (Norvilitis & Riley, 2001). **QZ** Attrition was associated with having less realistic donation‐related expectations (Switzer et al., 2004). Attrition from both the initial stage after registration and the final stage closer to donation. Realistic expectations included anticipating 2 or fewer days in hospital and 5 or fewer days missed from work.
		Beliefs about capabilities Facilitator	**QZ** Intention to donate was associated with higher perceived self‐efficacy (Ting et al., 2020), and greater perceived behavioural control (Glasgow & Bello, 2007). **QZ** Intention to join a registry was associated with greater perceived behaviour control (Hyde & White, 2013). **QZ** Low self‐esteem was associated with willingness (Briggs et al., 1986).
		Beliefs about capabilities Barrier	**QZ** Low self‐esteem was associated with higher rates of attrition at the final stage closer to donation (Switzer et al., 2004, 2013). **QZ** Attrition during the initial stage after registration was associated with lower mastery (Switzer et al., 2013). Mastery refers to believing that one has influence over what happens them.
Positive feeling motives Facilitator	**QZ** Positive feeling‐related‐motives were cited by 25% of registrants (Switzer et al., 1997) and by 5% of donors (Stroncek et al., 1989). **QZ** ‘Personal comfort’ was cited as a motivation by 3.9% of registrants (Aurelio et al., 2011). **QZ** ‘Curiosity’ was cited as a motivation by 4% of registrants (Branach et al., 2018) **QZ** 10% of donors donated ‘for the experience’ (Stroncek et al., 1989). **QZ** Attrition after the initial stage of registration was lower among those who reported positive feeling‐related motives (Switzer et al., 1999). **QZ** Receiving more information and receiving a letter praising blood donation work was perceived to lead to greater intention (Sarason et al., 1993). **QT** *Anticipated positive feeling* “My friends had joined the registry previously and told me how rewarding it was” p.428 *Unequivocal* (La Casta et al., 2019). In relation to donor registration. **QZ** Willingness to donate after joining the register and donating was associated with positive thoughts about future donation (Vekaria et al., 2020). Those who opted out at the final stage close to donation were associated with negative thoughts about donation (Vekaria et al., 2020). **QZ** Attrition at the final stage close to donation was lower among those who had positive thoughts about future donation (Vekaria et al., 2020). **QZ** Intention to donate was associated with the belief that HSC is lawful (Ting et al., 2020). **QZ** Positive donation‐related attitudes were associated with the intention to donate (Hyde & White, 2013). **QZ** Willingness was associated with the belief that friends or family would join too (Hyde et al., 2014).
	
		Past personal and family experience Facilitator	**QZ** Willingness was associated with ‘hearing about people who benefited from a transplant’ (Hyde et al., 2014); ‘knowing someone who needs a transplant’ (Galanis et al., 2008; Hyde et al., 2014; McCullough et al., 1986; Studts et al., 2010); having previously donated HSC to family (Hazzazi et al., 2019); and ‘family experience of giving or receiving blood’ (Briggs et al., 1986). **QZ** Knowing someone who had cancer was cited as a motivation by 53.0% of registrants (Norvilitis & Riley, 2001); and 10% of donors (Stroncek et al., 1989). **QZ** Motivation for donation included having previously considered donating were cited by 37.9% (Norvilitis & Riley, 2001). **QZ** Past‐experience based motives for donation were cited by 8% of registrants (Switzer et al., 1997); having experienced family loss by 0.6% (Aurelio et al., 2011); having previous donation to family or friend by 4.8% (Aurelio et al., 2011); having a relative or friend who needed HSCT by 8.1% (Branach et al., 2018) and by 10% (Stroncek et al., 1989). **QZ** ‘Relative/friend needs stem cells’ was ranked 4th out of 12 possible motives for enrollment (Bart et al., 2014). **QT** *because of the illness in the past of someone they knew* “The death of an old school friend from Leukaemia prompted me to be more proactive.” *Unequivocal* p.428 (La Casta et al., 2019). ***QT** To help a specific patient* “A young girl in our community required a donor.” p.428 *Unequivocal* (La Casta et al., 2019). In relation to donor registration.
		Past personal and family experience Barrier	**QT** ‘“past donation experiences influences decision… “one time I had a vampire that didn't know what they were doing and it messed up my arm for month”’ p.4 *Unequivocal*. (Kaster et al., 2014). Regarding recruitment to register and retention on register.
		Exchange‐related motives Facilitator	***QT** Non‐monetary incentives* “impresses future employers” p. 429 *Unequivocal* (La Casta et al., 2019). In relation to donor registration. **QZ** Expectation of a financial incentive or a small reward were ranked 10th and 12th out of 12 possible motives for joining the register (Bart et al., 2014). **QZ** 45% of registrants reported exchanged‐related motives. Under 40s were more likely to express this motive‐type (Switzer et al., 1997). **QZ** Attrition after the initial stage of registration was lower among those reporting exchange‐related motives (Switzer et al., 1999).
		Knowing about recipient Facilitator	**QT** *Facilitators* “If I was given personal information about the person I was helping, like a picture, or their name, or their age, I would be more willing to donate” p.6 *Unequivocal* (Kaster et al., 2014). **QZ** The motive ‘the identity of the recipient is disclosed’ was ranked 7th out of 12 possible motives by registrants joining the register (Bart et al., 2014).
		Knowing about recipient Barrier	**QZ** Wanting to know something about the potential recipient was associated with reduced willingness (McCullough et al., 1986).
Self‐identity Facilitator	**QT** *Self‐image as giving* “I think that people who donate are different… more compassion” p. 290 *Unequivocal (*Simmons et al., 1993). **QT** *Self‐Image as a Helping Professional* “the fact that I've chosen to be a nurse puts me in the helper‐server role.” *Unequivocal* p. 292 *(*Simmons et al., 1993). **QT** *Self‐Image as Risk‐Taker or Pioneer* “I like doing things other people haven't done. It is ego‐gratification.” p. 291 *Unequivocal* (Simmons et al., 1993). **QZ** Greater self‐identity as a donor was associated with willingness to join registry (Hyde & White, 2013). **QZ** Lower attrition at the initial registration stage was associated with being a volunteer (Switzer et al., 2004). **QZ** Lower attrition at the final stage close to donation with incorporating the HSC donor identity into social roles (Switzer et al., 2013).
	
		Self‐identity Barrier	**QZ** Weaker self‐identity as a donor was associated with attrition at an early stage after registration (Switzer et al., 2004) and the final stage closer to donation (Anthias et al., 2020; Switzer et al., 2004, 2013).
		Empathy Facilitator	**QT** *Personal circumstances* “I was thinking of my children… I really related to the woman, as she might be a mom, she might have children.” p.8 *Unequivocal* (Billen et al., 2017). **QZ** 18% of registrants cited empathy‐based motives with women being more likely to cite this motive type (Switzer et al., 1997). **QZ** ‘Solidarity with fellow humans’ was ranked as the second most important motivation for enrollment (Bart et al., 2014). **QZ** Willingness was associated with high trait empathy (Lee‐Won et al., 2016). **QZ** Attrition after the initial stage of registration was higher among those reporting empathy‐related motives for joining **(**Switzer et al., 1999).
Work & family concerns Barrier	**QT** Absenteeism from work posed another concern “she needed more information about the length of leave required before committing herself to donate and she went on to elaborate on how this had created an embarrassing problem for her at work”’. *Unequivocal* p.35 (Holroyd & Molassiotis, 2000). **QZ** Attrition was associated with having more ‘work & family concerns’ at multiple points of the donation process. Concerns at the initial stage after registration (Switzer et al., 1999, 2004) and at a late stage of the donation process (Anthias et al., 2020; Switzer et al., 2013). Work and family concerns included missing work, ability to care for family, missing family activities, payment for procedure, and travel to and from the donation centre. ***QT**. Bad fortune* “One of my relatives said someone who donated his bone marrow had become mentally retarded. I was thought to be a fool for proceeding with donation” p. 33 *Unequivocal*, (Holroyd & Molassiotis, 2000).

*Note*: Integrated Findings are a merged summary of all the available evidence from studies in the review on facilitators and barriers to donationrelated behaviour (joining the register, donation and attrition from the registry).

Qualitized (QZ)–Quantitative data‐based outcomes and statistical tests were transformed into textual description and narrative interpretation.

Qualitative (QT)–Qualitative data comprised of themes and subthemes taken form qualitative studies included in the systematic review.

Research Question 1: What are the demographic variables influencing unrelated stem cell donation (USCD: joining a stem cell registry and completing stem cell donation), and leaving a stem cell registry (attrition) and do unrelated younger adults, males and individuals from ethnic minority groups differ in relation to facilitators and barriers to USCD and attrition?

##### Integrated finding 1: demographics

Demographic characteristics such as gender, ethnicity, education level and marital status have an influence on USCD behaviour and are presented in Table 1. Predictors of intention to donate were: being married, being a college student or having a higher level of educational attainment. Barriers associated with higher levels of attrition appeared to be older age and unemployment.

##### Age

Ten studies investigated the relationship between age and intention to donate, the relationship between age and intention to donate was inconclusive with contradictory findings reported across seven studies. Five studies showed greater willingness to donate in younger people and two studies showed greater willingness in older people. None of the studies included in the systematic review specifically identified facilitators and barriers that influence USCD unique to one age group. Across all age groups, the preferred method for receiving promotional materials related to USCD was television followed by newspapers (Kwok et al., 2015). Three studies reported older age that was significantly associated with attrition.

##### Gender

Gender was examined in 19 studies, three of which reported a positive association between female gender and intention to donate. The conflicting study which reported a positive association between male gender and willingness to donate had a small sample size and poor methodological quality (Briggs et al., 1986). Regarding attrition, gender was investigated across six studies and female gender was a significant predictor of attrition in one study (Lown et al., 2014). No studies specifically identified facilitators and barriers relating to donation/attrition that were unique to one gender.

##### Ethnicity

Seven studies investigated the difference in facilitators and barriers influencing USCD between ethnic groups. The three most common barriers to donation, afraid of pain, not convenient and health problems listed in Onitilo et al. (2004) are the same across different ethnicities. African American and White people face similar barriers to donation, however in slightly different proportions. African Americans cited the barriers above in the following proportions, afraid of pain 37.4%, not convenient 12% and health problems 13% compared to White people who cited afraid of pain 23%, not convenient 18% and health problems 19.4%. African American and Asian Pacific Islanders reported greater mistrust in the health care system and allocation of Unrelated Stem Cells (USC) compared to White persons, American Indian and Hispanic groups (Switzer et al., 2013).

In a logistic regression of facilitators and barriers influencing attrition, Switzer et al. (2013) found that people with greater ambivalence were at least 15 times more likely to be lost to attrition across all ethnicities. Higher ambivalence towards donation was positively associated with attrition in the African American, White, Hispanic, and Asian Pacific Islander groups, and discouragement was also associated with attrition in the Hispanic population (Switzer et al., 2013). Additionally, higher levels of ambivalence were found in specific ethnic groups: American Indians and Hispanics (Switzer et al., 2013). This may partly explain some of the higher attrition in ethnic groups. Lown et al. (2014) conducted a multivariate logistic regression and found that people from an Asian and Mediterranean ethnicity were 2.64 (99% CI [1.94, 3.60] *p* < .001) and 2.55 (99% CI [1.50,4.34], *p* = .001) times more likely to be lost due to attrition respectively compared to white North Europeans.

##### Education status

Eight studies examined education status, six of which reported higher level of education was significantly associated with increased intention. Educational level was measured slightly differently in each study, the most common method was division into two groups: those with a university degree/attending university compared to those without. Education level was not significantly associated with attrition, this was investigated in four studies.

##### Other demographics

Ten studies examined relationship status, four of which reported being married was a significant facilitator associated with increased intent to donate. All ten studies compared being married to non‐married (single, divorced or widowed). Being a blood donor was examined in ten studies. Five of these were cross sectional or case control which reported a significant positive association with being a blood donor and intention to donate.

Research Question 2: What are they key facilitators and barriers influencing unrelated donors joining a stem cell registry, completing stem cell donation and leaving a stem cell registry (attrition) and how do these relate to the COM‐B model?

##### Integrated finding 2: donation‐related knowledge (COM‐B capability)

Donation‐related knowledge was a facilitator of intention to donate, joining the registry and donation of USCD. Lack of donation‐related knowledge was a barrier to joining the registry and donation and led to attrition from the registry. This integrated finding of ‘knowledge’ was related to the COM‐B component capability. Capability was defined as the individual's psychological and physical capacity to engage in a behaviour, here it is the activity of USCD (Michie et al., 2011). Having the necessary knowledge was part of psychological capability. Overall, 16 studies reported findings about donation‐related knowledge. Of these, 11 were cross‐sectional analytical studies and reported a significant positive association between knowledge and intention to donate. For example, two of the studies; Hyde et al. (2014) *r* = .49, *p* < .001 and Ting et al. (2020) *r* = .41, *p* < .001 both reported a medium effect size, with having knowledge on how to register associated with greater intention to donate. Similarly, five studies reported lack of knowledge as a barrier to intention to donate. A study investigating motives and barriers to joining the stem cell registry, reported that ‘lack of information on donation’ and ‘lack of information on risks’ were the two highest ranked barriers to enrolment (Bart et al., 2014). One study reported low knowledge as a significant predictor of attrition from the register (Switzer et al., 2004). Attrition rates were 2.70 times higher among individuals who were less informed (OR: .70, *p* < .001) at 95% confidence intervals (Switzer et al., 2004).

##### Integrated finding 3: perceived convenience and positive social influence (COM‐B: opportunity)

Perceived convenience was a facilitator of registration. Positive social influences, religious beliefs and the accessibility of positive donation‐related social norms promoted intention to donate, joining the registry and donation of USCD. Physical barriers such as cost, lack of time and inconvenient location were obstacles to registration. This integrated finding is related to the COM‐B component, opportunity. Opportunity was defined as the factors that lie outside the individual that make the behaviour possible or prompt it (Michie et al., 2011). Opportunity was distinguished into physical opportunity afforded by the environment and social afforded by the cultural surroundings that dictates the way that one thinks about things. Facilitators and barriers relating to social opportunity were more frequently studied compared to those related to physical opportunity.

Physical opportunity facilitators and barriers like time, cost, and location of bone marrow drives influenced an individual's intention to donate stem cells. ‘Lack of time’ emerged as a commonly endorsed barrier in the three cross‐sectional surveys (Bart et al., 2014; Narayanan et al., 2016; Norvilitis & Riley, 2001). Hyde et al. (2014) also found that ‘too many constraints on my time’ was associated with lower intention to donate (*r* = −.17, *p* = <.05). Other physical opportunity barriers included cost (Narayanan et al., 2016), and geographic location (Li et al., 2021). Switzer et al. (1999, 2004, 2013) and Anthias et al. (2020) reported that having work or family concerns was positively associated with increased attrition. Work and family concerns included missing work, donation impacting ability to care for family or attend family activities.

Regarding social opportunity, social facilitators and barriers such as peer pressure and media influence were reported to influence an individual's intention to donate stem cells. Influence from friends and family, such as: encouragement, a family member already registered, discussion of donation with family members and attending recruitment drives, were examined in 14 studies. Findings from (10/14) studies suggested that positive influence from friends and family increased an individual's intent to donate. Similarly, discouragement was identified as a barrier to donation by 42.7% of those unwilling to donate by Kwok et al. (2015), although the proportion was just 11.4% in Abdrbo et al., 2017. Three studies (Anthias et al., 2020; Switzer et al., 1999, 2004) found that discouragement from family and friends was associated with increased attrition, and findings from Switzer et al. (1999) suggests positive encouragement can reduce attrition (OR = 0.68, *p* = <.05).

Wider perceived societal norms also appeared to influence donation‐related decisions at the individual level with five studies (Hyde et al., 2014; Hyde & White, 2013; Lee‐Won et al., 2016; Norvilitis & Riley, 2001; Ting et al., 2020) finding that higher perceived subjective norms related to the social desirability of USCD were associated with greater intent to donate. Findings from (4/5) studies reported that media campaigns or public appeals positively influenced individuals' intention to donate.

Intention to donate was associated with religious beliefs. Religious beliefs were cited as important by participants in four of the qualitative studies, they were cited by a small minority of participants in cross‐sectional studies (e.g., 1.2% in Aurelio et al., 2011 and 2.4% in Branach et al., 2018). However, a wider moral imperative to act ‘in accordance with my principles’ was ranked as the fifth most important motivation for registration in Bart et al. (2014). However, in one study, there was an association between religious beliefs in ethnic minorities and objections to donation (Switzer et al., 2013).

Two studies examined the likelihood of attrition among individuals who joined the register with others. Of these, one study reported significantly higher levels of attrition among those who joined the registry with others in their social circle (Switzer et al., 1999). Similarly, the other finding was in the same direction but non‐significant (Switzer et al., 2004). Three studies reported negative influence from friends and family members was a significant predictor of attrition.

##### Integrated finding 4: altruism, sense of duty, fear and health based concerns (COM‐B: motivation)

Altruism was the most commonly cited motive for intention to donate, joining the registry and donation of USCD. Sense of duty, often stemming from religious, cultural or familial values, was associated with intention to donate, joining the register and donation in numerous studies. Important barriers to USCD included health‐based concerns and fears. Motivations to donate ranged from intrinsic to extrinsic‐based. Non intrinsic motivations and ambivalence appear to be associated with later attrition. This integrated finding of ‘altruism, sense of duty, fear and health concerns’ is related to the COM‐B component, motivation. Motivation was defined as all those brain processes that energize and direct behaviour and conscious decision‐making (Michie et al., 2011). This is divided into reflective motivation (involving evaluations and plans) and automatic motivation (involving emotions or impulses from associative learning and/or innate dispositions).

Altruistic motivations part of automatic motivation, such as the desire to save a life or help someone, consistently ranked among the most prevalent motivations for registration or donation across the entire body of identified studies. This is encapsulated in a quotation from one participant in La Casta et al.'s, 2019 paper: *A small act on my part can save someone's life* (p. 428). Proportions of participants citing ‘wanting to help people’ ranged from 37% (Switzer et al., 1997) to 94.4% (Branach et al., 2018) with the motive ‘the prospect of saving lives’ ranked as the most important motivation in a large survey of USCD attitudes among Swiss blood donors (Bart et al., 2014). Those who continued towards USCD at the donor matching stage (e.g. Switzer et al., 1999, 2004, 2013) were more likely to offer generalized altruistic reasons for becoming a donor, whereas those opting out were more likely to have been motivated to join originally by an appeal to help a particular patient.

Another very common motive for donation‐related behaviour was that it stemmed from a sense of duty. This motive, sometimes related to a sense of moral or religious obligation or the belief in ‘giving something back’ (Simmons et al., 1993, p.291), was cited by participants in all six of the included qualitative studies and by 63.6% of participants in Norvilitis and Riley (2001) of registrant attitudes.

Five studies investigated empathy. Of these, (3/5) suggest empathy increased intention to donate and (1/5) found an association between trait empathy and intention to donate (Lee‐Won et al., 2016). However, Switzer et al. (1999) found a weak positive correlation which showed empathy‐based motives for joining the registry, were associated with higher attrition from the registry at a later stage compared to other motives for joining, including religious/social, exchange related or positive feelings which were not associated with such attrition. No conclusion could be given from five studies which investigated the impact of knowing the donor recipient as a facilitator or barrier influencing USCD, due to contradicting results.

Twenty studies investigated and subsequently identified the following barriers to donation: fear of the donation process, side effects, and impact on personal health. Of these, (18/20) were cross‐sectional, randomized control trial or quasi‐experimental studies and repeatedly reported an association between such fears and reduced intent to donate and with attrition (Switzer et al., 1999, 2013). Four studies identified people who had higher ambivalence were significantly more likely to be lost from the register due to attrition.

Research Question 3: Based on the mapping of facilitators and barriers aligned to COM‐B components to the ‘Interventions Functions’ and ‘Policy Categories’ of the Behaviour Change Wheel (BCW), what are the theory and evidence‐based recommendations to improve the design of future interventions to elicit targeted behaviour change in individuals and groups to increase USCD and improve retention on the register?

##### Intervention functions

The integrated findings from Tables 2, 3, 4 identify facilitators and barriers in each component of the COM‐B model. Table 5 shows the combination of the selected intervention functions, policy categories and appropriateness of the policy category based on the APEASE criteria. A comprehensive guide of the multiple steps and tables used to formulate and map the findings to ‘Intervention Functions’ and ‘Policy Categories’ can be viewed in File S6. Intervention functions with examples related to changing behaviour in relation to USCD that were appropriate and satisfied the APEASE criteria were Education, Persuasion, Training, and Modelling. Environmental Restructuring and Enablement partially met the APEASE criteria, further detailed discussion of the benefits and drawbacks of these intervention functions is viewed in Table 5. An example of Enablement would be through the use of Legislation such as ‘soft opt‐in’ legislation which is used for organ donation in some countries.

**TABLE 5:** Identifying appropriate policy categories for an intervention to promote joining the unrelated stem cell donation register, donation and reducing loss from the register.

Intervention function	COM‐B component	Potentially useful policy categories	Does the policy category meet the APEASE criteria (affordability, practicability, effectiveness/cost‐effectiveness, acceptability, side‐effects/safety, equity) in the context of changing behaviours regarding USCD?
Education	Psychological capability Reflective motivation	Communication/Marketing	Yes. Communication to increase knowledge may be beneficial to increase USCD donors as knowledge has been related to positive donation‐related behaviours.
		Guidelines Regulation	Current guidelines regarding USCD and regulation were not identified as barriers to donation. Therefore, new guidelines are unlikely to add further value.
		Legislation	Not appropriate to improve education and knowledge. This is not practical or affordable in relation to APEASE criteria.
		Service Provision	Education of staff on specific barriers (lack of registry contact and lack of information about procedure) will help improve service and reduce attrition.
Persuasion	Automatic motivation Reflective motivation	Communication/Marketing	Yes. Cultivating altruism, and sense of duty in potential donors will help recruitment. These facilitators s were the most commonly cited motivations for joining the register and donation.
		Guidelines Regulation	Current guidelines regarding USCD and regulation were not identified as barriers to donation. Therefore, new guidelines are unlikely to add further value.
		Legislation	Not practicable in this context.
		Service Provision	It is important for potential donors to consent for joining the register or donation without undue influence.
Modelling	Social Opportunity Automatic motivation	Communication/Marketing	Yes. Positive social influences and norms increase positive donation‐related behaviours.
		Service Provision	Not practicable in this context.
Training	Physical capability Psychological capability Physical opportunity Automatic motivation	Guidelines Regulation	As above
		Fiscal provision	Not practicable in this context.
		Legislation	As above.
		Service Provision	Yes. See explanation in Education.
Environment Restructure	Physical Opportunity Social Opportunity Automatic motivation	Guidelines Regulation	As above
		Fiscal measures	As above
		Legislation	Yes. A ‘soft opt in’ approach used in other types of donations mainly organ donation may help recruit and retain more donors. Developing legislation such as ‘opt in’ would require consultation with numerous bodies, patient groups and public consultation which is outside of the scope of the study and not practicable with regards to APEASE.
		Environmental/social planning	Some environmental aspects such as location of recruitment may drives may be changed. This study did not find any specific location to be of benefit to recruitment. Inconvenient location was cited as a barrier to donation, but a ‘convenient location’ was not specifically defined. Other environmental locations such as donor sites and admission to hospital for donation are fixed primarily for safety, logistical and cost effective reasons and are unable to be changed.
Enablement	Psychological capability Social opportunity Automatic motivation	Guidelines Regulation	As above
		Fiscal provision	As above
		Legislation	See ‘soft opt in’ approach detailed in Environment Restructuring.
		Environmental/social planning	See approach detailed in Environment Restructuring
		Service provision	Not practicable in this context. The majority of Service Provision improvements to change behaviour fall under Education/Training or Environmental Restructuring.

*Note*: Policy category selected: Communication/Marketing, and Service Provision are the primary policy categories that can be used to change donation‐related behaviours. Consideration is given to how other policy categories Legislation and Environmental Restructuring can be used to improve positive donation‐related behaviours.

##### Policy categories

Establishment of intervention functions allowed the identification of appropriate policy categories for an intervention. The two main policy categories were Communication and Service Provision. These categories met the APEASE criteria particularly regarding practicality, effectiveness, and acceptability. Legislation and Environmental /Social Restructuring may also be beneficial but will likely require complex input from a variety of stakeholders, therefore, were deemed less appropriate.

## DISCUSSION

This is the first systematic review to investigate facilitators, barriers and demographic variables influencing USCD‐related behaviour. The mapping to the COM‐B model and BCW, allowed the systematic review to provide theory‐ and evidence‐based recommendations to inform future behaviour‐change interventions. Delivery of successful interventions could promote individuals joining the stem cell donation register, completing donation and reducing attrition from the register. The integrated findings from 50 qualitative and quantitative studies included in this study indicate that the most prevalent facilitators that were associated with a positive increase in donation‐related behaviours (intention to donate, willingness to donate, joining the registry and donation) were donation‐related knowledge, social influence, altruism and sense of duty. Key barriers associated with increased attrition from the registry were lack of information and lack of contact from registry staff alongside higher levels of ambivalence. No facilitators or barriers were identified that were unique to specific groups such as young adults, males or ethnic groups including minorities. Furthermore, the use of the COM‐B model identified determinants of USCD behaviour, enabling the specification of potential interventions to overcome barriers and enhance facilitators to increase psychological capability (e.g., donation‐related knowledge), social opportunity (e.g. positive social norms), and reflective motivation (e.g. altruism and sense of duty). Intervention functions found to be potentially effective in bringing about this change were Education, Persuasion, Training, and Modelling. Furthermore, policy categories that may be the most effective, affordable and practical were Communication/Marketing and Service Provision.

Knowledge was the most frequently investigated facilitator and barrier in the identified research literature, examined in (16/50) studies, with strong converging evidence from both qualitative and quantitative studies that knowledge is a key facilitator influencing intention and behaviour. The more one understands the importance of, and the processes involved in USCD, the more likely one is to be willing to register and to donate once matched. Greater knowledge also appears to assuage donation‐related fears. Conversely, the research literature showed that the lack of knowledge was frequently a cited as a barrier to donation and caused attrition from the registry. The importance of social and subjective norms appeared to hold regardless of age and ethnicity. Altruism was the most prevalent motive type offered by participants, and was associated with greater intent to donate, in both ethnic minority and ethnic majority groups. Another prevalent facilitator positively associated with intent to donate was ‘sense of duty’. Participants more often spoke about their sense of duty emanating from a moral value system, sometimes related to religious or cultural background. This review did not identify compelling evidence that there were significant differences in facilitators or barriers influencing USCD behaviour between ethnic groups. Additionally, it did not find any facilitators or barriers s unique to specific gender or age categories.

The facilitators and barriers identified as influencing stem cell donation behaviour were similar to those identified in wider literature research with respect to related stem cell, blood and organ donation behaviour. This potentially means there could be learning to be taken from evidence‐based interventions for promoting blood donation behaviour. Literature on these evidence‐based interventions in blood donation reported mixed results from cash incentives for donation with no overall benefits (Chell et al., 2018; Godin et al., 2012). This was comparable to this systematic review which reported incentives such as cash were not a facilitator to improve behaviours related to donation. A further example of learning could be regarding the delivery of the policy category of communication. The most effective type of communication‐based intervention to increase intention to donate blood was binding communication with high credibility compared to persuasive communication (Fonte et al., 2017). Greater knowledge, social norms and subjective norms, altruism and sense of duty were also facilitators positively associated with related stem cell donation (Garcia et al., 2013; Zomerdijk et al., 2019), blood donation (Carver et al., 2018; Klinkenberg et al., 2019), and blood and organ donation (Ferguson et al., 2019).

The mapping of the COM‐B model has provided two main policy categories that are within the scope of donor registries and recruitment campaigns to elicit behavioural change, which are Communication and Service Provision as shown in Table 5. The facilitators and barriers identified in the results correlated with psychological capability, physical opportunity, social opportunity, and reflective motivation components of the COM‐B model. Intervention functions that were likely to support behaviour change to increase donation, intention to donate and reduce attrition were underpinned by these components Education, Persuasion, Training, Donor Environment Restructuring, Modelling and Enablement. Consideration has also been given to how legislation, regulation and guidelines may influence the targeted behaviours. Communication is a broad policy category, which can be primarily broken into communication to increase knowledge and understanding and communication including media campaigns that focus on Persuasion and Modelling of the targeted behaviour, USCD. Addressing the psychological capability and reflective motivation categories through enhancing knowledge can increase USCD from joining the register to completing donation and reduce attrition. Communication campaigns focused on increasing knowledge could adopt a life‐course approach by providing accessible information tailored to adolescents within the secondary/further education setting. This could help reduce the barriers and poorer uptake by those without a higher education, who are not exposed to recruitment campaigns which are becoming more common across universities. Media campaigns should focus on emotion to elicit behavioural change by focusing on Persuasion interventions, appealing to potential donors' sense of duty and altruistic beliefs. Television followed by newspapers has been shown to be the most popular method of communication to promote USCD, across all age groups between 18 and 60 (Kwok et al., 2015). This may be surprising given the newer forms of digital communication such as social media. Social media has shown mixed results with health information it has been used to support behaviour change (Gough et al., 2017). However, the COVID‐19 pandemic has shown that although disseminating public health messages through social media is possible, misinformation is frequently prevalent accounting for 24.8% of all tweets analysed (Tsao et al., 2021).

The second policy category is Service Provision. Focusing on Service Provision allows multiple different areas of the COM‐B model to be targeted simultaneously. Examples of methods to enable potential donors to engage in USCD behaviour include providing information leaflets on donation process, addressing misconceptions including fear of pain and health concerns and the benefits of donation to increase knowledge and making the process convenient such as good location of recruitment drives. Ensuring relevant follow‐up via email communication from the registry can reduce the amount of attrition from potential donors who have registered but not yet donated. Additionally, there should be a focus on modelling of the targeted behaviour. The use of Modelling as an intervention function encompasses the policy categories of Communication and Service Provision, the intervention function focuses on social opportunity and automatic motivation components of the behavioural change wheel (Michie et al., 2014). An example of this would be the focus of registries on higher profile donors or ambassadors of their cause or include issues such USCD on TV series which could influence social norms positively to increase USCD.

Overall confidence in the health care system and the process of donation were important facilitators highlighted by this study with regards to increasing USCD and preventing registration attrition. Robust guidelines and appropriate regulation to maintain and improve current levels of confidence and to avoid attrition from the registry are currently in place. This has been achieved successfully by the World Marrow Donor Association (WMDA) and are frequently updated and re‐evaluated in line with changing international, legal and regulatory requirements (Shaw et al., 2010; WMDA, 2020). Demographic variables which were researched, could not identify any facilitators or barriers to donation behaviour for individual subgroups such as age, gender and ethnicity. Commonly the same barriers to donation were identified across ethnicities, suggesting that eliminating barriers will be beneficial across the population. Similarly, no demographic differences regarding ethnicity were identified as in a systematic review on facilitators and barriers influencing blood and organ donation (Ferguson et al., 2019). This allows the evidence and theory‐based COM‐B suggestions to be eligible and appropriate to shape behavioural change in relation to USCD for all individuals and subgroups.

### Strengths and limitations

The application of the BCW within the systematic review provided a rich and theoretically‐informed understanding of the causal mechanisms influencing USCD behaviour and how these map on to intervention functions and policy categories to support behaviour change. The BCW is widely used to understand a range of human behaviours, and the COM‐B within this provides a more holistic understanding of causal attributions than other theoretical models. For example, the Theory of Planned Behaviour does not include physical and social opportunity, considering behavioural intention to be determined by attitudes towards behaviour and subjective norms (Rothman, 2004; Weinstein & Rothman, 2005). Social opportunity and physical opportunity have been identified as factors influencing health‐related behaviours such as successful weight gain preventions (Willmott et al., 2021). Another strength of the study was the separation of the term factors into facilitators and barriers. Factors was an ambiguous term used commonly across the literature, separation of this term allows easier mapping and understanding of donation‐related behaviour. Lastly, in deciding on intervention functions and policy categories, the APEASE criteria allowed broad analysis of what may be effective, feasible and implementable.

Although this review had no limiters on context and included studies from countries worldwide, the limiter of studies published in English likely restricted the identification of research across a variety of global healthcare setting published in other languages. One problem with collated global research was that the meaning of ‘ethnic minority’ changed dependent on context and country the research was undertaken in. Additionally, no studies from the African continent met the criteria to be included, therefore, local facilitators or barriers that could affect individuals from this continent that could not be identified. Some nuance may have been lost. An expansive range of terms are used across the literature to measure and gauge donation‐related behaviours. Intention to register, intention to donate, willingness to register and willingness to donate were merged as intention to donate. Due to the similarities in results and to facilitate subsequent mapping of the targeted behaviours to the COM‐B model, intention to donate, joining the register, donation and attrition are referred to as donation‐related behaviours. This amalgamation of terms may lead to a possible loss of distinction of facilitators and barriers that may uniquely affect one of the targeted behaviours above. Although a range of research designs were included, all studies investigating attrition adopted a cross‐sectional approach and participants recounted their motivations and experiences after they had already opted in or out of donation. This may limit our understanding of attrition‐related facilitators or barriers as it could be argued that participants that opted out may engage in post‐hoc justifications regarding their decision such as perceived negative experiences or having been pressurized to join (Chell & Mortimer, 2014). Finally, intervention functions and policy categories would need to be analysed thoroughly for cost effectiveness and affordability in a local context before being applied on a broader scale.

### Future research

Future research should incorporate longitudinal designs, for example, following the same cohort from registration to donation or attrition to avoid the issues already discussed. Although the review included numerous studies, more targeted efforts could significantly increase our overall knowledge of USCD behaviour. The resource developed by Michie et al. (2014) provided direction on how to approach intervention development in a theoretically robust manner, and highlighted the importance of systematic review work to highlight important gaps in the evidence base. A key first step would include identifying specific behaviour change techniques and mode of delivery to change the target behaviour. This would involve prospective studies analysing what techniques work, before applying to a broader population. Overall, this review showed that there was a dearth of qualitative research, which was of significant value in this review in both identifying and clarifying the content of different facilitators or barriers relating to USCD. The systematic review found no facilitators or barriers that uniquely affected specific demographic groups regarding USCD behaviour excluding attrition (i.e. younger age, ethnicity or gender). Further research is needed to identify facilitators or barriers unique to specific subgroups such as younger adults (18–35) because younger donors (18–35) were associated with improved survival in transplant recipients and fewer post‐operative complications compared to older donors (>35) (Fingrut et al., 2017). In the absence of new empirical research, secondary analysis of facilitators and barriers by age group/gender/ethnicity in existing datasets may be beneficial to help develop the evidence base to assist registries in recruiting particular donors.

Future research could build on the intervention functions and policy categories suggested in this systematic review, to develop and evaluate USCD behaviour‐change interventions in local/national contexts. This is the first review to provide evidence‐based rationale for designing future intervention to change behaviour towards USCD. Cost effectiveness of interventions that are successful should be carried out to maximize the use of limited resources available to donor registries.

## CONCLUSIONS AND RECOMMENDATIONS

The findings from this systematic review suggested that donation‐related knowledge, positive social influences, altruism and sense of duty‐based motivations, and positive contact experiences with the USCD registry were key facilitators of intention to donate, joining the registry, and donation of stem cells by unrelated donors. Lack of information and lack of contact from registry staff alongside higher levels of ambivalence increased attrition from registries. Subsequent mapping of these facilitators and barriers to the COM‐B model has increased understanding of behaviours related to USCD including, intention to donate, recruitment to the registry and donation. This systematic review has been able to identify key intervention functions and two main policy categories to increase USCD through behaviour change. The policy categories were Service Provision and Communication: Service Provision through the addressing of barriers such as lack of information and communication from the registry; and Communication through education and increasing the knowledge regarding USCD which could lead to greater USCD in the targeted populations. Addressing the facilitators and barriers associated with USCD outlined in this review can support researchers, clinicians, policymakers, and registries to work towards maximizing future USCD recruitment campaigns.

## AUTHOR CONTRIBUTIONS

**Jessica Forbes:** Data curation; formal analysis; investigation; methodology; validation; visualization; writing – original draft; writing – review and editing. **Paul Rice:** Data curation; formal analysis; investigation; methodology; validation; visualization; writing – review and editing. **Jenny Groarke:** Conceptualization; investigation; methodology; writing – review and editing. **Emma Berry:** Conceptualization; investigation; methodology; writing – review and editing. **Henrietta Graham:** Conceptualization; investigation; methodology; writing – review and editing. **Lisa Graham‐Wisener:** Conceptualization; data curation; investigation; methodology; project administration; supervision; validation; writing – review and editing.

## CONFLICT OF INTEREST STATEMENT

All authors declare no conflict of interest.

## Supporting information


File S1.

File S2.

File S3.

File S4.

File S5.

File S6.

Data S1.

## Data Availability

The data that supports the findings of this study are available in the supplementary material of this article.
