# Supplementary material for: Factors influencing unrelated stem cell donation a mixed‐methods integrated systematic review
Source: Br J Health Psychol. 2024 Oct 24;30(1):e12758. doi: 10.1111/bjhp.12758 (PMC11586825; doi:10.1111/bjhp.12758)
Supplement: Supplementary file 1 — File S1. [file BJHP-30-0-s006.docx]

**S1. Eligibility criteria**

**Inclusion criteria**

**Population:**

- *adults (over the age of 18), of any gender who have:*
- i) not yet registered to become an unrelated stem cell donor
- ii) have registered to become an unrelated stem cell donor (but who have not yet donated)
- iii) who have completed stem cell donation for the first time*,*

**Phenomena of interest:**

- Studies reporting factors influencing stem cell donation
- Studies which relate to stem cell donation via bone marrow or peripheral blood donation

**Context:**

Studies which have been:

- conducted in both low- and high-income countries
- across a range of healthcare and non-healthcare settings

**Type of study:**

- Qualitative (structured/unstructured interviews, open-ended survey questions, and focus groups)
- Quantitative (observational, quasi-experimental and experimental research designs)
- Mixed method
- Empirical studies
- Published from 1980 to present
- Published in English

**Exclusion criteria**

- Not published in English language
- Published before 1980
- Expert reviews, case studies, opinion papers, grey literature, conference proceedings, systematic reviews
- Study population under 18 years of age
- Studies focused on blood cord donation
