# Supplementary material for: Factors influencing unrelated stem cell donation a mixed‐methods integrated systematic review
Source: Br J Health Psychol. 2024 Oct 24;30(1):e12758. doi: 10.1111/bjhp.12758 (PMC11586825; doi:10.1111/bjhp.12758)
Supplement: Supplementary file 2 — File S2. [file BJHP-30-0-s004.docx]

**S2. Search strategy**

**Search undertaken July 2020**

**MEDLINE_Stem Cell**

1. *Tissue Donors/px [Psychology]

2. (Attitude* or Belief* or Deci* or Psycholog* or Opinion* or Perspective* or View* or Behav* or Intention* or Educat* or Aware* or Expertise or Preference* or Familiar* or Motiv* or Incentiv* or Understanding* or Desire* or Facilitat* or Obstacle* or Barrier* or Experience* or Altruism or Perce* or Recruit* or factor* or knowledge).mp. [mp=title, abstract, original title, name of substance word, subject heading word, floating sub-heading word, keyword heading word, organism supplementary concept word, protocol supplementary concept word, rare disease supplementary concept word, unique identifier, synonyms]

3. (Stem Cell* Don* or Marrow Don* or "Non Blood Related Don*" or Hematopoietic Cell* Don* or Stem cell registr* or Bone Marrow registr* or BM Don* or allogen* don* or Stem Cell* Collection or Marrow Collection).mp. [mp=title, abstract, original title, name of substance word, subject heading word, floating sub-heading word, keyword heading word, organism supplementary concept word, protocol supplementary concept word, rare disease supplementary concept word, unique identifier, synonyms]

4. 1 or 3

5. 2 and 4

6. limit 5 to (english language and yr="1980 -Current")

7. (Animals/ or Models, Animal/ or Disease Models, Animal/) not Humans/

8. ((animal or animals or canine* or dog or dogs or feline or hamster* or lamb or lambs or mice or monkey or monkeys or mouse or murine or pig or pigs or piglet* or porcine or primate* or rabbit* or rats or rat or rodent* or sheep* or veterinar*) not (human* or patient*)).ti,kf,jw.

9. 7 or 8

10. 6 not 9

**PsycINFO_Stem Cell**

1. (Stem Cell* Don* or Marrow Don* or "Non Blood Related Don*" or Hematopoietic Cell* Don* or Stem cell registr* or Bone Marrow registr* or BM Don* or allogen* don* or Stem Cell* Collection or Marrow Collection).mp. [mp=title, abstract, heading word, table of contents, key concepts, original title, tests & measures, mesh]

2. limit 1 to (english language and yr="1980 -Current")

**EMBASE_Stem Cell**

1. (Stem Cell* Don* or Marrow Don* or "Non Blood Related Don*" or Hematopoietic Cell* Don* or Stem cell registr* or Bone Marrow registr* or BM Don* or allogen* don* or Stem Cell* Collection or Marrow Collection).mp. [mp=title, abstract, heading word, table of contents, key concepts, original title, tests & measures, mesh]

2. (Attitude* or Belief* or Deci* or Psycholog* or Opinion* or Perspective* or View* or Behav* or Intention* or Educat* or Aware* or Expertise or Preference* or Familiar* or Motiv* or Incentiv* or Understanding* or Desire* or Facilitat* or Obstacle* or Barrier* or Experience* or Altruism or Perce* or Recruit* or factor* or knowledge).mp. [mp=title, abstract, heading word, table of contents, key concepts, original title, tests & measures, mesh]

3. 1 and 2

4. (exp animal/ or exp juvenile animal/ or adult animal/ or animal cell/ or animal tissue/ or nonhuman/ or animal experiment/ or animal model/) not human/

5. 3 not 4

6. limit 5 to (english language and yr="1980 -Current")

**CINAHL_Stem Cell**

1. MM "Hematopoietic Stem Cell Transplantation/ED/NU/PF") OR (MM "Bone Marrow Transplantation/ED/NU/PF") OR (MM "Bone Marrow Transplantation, Allogeneic/ED/NU/PF") OR (MM "Living Donors/PF/ED")
2. Stem Cell* Don* or Marrow Don* or "Non Blood Related Don*" or Hematopoietic Cell* Don* or Stem cell registr* or Bone Marrow registr* or BM Don* or allogen* don* or Stem Cell* Collection or Marrow Collection
3. 1 or 2
4. Attitude* or Belief* or Deci* or Psycholog* or Opinion* or Perspective* or View* or Behav* or Intention* or Educat* or Aware* or Expertise or Preference* or Familiar* or Motiv* or Incentiv* or Understanding* or Desire* or Facilitat* or Obstacle* or Barrier* or Experience* or Altruism or Perce* or Recruit* or factor* or knowledge
5. 3 and 4
6. TI ( (animal or animals or canine* or dog or dogs or feline or hamster* or lamb or lambs or mice or monkey or monkeys or mouse or murine or pig or pigs or piglet* or porcine or primate* or rabbit* or rats or rat or rodent* or sheep* ) NOT (human* or patient*))
7. 5 NOT 6

**Updated search carried out in February 2021**

**MEDLINE_Stem Cell_Feb21**

1. *Tissue Donors/px [Psychology]

2. (Attitude* or Belief* or Deci* or Psycholog* or Opinion* or Perspective* or View* or Behav* or Intention* or Educat* or Aware* or Expertise or Preference* or Familiar* or Motiv* or Incentiv* or Understanding* or Desire* or Facilitat* or Obstacle* or Barrier* or Experience* or Altruism or Perce* or Recruit* or factor* or knowledge).mp. [mp=title, abstract, original title, name of substance word, subject heading word, floating sub-heading word, keyword heading word, organism supplementary concept word, protocol supplementary concept word, rare disease supplementary concept word, unique identifier, synonyms]

3. (Stem Cell* Don* or Marrow Don* or "Non Blood Related Don*" or Hematopoietic Cell* Don* or Stem cell registr* or Bone Marrow registr* or BM Don* or allogen* don* or Stem Cell* Collection or Marrow Collection).mp. [mp=title, abstract, original title, name of substance word, subject heading word, floating sub-heading word, keyword heading word, organism supplementary concept word, protocol supplementary concept word, rare disease supplementary concept word, unique identifier, synonyms]

4. 1 or 3

5. 2 and 4

6. limit 5 to english language

7. limit 6 to dt=20200724-20210225

8. (Animals/ or Models, Animal/ or Disease Models, Animal/) not Humans/

9. ((animal or animals or canine* or dog or dogs or feline or hamster* or lamb or lambs or mice or monkey or monkeys or mouse or murine or pig or pigs or piglet* or porcine or primate* or rabbit* or rats or rat or rodent* or sheep* or veterinar*) not (human* or patient*)).ti,kf,jw.

10. 8 or 9

11. 7 not 10

**Hits = 58**

**PsycINFO_Stem Cell_Feb21**

1. (Stem Cell* Don* or Marrow Don* or "Non Blood Related Don*" or Hematopoietic Cell* Don* or Stem cell registr* or Bone Marrow registr* or BM Don* or allogen* don* or Stem Cell* Collection or Marrow Collection).mp. [mp=title, abstract, heading word, table of contents, key concepts, original title, tests & measures, mesh]

2. limit 1 to english language

3. limit 2 to ch=20200724-2021025

4. limit 2 to up=20200724-2021025

**Hits = 5**

**EMBASE_Stem Cell_Feb21**

1. (Stem Cell* Don* or Marrow Don* or "Non Blood Related Don*" or Hematopoietic Cell* Don* or Stem cell registr* or Bone Marrow registr* or BM Don* or allogen* don* or Stem Cell* Collection or Marrow Collection).mp. [mp=title, abstract, heading word, table of contents, key concepts, original title, tests & measures, mesh]

2. (Attitude* or Belief* or Deci* or Psycholog* or Opinion* or Perspective* or View* or Behav* or Intention* or Educat* or Aware* or Expertise or Preference* or Familiar* or Motiv* or Incentiv* or Understanding* or Desire* or Facilitat* or Obstacle* or Barrier* or Experience* or Altruism or Perce* or Recruit* or factor* or knowledge).mp. [mp=title, abstract, heading word, table of contents, key concepts, original title, tests & measures, mesh]

3. 1 and 2

4. (exp animal/ or exp juvenile animal/ or adult animal/ or animal cell/ or animal tissue/ or nonhuman/ or animal experiment/ or animal model/) not human/

5. 3 not 4

6. limit 5 to english language

7. limit 6 to dd=20200724-20210225

8. limit 6 to rd=20200724-20210225

9. 7 or 8

**Hits = 241**

**CINAHL_Stem Cell_Feb21**

1. MM "Hematopoietic Stem Cell Transplantation/ED/NU/PF") OR (MM "Bone Marrow Transplantation/ED/NU/PF") OR (MM "Bone Marrow Transplantation, Allogeneic/ED/NU/PF") OR (MM "Living Donors/PF/ED")
2. Stem Cell* Don* or Marrow Don* or "Non Blood Related Don*" or Hematopoietic Cell* Don* or Stem cell registr* or Bone Marrow registr* or BM Don* or allogen* don* or Stem Cell* Collection or Marrow Collection
3. S1 or S2
4. Attitude* or Belief* or Deci* or Psycholog* or Opinion* or Perspective* or View* or Behav* or Intention* or Educat* or Aware* or Expertise or Preference* or Familiar* or Motiv* or Incentiv* or Understanding* or Desire* or Facilitat* or Obstacle* or Barrier* or Experience* or Altruism or Perce* or Recruit* or factor* or knowledge
5. S3 and S4
6. TI ((animal or animals or canine* or dog or dogs or feline or hamster* or lamb or lambs or mice or monkey or monkeys or mouse or murine or pig or pigs or piglet* or porcine or primate* or rabbit* or rats or rat or rodent* or sheep* ) NOT (human* or patient*))
7. S5 NOT S6
8. 7 and EM 20200724-

**HITS = 23**
