# Supplementary material for: Factors influencing unrelated stem cell donation a mixed‐methods integrated systematic review
Source: Br J Health Psychol. 2024 Oct 24;30(1):e12758. doi: 10.1111/bjhp.12758 (PMC11586825; doi:10.1111/bjhp.12758)
Supplement: Supplementary file 3 — File S3. [file BJHP-30-0-s005.docx]

| **Quantitative Studies**  **S3. Study characteristics and data extraction table** | | | | | | |
| --- | --- | --- | --- | --- | --- | --- |
| **Study, country** | **Design** | **Participants** | **Phenomena of Interest** | **Variables of interest** | **Outcome**  **Measure(s)** | **Relevant Findings** |
| Abdrbo et al. (2017)  Saudi Arabia | Cross-sectional survey | Adults aged 18-60 from the general public (*N* = 612).  Not Registered (94.8%); Registered (5.2%).  *M* Age = 28.92 (*SD* = 8.63); 74.7% female. | Factors influencing HSC donation. | SOC-D: age; gender; marital status; occupation; health status; income; education.  Donation-related knowledge: 12-item test, ≤ 6 classed as low, ≥ 7 as high knowledge.  Perceived barriers to registration: 9 multiple response items. | Willingness to donate: to family & others;  to family only;  unwilling. | On average, those willing to donate to unrelated others had > knowledge (χ^2^ = 22.89^***^); were > likely to be married (χ^2^ = 7.01^*^); > likely to be college students (χ^2^ = 42.57^***^) & were SIG younger than those only willing to donate to family or those not willing to donate (*d* = .49^***^). NS factors included self-rated health status (*d* = .10); education (χ^2^ = 4.13); salary (χ^2^ = 5.36); & gender (χ^2^ = 4.63).  Concerns of those unwilling to donate included lack of knowledge (52.2%), lack of trust in the system (42.4%), health concerns (41.4%), concerns about after-effects (32.9%) & pain (25%), & religion (4.5%). For those willing to donate but not register, barriers included inadequate knowledge (44.6%), lack of awareness about the registry (42.5%), lack of trust in the system (33.5%), health concerns (35.7%), fear of after-effects (29.3%), fear of pain (21.8%), family (11.1%), fear of death (8.2%), & religious issues (4.3%).  Effect size *d* calculated from *M*s & pooled SD, using an effect size calculator: [www.psychometrica.de/effect_size.html#transform2](http://www.psychometrica.de/effect_size.html#transform2). |
| Anthias et al. (2020)  UK | Cross-sectional survey | AN registrants, preliminarily matched (*N* = 357).  Continued towards donation (*n* = 303): *M* age = 34.16 (*SD* = 10.93); 43% female.  Opted-out (*n* = 54): *M* age = 31.48 (*SD* = 11.41); 43% female.  WH British (WB, *n* = 246): *M* age= 34.02 (*SD* = 11.54); 42% female.  Non-WH British (NWB, *n* = 111): *M* age = 33.15 (SD = 9.82); 46% female; 55% Asian/Asian British; 45% BLK/BLK British. | Reasons for attrition among UK ethnic minorities. | SOC-D: age; sex; employment; relationship status; education; religion; blood donor history.  Culturally-related: Family cohesion (16-item Bardis Familism scale); religious objections to donation (8-item subscale of Organ Donation Attitude Survey); mistrust of the medical system (10-item Health Care System Distrust scale); HSC donation allocation mistrust (6-items adapted from the Organ Donation Attitudes scale).  HRQoL: SF-8 scale physical & mental health composite scores.  Registration related: context for joining (centre type; sample type)*;* registry interaction quality (2 items).  Donation related:  Ambivalence (5 items from Switzer et al., 2004); Self-definition as a donor (2-items from a blood donor scale); medical concerns (5-items); family/work concerns (6 items); Interactions with others (4-items: whether they consulted someone; whether they were encourage/discouraged). | Decision to Continue or Opt-Out.  Ethnicity: White vs Non-White British. | *Differences by donation decision*  *SOC-D:* those who opted-in were > likely to be employed (χ^2^ = 3.95^*^) & to be blood donors (χ^2^ = 11.05^*^). NS factors included: age (*t* =­ 1.65), gender (χ^2^ = .19), marital status (χ^2^ = 3.37), education (χ^2^ = 3.74) & organ-donor status (χ^2^ = .53).  *Culturally-related:* those who opted in were < likely to have religious objections (*t* = –2.62^**^) & to mistrust the fairness of HSC allocation (*t* = –2.19^*^). NS factors included: mistrust of the medical system (*t* = .33) & family cohesion (*t* = .98).  *HRQoL*: those who continued reported > physical (*t* = 2.71^**^) & mental (*t* = 4.35^***^) HRQoL.  *Registry-related*: those who opted in were < likely to have joined at an educational institution (χ^2^ = 8.18^**^). NS factors included joining at a: blood donation centre (χ^2^ = .47); workplace (χ^2^ = 1.83); place of worship (χ^2^ = 2.69); recruitment event (χ^2^ = 2.69); online (χ^2^ = 1.44); appeal for a specific patient (χ^2^ = .67); or an event for a specific ethnic group (χ^2^ = 1.85). The sample (blood/saliva) type provided at registration was also NS (χ^2^ = .98).  *Interactions with the registry*: those who opted in reported having > contact with registry staff (*t* = 2.66^**^) & felt more informed when joining (*t* = 1.92) though the effect was NS. They were also > likely to say that staff explained the donation process (χ^2^ = 3.93^*^). 16% of opt-outs reported that > contact would have changed their decision (χ^2^ = 13.93^***^). Perceived fairness of treatment was NS between ethnic groups (χ^2^ = 1.24).  *Donation-related factors*: those who continued reported < ambivalence (*t* = –13.01^***^); > self-ID as a donor (*t* = 4.33^***^); < medical (*t*=–4.05^***^) & < work/family concerns (*t* = –5.53^***^) & were < likely to have been discouraged from donating (χ^2^ = 4.11^*^). Positive encouragement was NS (χ^2^ = .01).  *Differences by Race/Ethnicity (WB vs NWB)*  *SOC-D*: the NWB group were < likely to be in a marital-like relationship (χ^2^ = 6.23^*^) & < likely to be registered as an organ (χ^2^ = 23.48^***^) or blood donor (χ^2^ = 14.28^***^). NS factors included: age (*t* = –1.61), gender (χ^2^ = .69), employment (χ^2^ = .10) & education (χ^2^ = 3.82).  *Cultural* factors: the NWB group reported > family cohesion (*t* = 4.40^***^) & > religious-based objections (*t* = 2.02^*^), medical system mistrust (*t* = 3.12^**^) & mistrust of HSC donation allocation (*t* = 6.44^***^).  *HRQoL:* perceived physical (*t* = 1.20) & mental health (*t* = –.37) were NS.  *Registry factors*: the NWB group were > likely to have joined at a recruitment event for an ethnic group (χ^2^ = 17.55^***^) The relationship between other recruitment types & ethnicity was NS: blood donation centre (χ^2^ = .00); workplace (χ^2^ = .02); place of worship (χ^2^ = 2.29); recruitment event (χ^2^ = 1.73); educational setting (χ^2^ = .37); online (χ^2^ = 2.81); appeal for a specific patient (χ^2^ = 1.77). The sample type provided was NS (χ^2^ = 2.93).  *Interactions with the registry:* the NWB group were < likely to remember joining (χ^2^ = 3.99^*^) & > likely to believe that ethnicity affected how they were treated (χ^2^ = 10.13^**^) & that > contact with staff would have altered their decision (χ^2^ = 7.99^*^).  *Donation-related:* the NWB had < ambivalence scores (*t* = –2.32^*^) & > self-ID as a donor (*t* = 4.04^***^). NS factors were: medical (*t* = –.93), work & family concerns (*t* = 1.91), & encouragement (χ^2^ = 1.93) or discouragement by others (χ^2^ = .29). |
| Aurelio et al. (2011)  Italy | Cross-sectional survey | New registrants (*N* = 293).  *M* age = 27.7; 51.5% female | Motivations for registration. | SOC-D*:* age; gender; employment; health worker (yes/no); blood donor status.  Fear of donation: multiple choice. | Donor Motives (multiple choice). | Motives reported included: help to patients (67.8%); blood donor (7.8%); nobleness of act (4.6%); personal comfort (3.9%); religious beliefs (1.2%); other (12.3%) (sense of duty (43.9%); previous donation to family /friend (39.0%); family loss (4.9%).  *Fear of donation*: 32.8% expressed fear. Fear was correlated with gender^*^ & was > among females (61% vs 38.5% for males), the working class, & those who felt less informed. Age was NS. |
| Bagcivan et al. (2019)  Turkey | Cross-sectional survey | Not registered (*N* = 361).  Patients (*n* = 192): *M* age = 41 (*SD* = 15.43); 51% female.  Caregivers (*n* = 169): *M* age = 40.48 (*SD* = 13.04); 53.3% female. | Donation-related knowledge & attitudes. | SOC-D: age; gender; education; marital status; family /friends had transplant.  Barriers to donation. | Groups: cancer patient; non-cancer patient; family caregivers. | There was NS difference between patient type for willingness to donate (χ^2^ = 0.02) nor between family caregivers of both patient groups (χ^2^ = 2.33). 72.3% of all participants indicated willingness (No= 7.9% / Neutral = 19.7%). Reasons for unwillingness included: do not know how (33.3%); it is harmful to my health (22.7%); painful (15.1%); other (16.6%). |
| Bagozzi et al. (2001)  Hong Kong  USA | Cross-sectional survey | Non-registered students (*N* = 543):  Hong Kong Chinese (*n* = 190). *M* age = 22.2 (*SD* = 2.00); 64% female.  Chinese Americans (*n* = 107). *M* age= 20.5 (*SD* = 1.69); 44% female.  Black Americans (*n* = 124). *M* age = 20.5 (*SD* = 2.10); 48% female).  White Americans (*n* = 122). *M* age = 19.3 (*SD* = 1.66); 63% female. | Whether attitudes, subjective norms & intentions to donate BM vary depending on thenrolmentip to the donor. | Ethnicity: Hong Kong Chinese (HKC); Chinese American; BLK American; WH American  Relationship to the donor recipient: family member; close relatives; strangers from the same ethnic group; total strangers. | Attitude to donation: 5 items on affective & 5 on evaluative attitudes.  Subjective norms: 4 items.  Intention: to take a screening test & to donate once matched. | For all groups, the gradient for attitude, subjective norm, & intention to donate, were strongest for immediate family, then close relatives, ethnic strangers, & total strangers.  For Hong Kong Chinese, the relationship between attitude & intention was .31 for immediate family, .43 for close relatives, .55 for ethnic strangers & .55 for total strangers. The relationship between subjective norms & intention was .16 for immediate family, .35 for close relatives, .19 for ethnic strangers & .18 for total strangers.  For Chinese Americans, the relationship between attitudes & intention was .24 for immediate family, .34 for close relatives, .54 for ethnic strangers & .58 for total strangers. The relationship between subjective norms & intention was .41 for immediate family, .49 for close relatives, .38 for ethnic strangers & .37 for total strangers.  For black Americans, attitude SIG predicted intention for all targets. Subjective norms predicted intention only for immediate family. The relationship between attitude & intention was .21 for immediate family, .73 for close relatives, .73 for ethnic strangers & .65 for total strangers. The relationship between subjective norms & intention was .23 for immediate family, –.08 for close relatives, –­.01 for ethnic strangers & .09 for total strangers. For white Americans, the correlation between attitude & intention was .48 for immediate family, .47 for close relatives, .24 for ethnic strangers & .22 for total strangers. The relationship between subjective norms & intention was .39 for immediate family, .24 for close relatives, –.41 for ethnic strangers & .29 for total strangers. |
| Balassa et al. (2019)  UK | Cross-sectional review of data on planned donations | Matched donors on the British Bone Marrow Registry (*N* = 2942).  *M* age = 35.8; 35% female. Ethnicity: White (96.8%); Non-White (3.2%) | Attrition at the finally selected donor stage & relationship to blood donation factors. | SOC-D: Age at time of request; gender; ethnicity (white/non-white)  Registry factors: time registered; location of request (UK or international); HSC source (PBSC vs BM)  Blood donation: donor reliability score (1 (best) to 5 (worst) – calculated by attended sessions; type of blood donation (apheresis or whole blood); *M* blood donations /year. | Donor-related cancellation reasons: medically-deferred; personal reasons. | Donor reasons accounted for 46.7% of cancellations (61.7% on medical grounds; 38.3% on personal).  Following regression analysis, poor blood donor reliability score (*OR* = 1.73^***^) & PBSC as the source (*OR* = 2.36^***^) were associated with deferral for any donor-related reason. NS factors were: gender (female vs male) *OR* = 0.91; ethnicity (non-white vs white) *OR* = 0.92; age (< 30 vs > 30) *OR* = 0.74; time on registry *OR* = 1.00; blood donations/yr *OR* = 1.11; blood donation type (apheresis vs whole blood) *OR* = 0.73.  Donor withdrawal for medical reasons was associated with > age (*OR* = 1.36^*)^; & PBSC source (*OR* = 2.22^**^). NS factors were: gender (female vs male) *OR* = 0.79; ethnicity (non-white vs white) *OR* = 0.52; time on registry *OR* = 1.02; blood donations/yr *OR* = 1.08; blood donation type (apheresis vs whole blood): *OR* = .97; blood donor reliability (*OR* = 1.52).  Withdrawal for personal reasons was associated with: age (30-40 vs other ages) *OR* = 1.72^*^; PBSC source vs BM *OR* = 2.43^*^; low blood donor reliability *OR* = 1.94^**^. NS factors included: gender *OR* = 1.13; ethnicity *OR* = 1.50; time on the registry *OR* = 0.96; *M* blood donations *OR* = 1.16; blood donation type OR = 0.41. |
| Bart et al. (2014)  Switzer-land | Cross-sectional survey | General public (*N* = 2631). 77.2% not registered, 22.8% registered. | Motivators for & barriers to joining the stem cell registry. | Motives for enrolling: 10 factors selected based on literature review + 2 free responses.  Obstacles to nrolment: 11 factors selected + 2 free responses. | Rank order of motives & obstacles (determined by Borda count). | Motives to enroll were ranked as follows: the prospect of saving lives (*n* = 7028); solidarity with fellow humans (*n* = 5057); the prospect of increasing a patient’s chances (*n* = 4341); relative/friend needs stem cells (*n* = 4052), accordance with my principles (*n* = 2196), donor centre contacted me (*n* = 1235), identity of the recipient is disclosed (*n* = 1014), accompanied by friend/relative (*n* = 628), financial incentive (*n* = 423), other (*n* = 480), small reward (*n* = 289).  Obstacles were ranked as follows: lack of info on HSC donation (*n* = 7465), lack of info about donation risks (*n* = 4089); afraid of medical procedures (*n* = 1984); lack of time (*n* = 1343); lack of incentives (*n* = 1303); severe health risks (*n* = 597); medical advice/ health check (*n* = 569); too time consuming (*n* = 555); ill health (*n* = 405); no need to register (*n* = 311); other (*n* = 1818). |
| Beatty (1989)  USA | Cross sectional study | Blood donors  (*N* = 27,560).  No interest: *M* age = 34; 51% female.  Interested but not joined: *M* age = 34; 51% female.  Registered: *M* age = 34; 44% female. | The relationship between blood donor history & joining the BM registry. | SOC-D: age; gender; blood donation history | Registry status: no interest; interested but did not consent; consented. | Age, gender, or prior use of blood products were NS predictors of whether a blood donor would join the BM registry.  There was a strong correlation between previous blood donation history & probability of joining (*p* = .001). For example, donors who had given 5 or fewer units had a 5.1% probability of joining the register vs donors who had given 20+ units (*n* = 60) had a 60% probability. |
| Branach et al. (2018)  Poland | Cross-sectional survey | Registered (*N* = 124)  Age: 20-26 = 90.3%; 18-19 = 3.2%; 27-30 = 3.2%; 31-40 = 2.4%; 41-50 = 0.9%.  86.3% female. | Factors influencing the decision to register. | SOC-D: age; gender; education; place of residence; marital status; family status.  Factors influencing decision: knowing someone who had registered or donated; time spent making decision; consulted anyone; registration barriers & fears. | N/A | Factors motivating registration were: to help people (94.4%); to do a good deed (71.8%); sense of duty (35.5%); public campaign (12.9%); family/friend required BMT (8.1%); family/friend influence (8.1%); curiosity (4%); religion (2.4%); no reason (1.6%).  Previous barriers to registration were: lack of possibility (50%); waited to reach adequate age (26.6%); fear of the BM procedure (11.3%); fear of PBSC procedure (8.9%); health reasons (6.5%); fear of possible complications (5.6%).  Fears regarding the collection process included: pain at the injection site (43.5%); fear of general anaesthesia (32.3%); fear of infection (14.5%); fear of flu-like symptoms (1.6%); fear of numbness (1.6%). |
| Briggs et al. (1986)  USA | Quasi-experimental study | Apheresis blood donors, not on BM register (*N* = 261). *M* age = 37.9; 58% female.  12 experimental groups: each received a different questionnaire variant (high-low risk/salience/probability). | Factors that influence willingness to donate BM. | Perceived risk [high/low].  Perceived salience (whether one of a large [high] or small [low] donor pool).  Perceived probability of being called: 1 in 25 [high] vs 1 in 1000 [low].  SOC-D: age; sex; education; marital status; No. of children; prior blood & apheresis donations.  Pre-disposition (5-item scale): personal & family HSC & blood donation experience.  Self-esteem: RSE. | Willingness to commit to donation (4 items). | Perceived risk to the donor had a significant effect on the respondent’s willingness to become a donor (χ^2^ = 31.26^***^): as perceived risk increased, willingness decreased.  The high-risk message was a > deterrent to women (χ^2^ = 21.46^***^) than men (χ^2^= 6.01^*^). Perceived risk also had < effect on single people (χ^2^ = 6.16^*)^ vs those who were married with children (χ^2^ = 18.10^***^). Salience & probability were NS.  The number of prior blood donations (*r* = –.18^**^) & previous experience of apheresis (­­*r* = –.147^*^) were negatively correlated with willingness to donate.  Older people were < willing to volunteer (*r* =­ –.18^**^) & men were > willing to donate than women (*r* = .18^**^). Willingness decreased as participants’ number of children increased (*r* = –.14^*^). Those who had personal or family experience of giving or receiving blood were > willing (*r* = .18^*^). Those with > self-esteem were < willing to donate (*r* = –.15^*^). Education (*r* = .08) & marital status (*r* = .09) were NS. |
| Galanis et al. (2008)  Greece | Cross-sectional survey | *N* = 565.  Athens residents registered on the Greek BMD Registry (*n* = 250): *M* age = 35.6 (± 0.6); 62% female.  Athens residents not registered (*n* = 315): *M* age = 35.2 (*SD* = 0.6); 50.2% female. | Factors that influence the decision to join BM registry. | SOC-D: gender; education; occupation; children.  Donor history [Y/N]: blood or organ donor; relative/friend registered; relative/friend needed BMD; discussed tissue or organ donation with family/friends.  Knowledge: 4 item scale. Attitude: fear of process (Y/N); trust in HCPs (Y/N). | Registration status: registered vs not registered. | The following factors predicted being registered: being female (*OR* = 1.62^**^); having a high school (OR 1.84*) or university education (*OR* = 3.23^***^) as opposed to a primary school education; working as a HCP, scientist or student; having children (*OR* = 1.6^**^), greater knowledge (*OR* = 2^***^); being a regular blood donor (*OR* = 23.4^***^); having a relative or friend already registered (*OR* = 24^***^) or who needed a BM transplant (*OR* = 11.1^***^); discussing tissue & organ donation with family (*OR* = 11.3^***^); trust in health professionals (*OR* = 6.7^***^); received info about BM transplantation (*OR* = 36.8^***^).  Fear of the BM donation process was negatively associated with registration (*OR* = 0.22^***^). |
| Glasgow & Bello (2007)  USA | Cross-sectional survey | Non-registered African Americans (*N* = 220).  *M* age = 32.9, (*SD* = 12.7). 69% female. | Factors influencing donation intentions of African Americans & associated demographic variables. | Theory of planned behaviour: attitudes;  subjective norms;  perceived behavioural control. | Behavioural intent towards BMD. | The attitude ‘Fear or not trusting’ was negatively correlated with intent (^***^) whereas ‘helping others’ was positively correlated (^***^). The subjective norms ‘External influences’ & ‘approval of people’ (^**^) correlated with intent not to donate (^**^). The perceived behaviour control constructs ‘Value of knowledge’ (^***^) & concerns about resources (^***^) correlated with intent to donate. Other factors SIG correlated with intent were: possession of an organ donation card (^***^); being a Methodist (^**^); & being divorced (^**^). |
| Hazzazi et al. (2019)  Saudi Arabia | Cross-sectional survey | Medical students (*N* = 744)  Registered (*n* = 67)  Age: 18-22 = 46.3%; 23-25 = 52.2%; >25 = 1.5%. 61.2% female.  Not registered (*n* = 677).  Age: 18-22 = 63.5%; 23-25 = 34.9%; >25 = 1.6%. 56.3% female. | Donation -related knowledge & attitudes. | SOC-D: age; gender; academic year; education; marital status.  Experience: history of HSC donation; blood donation.  Knowledge: donor eligibility; process-related (6 items)  Attitudes towards registration & donation (5 items).  Concerns: pain; time commitment; long-term side-effects. | Registration status: registered vs not registered. | Being on the registry was SIG associated with age: students aged 23-25 were > likely to be registered (χ^2^ = 7.99^*^). Students studying in the clinical years (χ^2^ = 16.16^***^) & those in year 5 (χ^2^ = 26.46^***)^ were > likely to be registered. Gender (χ^2^ = 0.60) & marital status (χ^2^ = 1.04) were NS.  Those registered were > likely to have a history of: donating HSC to family (χ^2^ = 8.01^**^); & blood donation (χ^2^ = 10.73^***^). Having a family history of blood cancer was NS (χ^2^ = 1.78).  Knowledge: registered students were > likely to correctly answer items about the need to provide a blood sample (χ^2^ = 22.07^***^) & about side-effects (χ^2^ = 8.42^**^). There were NS differences in knowledge related to eligibility (χ^2^ = 0.19), the importance of ethnicity (χ^2^ = 0.66), transplantation procedure (χ^2^ = 1.42) & whether hospitalisation is required (χ^2^ = 2.18).  Among all participants, long-term side effects was the most cited concern (48.7%), followed by time commitment (43.3%) & pain (38.7%). Registered students were < likely to express concern with regard to pain (χ^2^ = 12.10^**^) & side effects (χ^2^ = 6.23) but there was NS difference for concern regarding time commitment (χ^2^ = 5.46).  *NB* χ^2^ values calculated from *n* using an effect size calculator: socscistatistics.com/tests/chisquare2/default2.aspx |
| Hyde & White (2013)  Australia | Longitudinal survey. | Students (aged 18-30) not registered (*N* = 174).  Time 1 (*n* = 174):*M* age = 19.49, (*SD* = 2.23); 76.7% female; 70.7% Caucasian.  Time 2 (*n* = 93):  *M* age = 19.70, (*SD* = 2.15); 79.6% female. | Factors informing intention to join BM registry & the relationship between intention & actual registration behaviour. | Theory of Planned Behaviour predictors:  attitude (4-item scale); subjective norms (2-item scale);  perceived behavioural control (2-item scale).  Moral norm (I feel I ought to register a donor); anticipated regret (2-item scale); self-ID as a donor (2-item scale).  Past blood donation behaviour (yes/no).  SOC-D: gender & age. | Intention to register (2 item scale).  Behaviour (@ 3-months):  joined registry  blood donor screening;  completed a blood donor form; completed ABMDR consent; read the ABMDR brochure; considered registering. | Intention to register was correlated with: attitude (*r* = .39^***)^; subjective norm (*r* = .51^***)^; perceived behaviour control (*r* =.26^***^); moral norm (*r* = .70^***^); anticipated regret *(r* = .60^***^); self-identity (*r* = .66^***^). Gender (*r* = .13), age (*r* = –.09) & past blood donation (*r* = .07) were NS.  Intention to register at Time 1 was correlated SIG with performing one of the outcome measures at time 2 (*r* = .28^**^). |
| Hyde et al. (2014)  Australia | Cross-sectional survey | Students,  not registered (*n* = 150).  Age: 18-40 (*M* = 21.4, *SD* = 4.6). 67% female. | Behavioural, normative, control beliefs, & intentions related to joining a BM registry. | Beliefs: participants rated the likelihood that 9 behavioural beliefs (3 advantages & 6 disadvantages), 8 normative beliefs & 16 control beliefs (10 barriers & six motivators) would occur if they joined the registry.  Self-reported BMD knowledge (1 item).  SOC-D: gender, age. | Intention to join the ABMDR within six months (4-item scale). | Behavioural beliefs negatively correlated with intention to register were: experiencing health risks (*r* = –.20^*^); belief that BM is an invasion of the body (*r* = –.43^***^); pain as a result of donating (*r* = –.21^**^); experiencing pain or side effects as a result of giving blood (*r* = –.21^**^). NS correlations were observed for: saving or improving the lives of others (*r* = .12); being unable to change decision once registered (*r* = –.12); need to undergo surgical procedures for donation (*r* = –.01); knowing someone will donate to me if I need it (*r* = .07).  Normative beliefs that family (*r* = .43^***^), partners (*r* = .45^***^), parents (*r* = .45^***^), friends (*r* = .40^***^), people who need or have received a BM transplant (*r* = .20^*^), medical professionals (*r* = .26^***^) & religious group members (*r* = .16^*^) would approve of the decision to register were SIG associated with > intention.  Significant negative associations between control beliefs & intention included anticipating pain as a result of donating (*r* = –.37^***^); anticipating pain or side effects as a result of giving blood (*r* = –.41^***^); amount of effort required (*r* = –.27^***^); fear of seeing blood (*r* = –.30^***^); having too many constraints on time (*r* = –.17^*^); fear of needles or blood (*r* = –.30^***^). NS beliefs included: “thinking I am ineligible to donate blood or BM” (*r* = –.05); lack of knowledge about the donation process (*r* = .08); & being unaware of the blood collection centre location (*r* = .01).  Control motivator beliefs associated with intention were: hearing about people who have benefitted from a transplant (*r* = .39^***^); knowing someone who needs a transplant (*r* = .25^**^); info about how to register (*r* = .49^***^); knowing more about the donation process & risks (*r* = .39^***^); having friends/family who would join the registry with me (*r* = .20^*^). ‘Personally knowing the transplant recipient’ was NS (*r* = .05). |
| Kwok et al. (2015)  Hong Kong | Cross-sectional survey | General public, aged 18-60 (*N* = 3479).  Not registered (86.4%); registered (13.6%).  50.6% aged 18-32; 31.9% 33-46; 17.4% 47-60. 73.2% female. | Factors associated with donation intention. | SOC-D: age; gender; education; occupation; race.  HSC knowledge: 12-item scale.  Attitudes of those unwilling to donate (8 items). | Intention to donate:  family members only; family & unrelated individuals; unwilling to donate. | Multivariate analysis indicated that those willing to donate to family & unrelated individuals were > likely to: be in the younger two age groups of 18-32 (*OR* = 1.80^***^) & 33-46 (*OR* = 1.61^**^); educated to tertiary level (OR = 1.47^**^); & to have > knowledge (*OR* = 2.55^***^).  Concerns of those unwilling to donate included: health concerns (65.2%); fear of pain (64.4%); after-effects (62.1%); inadequate knowledge (64.1%); lack of trust in the system (35.3%); family concerns (42.7%); death (29.3%); & religion (6.3%). |
| Laver et al. (2001)  USA | Cross sectional survey | African American adults not registered (*N* = 589).  Age: < 20 (7.9%); 20-29 (22.4%); 30-39 (25.7%); 40-49 (21.4%); 50-59 (15.0%); 60-69 (5.9%); <70 (1.7%).  79.4% female. | Barriers to BMD among African American potential donors. | SOC-D: age; gender; ethnicity; education; employment status.  Knowledge: aware of the NMDP; aware that transplantation saves lives; understand donation process; awareness that African Americans are better matches for other African Americans.  Registration site: BM drive; presentation; healthcare facility; health department; work site. | Willingness to become a BM donor. | Those willing to donate were likely to be younger & to have stayed in education longer: 61.8% of those who had received > 12years were willing to donate, vs 43.8% who had received 12 years, & 46.3% who had received < 12 years. People with small children were also > willing compared to those living alone.  Willingness was 1.5 times > among those surveyed at work sites, educational presentations & bone marrow drives (60%) vs at a health care facility or the Health Department (40.4%^***^). Willingness was also associated with knowing that transplantation saves lives (53.5% vs 23.5% of non-willing participants^***^) & that African Americans were the best match for African Americans (56.1% vs 44.2%^*^). Awareness of the NMDP was NS (52.7% vs 49.8%).  A SIG > proportion of those willing to donate selected opportunity (69%) & cost (76.9%) as barriers, rather than fear of pain (36.3%) or inconvenience (30.8%^***^). Respondents who listed cost or lack of opportunity were 1.6 times > likely to express willingness than those selecting other barriers. |
| Lee Won et al. (2018)  USA | RCT | General public, NMDP eligible (aged 18-44) but not registered (*N* = 152). *M* age = 34.01 (*SD* = 6.34); 50% female.  77.0% white; 15.7% African American; 5.3% Asian; 1.3% Hispanic; 0.7% Native American.  Assigned to a low (*n* = 76) or high virality condition (*n* = 76). | The effects of social media virality metrics on perceived social norms & behavioural intent to join a BM registry. | Perceived Threat of Blood Cancers (4 items from Lindsey, 2005).  Trait empathy (2 subscales of the IRI)  Facebook use; Twitter use. | Injunctive norms: 3 items from White et al. 2009.  Intention to register: 3 items from Lindsey (2005) & Stukas et al. (1999). | Twitter use (*r* = .22^**^), trait empathy (*r* = .38^***^) & perceived threat (*r* = .18^*^) were SIG related to perceived injunctive norms. Facebook use was not (*r* = .10).  Facebook (*r* = .17^*^) & Twitter use (*r* = .23^**^), trait empathy (*r* = .40^***^) & perceived injunctive norms (*r* = .68^***^) were SIG correlated with behavioural intent to join the registry. |
| Li et al. (2021)  China | Cross-sectional survey | Donor Program registrants (*N* = 2670).  East Region (*n* = 1074): *M* age = 30.98; 42.2% female.  Middle (*n* = 581): *M* age = 30.33, 43.2% female.  West (*n* = 865): *M* age = 29.02, 46.9% female.  N-Eastern (*n* = 150): *M* age = 32.81; 37.3% female. | Whether characteristics associated with the decision to donate BM differ by region. | SOC-D: age; gender; blood donation history; income.  Donation-related knowledge: 4 items. | Decision to donate: continue/opt-out/ or ambivalent. | Donation decision varied SIG by region: (χ^2^ = 105.10^***^). The eastern region had the highest proportion of people opting to continue towards donation, the north-eastern had the highest rate of opt-out & the lowest proportion of participants reporting that they had previously received publicity or education (χ^2^ = 37.70^***^). |
| Lindsey (2005)  USA | RCT | Students (*N* = 146)  aged 18-31 (*M* = 21.4, *SD* = 1.90); 60% female.  80% White; 10% African American; 6% Asian American; 4% other. | The effect of anticipated guilt on motivation and behavioural intent to donate BM. | Randomly assignment to receive either: a naturalistic (*n* = 48) or a high anticipated guilt message (*n* = 46) promoting BM donation; or to a no message control (*n* = 52). | Anticipated guilt (5 items, Kugler & Jones, 1992).    Behaviour Intent, mean of: 6-item scale; accepted info card about registry; joined mailing list; & willingness to communicate (Morgan & Miller, 2002).  *Post-test (7-10 days)*: actions taken; guilt (Kugler & Jones, 1992). | Each of the path coefficients was substantial & in the predicted direction. The coefficient linking the message & anticipated guilt was .63^*^. Anticipated guilt affected behavioural intent (.84^*^) such that the more guilt respondents anticipated feeling, the greater participants’ intention to donate. The coefficient linking behavioural intent & behaviour was .42^*^, indicating a tendency for those respondents who intended to take action to actually act. |
| Lown et al. (2014)  UK | Retrospective study of AN registry data | AN registrants, typing stage (*N* = 7541):  18-30 years (32.1%); 31-45 (41.9%); 46-60 (26.0%); 37.3% female.  White Northern European (86.0%); African (0.8%); African-Caribbean (2.0%); Asian (3.2%); Eastern European (0.3%); Hispanic (0.1); Jewish (2.0%); Mediterranean (1.1%); Middle Eastern (0.2%); Mixed (0.8%); Oriental (0.1%); Other (0.8%) | Characteristics associated with attrition at CT stage & differences between ethnic groups. | SOC-D: age; gender; ethnicity; prior blood donation.  Length of time on register. | Availability at CT stage. | Donor reasons for attrition included: 19.4% personal reasons; 34.1% medical; 36% due to failure to contact the donor; 7.9% emigrated.  Attrition was higher for: age 31–45 (*OR* = 1.23^***^) & 46–60 years (*OR* = 1.58^***^) when compared to 18–30 years; women (*OR* = 1.32^***^); African (*OR* = 2.78^***^), African-Caribbean (*OR* = 3.07^***^), Asian (*OR* = 2.65^***^), Jewish (*OR* = 1.54^**^), & Mediterranean (*OR* = 2.38^***^) ethnicities when compared to White Northern European; a duration on the register of 6–10 years (*OR* = 1.37^***^), 11–15 years (*OR* = 1.65^***^) & 16+ years (*OR* = 1.40^***^) when compared to < 1–5 years. Attrition was lower for blood donors (*OR* = 0.71^***^). Being Eastern European (*OR* = 0.80); Hispanic (*OR* = 1.25); Middle Eastern (*OR* = 1.87); Mixed race (*OR* = 1.03) or East Asian (*OR* = 0.70) was NS.  There was a SIG effect of ethnicity on the causes of attrition^***^: African & African-Caribbeans had > rates of no contact compared to White Northern Europeans (62.2% & 62.0%, respectively, compared to 35.1% for White Northern Europeans). Asians had > personal deferrals (39.2 vs 19.6%), but < medical deferrals (15.6 vs 33.5%) & similar rates of no contact (39.3 vs 35.1%). Women were SIG > likely to defer for medical reasons (38.9% vs 26.7%^***^) but < likely to be non-contactable (33.7% vs 39.9%). |
| McCullough (1986)  USA | Cross-sectional survey | Blood donors (*N* = 150).  Registered (*n* = 100):  35% aged < 30yrs; 41% 31-40yrs; 24% > 41; 47% female.  Declined to register (*n* = 50): 44% aged < 30yrs; 38% 31-40yrs; 18% > 41; 51% female. | Factors related to the decision to join the BM donor program. | SOC-D: gender; age; marital status; children; education; occupation  Previous donation: blood; apheresis; organ donor.  Religion: influenced decision; factor in life.  Personal experience: know BM recipient or donor  Donation-related: willingness to donate to unrelated vs family/friend;  discussed with another; encouraged /discouraged; ease of decision. | Opt in/out of programme. | Factors associated with being unwilling to participate included: previous donation of whole blood or by apheresis (χ^2^ = 5.07^*^). Organ donation was NS (χ^2^ = 0.06). Participants willing to register were > likely to agree that religion influenced their decision (χ^2^ = 19.70^***^) & were > likely to say that personal experience influenced their decision (χ^2^ = 8.57^**^). Those who declined to participate were > likely to want to know specific details about a stranger before donating (χ^2^ = 49.16^***^) & were > willing to donate to a relative or friend (χ^2^ = 25.19^***^). Gender (χ^2^ = 0.33); age (χ^2^ = 1.33); marital status (χ^2^ = 3.57); education (incomplete data); & occupation (χ^2^ = 2.14) were NS.  Most participants (52% of accepted & 58% of declined) discussed their decision with someone, usually their spouse. The response of that person influenced the decision: 59% of those who agreed were encouraged vs 7% of those who declined to (χ^2^ = 38.61^***^).71% of those who declined were discouraged by their spouse vs 25% of those who agreed (χ^2^ = 38.61^**^).  *NB* χ^2^ values calculated from *n* using an effect size calculator: socscistatistics.com/tests/chisquare2/default2.aspx |
| Mclaren et al. (2012)  Australia | RCT | Adults (aged 18-40) meeting eligibility criteria of ABMDR but not registered (*N* = 141): *M* age = 21.33 (*SD* = 4.57); 66.7% female  67 allocated to the risk condition (15 male/52 female); 74 to no-risk (32 male/42 female). | Risk perceptions related to joining the registry & whether these differ based on gender. | SOC-D: age; gender.  Experimental conditions: received either a questionnaire with donation risk or no risk info.  Blood donation frequency.  Self-reported BMD knowledge. | Perceived risk perceptions (5 items; 7-point scales) of: blood donation; PBSC; BM; registering.  Intention (4 items): to join in the next 6 months; to make an appointment; to complete a consent form; & to donate blood for typing.  Attitudes (7 items).  Subjective norms (3 items).  Self-efficacy (2 items on perceived behavioural control). | For PBSC risk, females perceived a > risk irrespective of condition: there was a significant main effect for gender *F* = 8.79^**^, partial *n^2^* = .06), but not condition, *F* = 1.76, partial *n^2^* = .01, & the interaction was NS, *F* = 0.98, partial *n^2^* = .01. For BM risk, females perceived a > risk irrespective of condition: there was a significant main effect for gender, *F* = 5.35^*^, partial *n^2^* = .04, but not condition, *F* = 0.16, partial *n^2^* = .00, & the interaction was NS, *F* = 0.64, partial *n^2^* =.01. For perceived risk related to joining the BMD register, the condition x gender interaction approached significance, *F* = 3.60, *p* = .060, partial *n^2^* = .03. Men had SIG < registration risk scores in the risk condition vs males in the no-risk condition, *F* = 4.30^*^, partial *n^2^* = .09, but there was a NS effect for females, *F* = 0.45, partial *n^2^* = .01.  For intention, there was a SIG main effect for condition, *F* = 5.24^*^, partial *n^2^* = .04: those in the risk condition had < intention scores vs those in the no-risk condition. The main effect for gender was NS, *F* = 2.56, partial *n^2^* = .02, as was the condition x gender interaction, *F* = 0.01, partial *n^2^* = .04.  For attitude, the condition x gender interaction was SIG, *F* = 5.26^*^, partial *n^2^* = 0.04. Males had < attitude scores in the risk condition vs the no-risk condition, *F* = 8.84^**^, partial *n^2^* = .16, but not between conditions for females, *F* = .02, partial *n^2^* = .00.  For subjective norms, there was a NS effect for condition, F = 1.35, partial *n^2^* =.01, & gender, *F* = 0.22, partial *n^2^* = .00, & the interaction was NS, *F* = 0.13, partial *n^2^* =.00.  For perceived behavioural control, there was a NS main effect for condition, *F* = 1.59, partial *n^2^* = .01, & gender, *F* = 0.37, partial *n^2^* =.00, & the condition x gender interaction was NS, *F* = 1.97, partial *n^2^* =.01. |
| Milaniak (2020)  Poland | Cross- sectional survey | Medical students, not registered (*N* = 311): *M* age = 32.04 (*SD* = 10.94); 87.1% female. | Factors influencing decision making about BMD. | Age.  Empathy (28 item IRI scale).  Altruism (A-N, Sliwak, 2005). | Attitude: 24 items on beliefs & intentions. | A logistic regression analysis indicated that older age (*OR* = 1.05^*^) & higher scores in the personal distress scale (*OR* = 1.13^*^) were associated with unwillingness to donate BM. |
| Mon-aghan (2020)  Canada | Case control study | Canadian registry members, medically eligible for VT (*N =* 1228).  Available at VT (*n* = 662): *M* age at registration = 24.61 (*SD* = 6.63); *M* age at VT =29.96 (*SD* = 8.97); 33.08% female.  Unavailable (*n* = 566): *M* age at registration = 22.83 (*SD* = 6.58); *M* age at VT = 28.04 (*SD* = 9.18); 30.9% female. | Donor factors associated with availability at VT. | SOC-D: age; gender; prior blood donation; resided in province with donor collection centre.  Registration method; length of time between registration & activation. | Availability at VT stage. | Factors associated with availability at VT included:  younger age at registration (*d* = 0.65^***^) & at VT request (*d* = 0.21^***^); method of registration (χ^2^ = 112.36^***^), with those joining at a recruitment event less likely to be available; & prior whole blood donation (χ^2^ = 91.99^***^).  Gender, residing in a province with a donor collection centre & the interval of time between registration & VT were NS. |
| Nara-yanan (2016)  USA | Cross- sectional survey | Medical students (*N* = 99)  Registered (43%): age  21-25 (48% ); 26-30 (41%); >30 (24%). 55% female; 45% WH, 41% other.  Not registered (57%): age 21-25 (52%); 26-30 (59%); >30 (76%). 45% female; 55% WH, 59% other. | Knowledge, motivators & barriers to registering. | SOC-D: gender; age; race.  Blood donor [Y/N]  Knowledge (6 items); prior experience of learning about HSCT (3 items); donation related attitudes (11 items).  Altruistic Personality (20-item scale, Fetzer Institute). | Registry status. | Women were > likely to be registered (χ^2^ = 6.09^*^). Race (χ^2^ = 0.13) & age (χ^2^ = 2.24) were NS. Prior blood donation rates were > in those on the registry (χ^2^ = 5.95^*^). There was NS difference in altruism scores (*t* = 1.00).  Registered students were > likely to give correct answers to questions regarding donor eligibility (χ^2^ = 41.90^***^) & serious side-effects (χ^2^ = 5.69^*^) & had < concerns about potential negative consequences, such as the time commitment^**^, cost^*^, pain^*^, & long-term side effects^*^.  Concerns related to BM donation included: fear of pain (56%)**;** time commitment (52%)**;** long-term side effects (39%) & financial cost (25%).  *NB* χ^2^ values calculated from *n* using an effect size calculator: socscistatistics.com/tests/chisquare2/default2.aspx |
| Norvilitis & Riley (2001)  USA | Cross-sectional survey | College students (*N* = 113): *M* age = 23 (*SD* = 6.2 years). Ethnicity: 87% WH; 4.5% AA; 2.7% AS; 1.8% HIS; 1.8% NA; 2.7% biracial.  Registered (*n* = 66): 77.3% female, 19.7% male, 3% unspecified gender.  Not registered (*n* = 47): 61.7% female. | Personality, knowledge & attitudes of registered & non-registered. | Satisfaction with Life (Diener et al., 1985).  Perceived morality & self-worth (subscales of the Self-Perception Profile for College Students Neeman & Harter, 1986).  Locus of Control (Rotter I-E, 1966).  Social Desirability (Crowne & Marlowe, 1960).  Donation concerns (11 items).  BMD knowledge, attitudes & motivation. | Registry status. | Donor motivations included: wanting to help (69.7%); hope of reciprocity (63.6%); the right thing to do (63.6%); motivated by signs around campus (54.5%); knew someone who has cancer (53.0%); considered donating previously (37.9%); convenience (21.2%); & friend was donating (12.1%).  For those who did not take part, barriers included: being unaware (42.5%); no time (38.3%); not interested (25.5%); medical reasons (25.5%); fear of pain (19.1%); fear of needles (19.1%); unaware of need for BM donors (14.9%); lack of belief in the usefulness of BMD (10.6%); already registered (8.5%); & inconvenient location (8.5%).  Factors associated with participation included: being a frequent blood donor (*t* = 1.94^*^); having friends who participated (χ^2^ = 13.50^**^); knowledge about typing (*t* = 3.75^***^); > social desirability score (vs non-participants who were aware of drive (*t* = 2.20^*^).  Non-participants were > likely to believe that the process was painful (*t* = 4.80^***^). Non-participants who reported they were aware of the drive reported a > fear of needles (*t* = 1.66^**^) & pain (*t* = 2.62^**^). No differences were detected on medical attitudes; fear of medical procedures; & perceived self-competence, locus of control, & satisfaction with life. |
| O’Donnell & Guidry (2020)  Country: N/A (online) | Content analysis of Reddit posts | 665 contributors (25 posts & 1,015 BMD comments). The posts came from: recipients (12%), donors (52%), registrants (12%), potential registrants (4%) & others (friends, family, HCPs, 20%).  Comments: donors (17.3%); registrants (16.4%); potential registrants (9.8%); recipients (7%); others (7%). | How behavioural, normative & efficacy beliefs about BMD are represented on Reddit. | Status of contributor (donor; recipient; registrant; not yet registered; others);  Behavioural, normative & efficacy beliefs. (Ajzen, 1991). | Reddit post engagement scores: median score (upvotes minus downvotes). | Engagement scores were SIG > for commenters who were donors (*Mdn* = 4.0) & for those who had not revealed their relationship to the issue (*Mdn*=4.00), compared to individuals interested in the registry (*Mdn*=2.00). No other differences between relationship types were observed (overall χ^2^ = 24.12^***^).  Comments with positive norms elicited > engagement scores than comments that did not mention norms (*U* = 21440, *Z* = -2.46^*^).  Engagement scores were SIG > for commenters who described donation as easy (*Mdn* = 4.50) compared to commenters who described donation as hard (*Mdn* = 3.00), χ^2^ = 10.35^**^. |
| Onitilo et al. (2004)  USA | Cross-sectional survey | General public (*N* = 829). Not registered.  White (*n* = 421). Age: < 35 = 27.1%; 35-50 = 31.4%; ≥ 50 = 41.6%. 66.9% female.  African American (*n* = 408). Age: < 35 = 27.9%; 35-50 = 38.2%; ≥ 50 = 33.8%. 64.5% female. | Relationship between ethnicity & donation intention. | Non-validated 10-item questionnaire.  SOC-D: age; gender; ethnicity; education  Donation-related knowledge [yes/no]: awareness of NMDP, that BMT saves lives, & the importance of ethnicity to matching.  Reasons for not donating. | Willingness to become a donor [yes/no].  Willingness to be contacted about BM donation. | White people were > aware of the NMDP: χ^2^ = 24.42^***^ & that BMT saves lives: χ^2^ = 9.46^**^. African Americans were > aware that individuals of the same ethnicity provide a better match (χ^2^ = 28.46^***^). There was NS difference for willingness to donate (χ^2^ = 0.42) but WHs were > willing to be contacted about the program (χ^2^ = 8.70^**^).  Those willing to donate were likely to be younger (χ^2^ = 14.64^***^); have had > education (χ^2^ = 44.44^***^), & to have been aware of the NMDP (χ^2^ = 33.50^***^) & that BMT saves lives (χ^2^ = 11.25^***^). Gender was NS (χ^2^ = 0.26).  Among the unwilling, the most common barrier was “afraid of pain” (30%) but a > proportion of African Americans reported this reason (χ^2^ = 13.65^***^). “Health problems” (χ^2^ = 3.99^*^) & “not convenient” (χ^2^ = 4.09^*^) were more commonly cited by white participants. There were NS differences for: no opportunity (χ^2^ = 0.65); cost (χ^2^ = 3.67); religion (χ^2^ = 0.05); lack of trust in the healthcare system (χ^2^ = 0.49); age (χ^2^ = 0.45); would only donate to family (χ^2^ = 0.41); & lack of knowledge (χ^2^ = 1.71). |
| Sarason et al. (1993)  USA | RCT | Blood donors (*N* = 3495). 50.1% female. | The effect of recognising blood donors’ contributions on participation in the BM registry. | Experimental group (*n* = 1197) received brochure, a complimentary letter & a questionnaire.  Brochure only control group (*n* = 1192); no mailing control group (*n* = 1106)  SOC-D: Age; gender. | Registry status. | The percentage joining was SIG > for those who received both the questionnaire & the brochure (12.9%) than for the brochure-only control (6.4%) & the no-mailing control (5.9%) groups, (χ^2^ = *48.4^***^*). The difference in participation rates between the two control groups was NS.  Blood donation history was positively associated with joining the registry. For blood donors who gave a *M* of < 1 unit per year, the questionnaire-&-brochure-treatment group showed x6 the rate of joining the registry compared to the no-mailing control group (7.71% vs 1.15%). Those aged 31-40 joined the registry at a SIG > rate than did either younger or older groups (χ^2^ = 12.7^**^). Gender was NS. |
| Sikora et al. (2014)  Poland | Cross-sectional survey | Students (*N* = 1609): 16.2% registered; 83.8% not registered; *M* age = 21.5 years; 68% female. | Knowledge & attitudes regarding unrelated BMD. | SOC-D: age; gender; university, year & specialization of study | BMD attitude. Registry status (4 items).  Knowledge (6-items). | Registered people had a SIG > level of knowledge about BMD compared to unregistered (36% vs 18%^***^). Among the unregistered, 34% said they had not considered it before & 22% were concerned about health risks. |
| Stroncek et al. (1989)  USA | Cross-sectional structured interviews | BM donors (*N* = 20). Age: 25% 21-30; 40% 30-40; 35% 40-55. 60% female. | Donor motivations. | SOC-D: age, gender; marital status | Motivations for joining BM register. | Donors reported the following motivations: to help another person (80%); for a new experience (10%); because a family member had cancer (10%); because an acquaintance had a BMT (5%); to make life more meaningful (5%). |
| Studts et al. (2010)  USA | RCT | Medical students, not registered (*N* = 102): 54% female;  84% WH, 9% AS, 5% AA, 2% bi-racial. | The efficacy of rational (RA) & emotional appeals (EA) as methods for increasing intention to register with the NMDP. | Appeal type: random assignment to an RA or EA group.  SOC-D: age; gender ethnicity; marital status; religion.  BMD experience: approached by or registered with NMDP; family or friends received BMT; personal or family history of cancer. | Registration decision: [Yes/No]; likelihood of registering [7-point Likert].  Consider talking to family about registering [Yes/No]; likelihood [7-point Likert]. | More of the participants who received the EA indicated that they would register vs those who received the RA (χ^2^ = 13.53^***^). There was NS difference in likelihood of registration (r = .16).    With regard to talking with family members, the group difference was NS on the dichotomous (χ^2^ = 3.44) & likelihood measure (*r* = .16). Married participants were > likely to agree to register (χ^2^ = 4.40^*^) as well as participants who reported a family history of cancer (χ^2^ = 11.05^***^). Gender was NS on the dichotomous measure (χ^2^ = 3.48) but on the likelihood measure females were > likely to agree (*r* = .27^**^). Participants with a family history of cancer reported a > likelihood of registering (*r* = .23^**^). |
| Switzer et al. (1997)  USA | Cross-sectional survey | Participants registered with the NMDP (*N* = 343)  *M* age = 38 (*SD* = 7.6). 44% female. | Donor motives & associated characteristics. | SOC-D: gender; age; religion; marital status; children, education.  Motives: empathy; exchange-related; normative; idealised helping; positive feeling; experienced based. | Ambivalence: 7-item scale (Simmons et al., 1977). | Participants reported the following motives: exchange-related (45%); Idealised helping (37%); normative motives (26%); positive feeling motives (25%); empathy-related (18%); past-experience-based (8%); other motives (9%).  Women were > likely to report empathy (χ^2^ = 5.02^*^) & positive feeling motives (χ^2^ = 3.83^*^); donors under 40 were > likely to cite exchange-based (χ^2^ = 3.66) & idealised helping motives (χ^2^ = 3.67) though the results were NS. No other associations were identified. |
| Switzer et al. (1999)  USA | Cross-sectional survey | Preliminarily matched members of NMDP (*N* = 1014)  Opted to continue typing (*n* = 258):  *M* age = 36; 59% female.  Opted-out (*n* = 756): *M* age = 37;  57% female. | Factors associated with attrition at the DR typing stage. | SOC-D: age; gender; marital status; children; education; ethnicity.  Volunteer history; blood donation; time registered.  Recruitment-related: delayed decision; consulted family/friends or HCPs; encouraged; discouraged.  Recruitment setting: others present; drive for specific patient; ethnicity played a role.  Motives, open-ended responses coded into: empathy; idealized helping; social or religious obligation; exchange-related; expected positive feelings.  Donation concerns. | Opt in/ opt out at DR typing stage. | Demographic characteristics were NS: women (*OR* = 1.09); age (*t* = 1.88); not married (*OR* = 1.02), no children (*OR* = 1.05); college degree (*OR* = 1.27); not employed (*OR* = 1.37); & non-white (*OR* = 0.73).  Blood donors (*OR* = 0.42^***^) were < likely to drop out while those who had been on the registry longer than 4 years were > likely to drop out (*OR* = 6.25^***^). Volunteer history was NS (*OR* = 1.09).  Those who delayed the decision (*OR* = 1.85^***^) or were discouraged from joining (*OR* = 1.94^***^) were > likely to drop out. Those who were encouraged (*OR* = 0.68^*^), consulted relatives (*OR* = 0.78, p = NS) or professionals (*OR* = 0.59^*^) were < likely.  Those who joined with others (*OR* = 1.85^***^), joined at a drive for a specific patient (*OR* = 1.98^***^) or reported that their ethnicity mattered (*OR* = 2.70^***^) were > likely to withdraw.  Empathy motives were linked with > attrition (*OR* = 1.39^**)^ but social or religious (*OR* = 0.62^**^); exchange-related (*OR* = 0.31^***^); or expected positive feelings (*OR* = 0.36^**^) motives were associated with < rates. Idealised helping was NS (*OR* = 0.97).  Medical concerns were associated with attrition: pain (*OR* = 1.48^***^); damage to health (*OR* = 3.28^***^); anaesthesia (*OR* = 1.58^***^); needles (*OR* = 2.22^***^). General concerns associated with attrition included: time off work (*OR* = 2.53^***^); who gets the marrow (*OR* = 4.79^***^); against religion (*OR* = 6.03^***^); & concern that patients’ chances are low (*OR* = 1.84^*^). ‘Concerns that family would worry’ was NS (*OR* = 1.29). |
| Switzer et al. (2003)  USA | Cross-sectional survey | Newly registered to the NMDP (*N* = 426).  52% ≤ 38 yrs; 48% ≥ 39 yrs. 72% female. | Ambivalence among newly recruited potential donors. | Recruitment experience: context (registration centre; joined with others; knew previously registered members); helpfulness of recruitment materials; informativeness of staff; & staff attitudes.  Donation decision: encouraged/discouraged; influenced by religious/ethnic group; intrinsic commitment (2 item-scale).  Perceived knowledge: 3 items & one scale.  Realistic expectations (3 items).  Concerns about donation: medical; work & family concerns. | Ambivalence towards donation: 7-item scale (Switzer et al., 1996). | Previous volunteer work had a significant correlation with ambivalence (*r* = .14^**^). Gender (*r* = .05), age (*r* < .01); race (*r* = .01), marital status (*r* = .02); having children (*r* = .01); education (*r* < .01); & employment (*r* = .02) were NS.  Recruitment context: joining at a drive for a specific patient (*r* = .11^*^) or knowing someone on the registry (*r* = .10^*^) were associated with > ambivalence. Recruitment site (community marrow drive, *r* = .05; blood donation centre, *r* = .07; workplace, *r* = .03; college, *r* = .06; church, *r* = .04; drive for specific ethnic group, *r* = .02), & being with others when registering (*r* = .04) were NS.  Perceptions that recruitment material was < helpful (*r* = .19^***^) & that recruitment staff were < informative (*r* = .19^***^) & < friendly (*r* = .13^**^) were all associated with ambivalence.  There was a > association with ambivalence for those who were discouraged to join (*r* = .17^***^) vs those who were encouraged (*r* = .11^*^). Those who perceived they were encouraged by their culture or religion also reported > ambivalence (*r* = .15^**^). Respondents with < intrinsic commitment scores were more likely to report > ambivalence (*r* = .16^**^).  Those who still had questions about the process (*r* = .17^***^), believed there were risks involved (*r* = .34^***^), felt less informed (*r* = .23^***^) & had unrealistic expectations about donation (*r* = .13^**^) reported > ambivalence.  Those who cited medical (*r* = .42^***^), work or family (*r* = .28^***^), or other concerns (*r* = .14^**^) or believed that serious complications were possible (*r* = .19^***^) or anticipated feeling sad after donating (*r* = .11^*^) were > likely to have > ambivalence. |
| Switzer et al. (2004)  USA | Cross-sectional survey | Potential donors at the DR & CT stages (*N* = 2270).  DR Stage (*n* = 1906).  Opted to continue (*n* = 1727): 53% under 40; 66% female; 19% non-white.  Opted out (*n* = 179): 53% under 40; 64% female; 29% non-white.  CT Stage (*n* = 364)  Opted to continue (*n* = 195): 62% under 40; 60% female; 40% non-white.  Opted out (*n* = 169): 64% under 40; 68% female; 50% non-white. | Factors associated with attrition at the DR & CT stage. | SOC-D: gender; age; marital status; education; ethnicity.  Volunteer-related factors: current volunteer work; blood donation in last year; self-ID as a BMD (5 item scale, adapted from Pilavin et al., 1991); extent to which being a BM donor is incorporated into social roles (8 item scale, from Pilavin et al., 1991).  Psychosocial: emotional wellbeing (Hopkins Symptom checklist); Empathy (RSE); Mastery scale (Pearlin & Schooler, 1978).  Recruitment-related: endorsement of setting; encouragement/discouragement; whether accompanied when joined; joined at a drive for a specific patient; importance of ethnic membership.  Donation-related: ambivalence (Simmons et al., 1987); perceived knowledge (2-item average); realistic expectations (2-item average); medical, work & family concerns.  Donor-centre contact: provision of adequate info (2 items); donor-centre mailings received; remembered registering when contacted; knew why contacted; felt pressured when contacted; provision of info about the patient. | Decision to continue towards donation at DR stage.  Decision to continue at CT stage. | The pattern of bivariate results for DR & CT stage was similar. Among SOC-D characteristics at the CT stage, not being married (*OR* = 0.58^*^) & for both stages, belonging to an ethnic minority group (At DR, *OR* = 1.72^**^; At CT, *OR* = 1.53^*^) were associated with > attrition. The remaining variables were NS: being married (at DR, *OR* = 1.06); being female (at DR, *OR* = 0.93; at CT, *OR* = 1.43); aged under 40 (at DR, *OR* = 1.00; at CT, *OR* = 1.09); college degree (at DR, *OR* = 1.02; at CT, *OR* = 0.75); being employed (at DR, *OR* = 1.14; at CT, *OR* = 0.85).  Among volunteer-related variables, DR-stage respondents who had donated blood in the past year (*OR* = 0.42^***^), & respondents at both stages who had reported that being a BM donor was important to their self-ID (at DR, *OR* = 0.39^***^; at CT, *OR* = 0.39^***^), were < likely to drop out. NS factors included: blood donor in past year (at CT, *OR* = 0.96); volunteer anywhere (at DR, *OR* = 0.79; at CT, *OR* = 1.23); donor-ID incorporated into social role (at DR, *OR* = 0.83; At CT, *OR* = 0.86).  None of the psychosocial variables were associated with attrition at the DR-stage. But at the CT stage, > depression (*OR* = 1.74^**^) & < self-esteem (*OR* = 1.59^*^) were linked to > attrition. NS factors were as follows: anxiety (at DR, *OR* = 1.26; at CT, *OR* = 0.99); depression (at DR, *OR* = 0.97); low self-esteem (at DR, *OR* = 1.00); lower mastery (at DR, *OR* = 1.16; at CT, *OR* = 1.19).  Among recruitment-setting factors, having joined at a community drive was associated with < attrition at the DR-stage (*OR* = 0.68^*^), whereas joining at a college or university was associated with > CT-stage attrition (*OR* = 2.08^*^). Joining at other recruitment settings was NS: blood donation centre (at DR, *OR* = 0.82; at CT, *OR* = 0.75); workplace (at DR, *OR* = 1.18; at CT, *OR* = 0.81); religious institution (at DR, *OR* = 1.37; at CT, *OR* = 1.18); community (at CT, OR = 0.67); university (at DR, *OR* = 1.84).  Respondents who joined because of a specific patient (at DR, OR = 1.52^**^; at CT, *OR* = 1.32, p = NS) or who reported that their own ethnicity was important in their decision (at DR, OR = 1.92^**^; at CT, *OR* = 0.93, p = NS) were > likely to drop out at the DR-stage. Individuals who were either encouraged to join (at DR, *OR* = 1.12, p = NS; at CT, *OR* = 1.64^*^) or discouraged to join by others (at DR, *OR* = 1.23, *p* = NS; at CT, *OR* = 1.79^*^) were > likely to drop out. Whether someone joined the registry with others was NS (at DR, *OR* = 1.36; at CT, *OR* = 1.52).  All the donation-related characteristics were associated with attrition at both stages. Attrition was associated with: > ambivalence (at DR, *OR* = 3.86^***^; at CT, *OR* = 4.18^***^); feeling < informed (at DR, *OR* = 2.03^***^; at CT, *OR* = 2.70^***^); < realistic expectations (at DR, OR = 1.71^**^; at CT, *OR* = 2.44^***^); > medical (at DR, *OR* = 2.47^***^; at CT, *OR* = 2.90^***^); work or family (at DR, *OR* = 1.89^***^; at CT, *OR* = 2.35^***^); & general concerns (at DR, *OR* = 1.59^**^; at CT, *OR* = 1.70^*^).  Registry factors associated with > attrition included: received inadequate info (at DR, *OR* = 2.16^***^; at CT, OR = 3.85^***^); did not receive mailings (at DR, *OR* = 1.72^***^; at CT, *OR* = 1.69^*^); did not remember joining when contacted at DR (at DR, *OR* = 5.00^***^); did not know why they had been contacted (at DR, *OR* = 1.82^***^); felt pressured (at DR, *OR* = 5.46, *p* = NS; at CT, *OR* = 2.86^*^). Receiving info about the donor recipient was associated with < attrition (at DR, *OR* = 0.83, *p* = NS; at CT, *OR* = 0.47^***^). |
| Switzer et al. (2005)  USA | Cross-sectional survey | Completed DR-stage testing & agreed to proceed to donation (*N* = 1679).  White (*n* = 1359): 44% < 40yrs; 65% female.  Black (*n* = 111): 50% < 40yrs; 74% female.  Asian/Pacific Islander (*n* = 83): 64% < 40yrs; 62% female.  Hispanic (*n* = 101): 59% < 40yrs; 69% female.  Native American (*n* = 25): 56% < 40yrs; 76% female. | Donation-related characteristics & ethnicity. | SOC-D: gender; age; marital status; education.  Volunteer-related: self-ID as a donor (5-item scale); incorporation of donor ID into social roles (8-item scale, both Pilavin & Callero, 1991).  Psychosocial: emotional wellbeing (Hopkins Symptom Check list); RSE scale; Mastery (Peralin & Schooler, 1978).  BMD-related: ambivalence (Simmons et al., 1987); perceived knowledge; medical, work & family concerns. | Ethnicity: white; black; Asian/Pacific Islander; Hispanic; Native American. | There were SIG relationships observed between all SOC-D variables & ethnicity apart from gender: women (*r* = .06); < 40yrs (*r* = .11^***^); married (*r* = .19^***^); college degree (*r* = .22^***)^.  There were SIG relationships between all volunteer-related variables & ethnicity: define self as donor (*r* = .11^***^); volunteer-status incorporated into social role (*r* = .15^***^).  There were SIG relationships between all psychosocial variables & ethnicity: anxiety (*r* = .11^***^); depression (*r* = .11^***^); self-esteem (*r* = .11^***^); mastery (*r* = .09^**^).  There were significant relationships between all donation-related variables & ethnicity: ambivalence (*r* = .12^***^); felt informed (*r* = .09^***^); medical concerns (*r* = .15^***^); & work & family concerns (*r* = .11^***^).  Overall, Asian/Pacific Islanders were < likely than other ethnic groups to have internalized a donor self-concept, whereas Native Americans were > likely to have done so. Asian Pacific Islanders reported > general anxiety, ambivalence & medical concerns than other groups, whereas Native Americans reported < anxiety, ambivalence & concerns. This pattern was evident across most non-demographic variables. |
| Switzer et al. (2013)  USA | Cross-sectional structured interviews | NMDP registrants (*N* = 1067).  Opt-in at CT (*n* = 843).  Opt out at CT (*n* = 224).  28.3% White (WH): *M* age = 37.70 (*SD* = 11.52); 52% female.  19.3% African American (AA): *M* age = 38.17 (*SD* = 11.21); 73% female.  24.9% Hispanic/Latino (HIS): *M* age = 38.78 (*SD* = 10.34); 59% female.  20.8% Asian/Pacific Islander (API): *M* age = 34.73 (*SD* = 10.66); 61% female.  6.7% Native American (NA): *M* age = 41.03 (*SD* = 10.28); 61% female. | Factors associated with ethnicity & with the decision to continue at CT stage. | SOC-D: ethnicity; gender; age; marital status; education; employment; income.  Culturally-related:  importance of religion (2-item scale); religious objections to HSC donation (8-items from ODAS, Minniefield et al., 2001); mistrust of the medical system (10-item Health Care System Distrust Scale, Rose et al., 2004); HSC allocation mistrust (6 items from ODAS); family cohesion (16-item Bardis Familism Scale, 1959).  Psychosocial:  anxiety & depression (subscales of the Brief Symptom Inventory); RSE scale; Mastery (Pearlin & Schooler, 1978); Perceived physical & mental health SF-8 subscales (Ware et al., 2001).  Donation related: encouraged /discouraged; ambivalence (7-item scale, Simmons et al., 1977); Self-ID as a donor (2-items from Pilavin & Callero, 1991); extent HSC donor role has been incorporated into social roles (8-item scale, Pilavin & Callero, 1991); medical concerns (4-items); family & work concerns (5-items);  perceived chance of serious complication (1 item); satisfaction with the decision to (or not to) donate (2-item scale). | Opt-in / Opt-out at CT. | There were differences across ethnic groups on all demographic variables except employment status (χ^2^ *=* 6.22). An examination of specific WH vs minority differences indicated that the WH group was < likely to include females than the AA group (χ^2^ = 25.44^***^); was older than the API group (*d* = .58^***^); was > likely to be married than the AA or API groups (χ^2^ = 57.22^***^); was > educated than the HIS group & < educated than the API group; and had > income than the AA, HIS, & AI groups (χ^2^ = 67.70^***^).  Among culturally-related variables, the WH group was < likely to believe that religion played a role in their donation decision than AA & HIS groups (*d* = .85^***^); had fewer religious objections (*d* = .51^***^), & < family cohesion (*d* = .60^***^) than all other groups; < medical mistrust than AA & HIS groups (*d* = .44^***^) and < HSC allocation mistrust than the API group (*d* = .63^***^).  Among psychosocial variables, the WH & AA groups had > self-esteem than the HIS & API groups (*d* = .50^***^) and > mastery than the API group (*d* = .62^***^). Physical (*d* = .26) & mental (*d* = .22) health, anxiety (*d* = .32), & depression scores (*d* = .32) were NS.  Among donation-related characteristics, the WH & API group were > likely to have been encouraged to donate than the AA & HIS groups (χ^2^ = 13.41^**^). The API group was > likely to have been discouraged than all other groups (χ^2^ = 24.67^***^). The WH, AA & API groups had > ambivalence (*d* = .43^**^). The WH group was < likely to self-ID as a donor (*d* = .45^***^) & incorporate a donor ID into their social roles (*d* = .47^***^ than the HIS & AA groups and to have < work & family (*d* = .30^**^), & medical concerns (*d* = .58^***^) than the API group; and < concern about serious risks than the AA group (*d* = .23^*^). Satisfaction with the decision did not differ SIG by ethnicity (*d* = .24).  *Differences by CT decision (opt-in/opt-out)*  Those who opted out were likely to be younger (t = 3.49^***^); unmarried (χ^2^ = 7.38^**^); < educated (χ^2^ = 5.74^*^); unemployed (χ^2^ = 5.12^*^) & have < income (χ^2^ = 11.67^**)^. Gender was NS (χ^2^ = 0.05).  Those who opted out were more likely to have religious objections to donation (*t* = 6.35^***^) & mistrust about the fairness of HSC allocation (*t* = 5.60^***^). The importance of religion in their decision (*t* = 0.92), family cohesion (*t* = 1.05) & medical mistrust (*t* = 1.84) were NS.  Those who opted out had > anxiety (*t* = 4.39^***^) & depression (*t* = 5.53^***^); < lower self-esteem (*t* = 4.94^***^) & mastery (*t* = 4.04^***^) & < physical (*t* = 3.81^***^) & mental health (*t* = 6.30^***^) scores.  Those who opted out: had > ambivalence (*t* = 20.97^***^); & < self-ID (*t* = 7.31^***^) & social ID as a donor (*t* = 3.55^***^); > medical (χ^2^ = 7.91^***^) & work/family (χ^2^ = 7.72^***^) concerns; > belief that donation could result in a serious complication (*t* = 5.24^***^); & were < satisfied with their donation decision (*t* = 34.85^***^).  *Factors associated with CT-stage decision by ethnic group*  For AA participants: > education was associated with a > likelihood of attrition (*OR* = 2.61^**^). NS factors were as follows:   - WH: female sex (*OR* = 0.88); age (*OR* = 0.98); being married (*OR* = 1.30); > education (*OR* = 1.00); being employed (*OR* = 0.60) - AA: female (*OR* = 0.82); age (*OR* = 0.97); being married (*OR* = 1.16); being employed (*OR* = 0.70) - HIS: female (*OR* = 2.07), age (*OR* = 0.99), being married (*OR* = 1.55), > education (*OR* = 1.06); being employed (*OR* = 0.62) - API: female (OR 0.93), age (*OR* = 0.99), being married (*OR* = 1.06), > education (*OR* = 1.82); being employed (*OR* = 1.19).   Attrition was not associated with any of the culturally related variables for any of the ethnic groups.   - WH: religion importance (*OR* = 0.96); religious objections (*OR* = 0.88); family loyalty (*OR* = 1.27); HSC allocation mistrust (*OR* = 0.59). - AA: religion importance (*OR* = 1.18); religious objections (*OR* = 0.50); family loyalty (*OR* = 1.61); HSC allocation mistrust (*OR* = 1.74). - HIS: religion importance (*OR* = 2.88); religious objections (*OR* = 0.47); family loyalty (*OR* = 1.76); HSC allocation mistrust (*OR* = 1.91). - API: religion importance (*OR* = 1.18); religious objections (*OR* = 2.63); family loyalty (*OR* = 1.04); HSC allocation mistrust (*OR* = 1.44).   Psychosocial: for API participants, a < score on the SF-8 mental health scale was associated with > attrition (*OR* = 0.90^**^). NS results were as follows:   - WH: self-esteem (*OR* = 0.52), SF-8 PH (*OR* = 1.01), & SF-8 MH (*OR* = 1.01) - AA: self-esteem (*OR* = 0.48), SF-8 PH (*OR* = 0.92), & SF-8 MH (*OR* = 1.02) - HIS: self-esteem (*OR* = 0.97), SF-8 PH (*OR* = 0.96), & SF-8 MH (*OR* = 0.98) - API: self-esteem (*OR* = 2.86) & SF-8 PH (*OR* = 0.97).   Across all groups, > ambivalence was associated with > attrition: WH (*OR* = 15.03^***^); AA (*OR* = 14.09^***^); HIS (*OR* = 94.56^***^); API (*OR* = 22.16^***^). For the WH group, > self-identification as a donor was associated with < attrition (*OR* = 0.77^**^). For HIS participants, having been discouraged from donating was associated with > attrition (*OR* = 11.80^**^). NS associations were as follows:   - WH: encouraged to donate (*OR* = 2.21); discouraged (*OR* = 2.30); work & family concerns (*OR* = 0.89); chance of serious complication (*OR* = 1.43). - AA: encouraged to donate (*OR* = 1.75); discouraged (*OR* = 2.01); donor self-ID (*OR* = 0.89); work & family concerns (*OR* = 0.98), chance of complication (*OR* = 1.78). - HIS: encouraged to donate (*OR* = 1.14); donor self-ID (*OR* = 1.12); work & family concerns (*OR* = 1.07), risk of complication (*OR* = 1.89). - API: encouraged to donate (*OR* = 1.39); discouraged (*OR* = 1.81); donor self-ID (*OR* = 0.82); work & family concerns (*OR* = 1.23); and risk of serious complication (*OR* = 1.88).   Effect size *d* calculated from *M*s & pooled SD, using an effect size calculator: [www.psychometrica.de/effect_size.html#transform2](http://www.psychometrica.de/effect_size.html#transform2). |
| Ting et al. (2020)  Malaysia | Cross-sectional survey | Blood donors (*N* = 569).  Not registered (95.3%); registered (4.7%). *M* age = 34.6 (± 9.97). 30.8% female.  Ethnicity: Malay (35.3%); Iban (12.8%); Bidayuh (6.3%); Chinese (34.8%); Indian (1.4%); Other (9.3%) | Factors related to HSC donation among blood donors. | SOC-D: age; gender; ethnicity; education  Registered; prior donation; attended promo event.  Knowledge (*M* of 4 items); attitudes (3 items on lawfulness; 5 on trust in the process; 2 on side effects).  Subjective norms (3 items).  Self-efficacy (3 items). | Intention to donate HSC: 3-item scale. | None of the SOC-D characteristics were associated with intention to donate: age (*t* = –1.91); gender (*t* = –1.18); ethnicity (*F* = 1.23); & education (*F* = 1.36).  Knowledge that one must be registered before donating was associated with > intention (*r* = .41^***)^. Knowledge related to the right to withdraw (*r* = .23); the permeance of side-effects (*r* = .06); & relative risk of BM vs PBSC donation (*r* = –.13) was NS.  All 3 attitudinal dimensions were associated with > intention: lawfulness of HSC donation (*r* = .31^***^); the handling of HSC donation (*r* = .28^***^); potential side effects (*r* = .13^**^). Believing that others approved of donation (subjective norm) was associated with > intention (*r* = .32^***^). > perceived self-efficacy positively correlated with intention (*r* = .24^***^). |
| Tus-zynska-Bogucka (2019)  Poland | Cross-sectional survey | General public aged 18-25 (*N*=305): *M* age= 22.36 (SD = 2.01); 37% female.  23.3% registered:  *M* Age = 22.37 (*SD* = 1.91); 63.4% female  76.7% not registered: *M* Age = 22.36 (*SD* = 2.06); 63.2% female. | Predictors of the decision to join the HSC register. | SOC-D: gender; age; education.  Prosocial Behaviour (Moroń, 2012).  Ten Item Personality Index (Gosling et al., 2003).  Self-esteem: RSE.  Family function: Apgar (Smikstein, 1978). | Intention to register. | Demographic factors had a NS relationship to intention to join: gender (χ^2^ = 0.0004); age (*d* =.005); education (χ^2^ = 2.84); family structure (χ^2^ = 0.16); siblings (χ^2^ = 0.55); material standard (χ^2^ = 1.87). Following logistic regression, intention to join the registry was positively associated with satisfactory family affection (OR = 2.27^**^), & conscientiousness (OR = 1.10^*^). Those who reported > philanthropic behaviours were < likely to intend to join (OR = 0.932^*^). NS factors included: self-esteem (OR = 1.01); extraversion (OR = 1.00); agreeableness (OR = 1.03); neuroticism (OR = 0.91); openness (OR = 0.90); family adaptation (OR = 1.26); family partnership (OR = 1.05); family growth (OR = 0.63); family resolve (OR = 0.75); active prosocial behaviours (OR = 0.93); & prosocial support behaviours (OR = 1.05).  *NB* χ^2^ values calculated from *n* using an effect size calculator: socscistatistics.com/tests/chisquare2/default2.aspx. Cohen’s *d* calculated from *M & SDs* using the effect size calculator at campbellcollaboration.org. |
| Varghese & Hem (2015)  India | Cross-sectional survey | Non-registered students (*N* = 81): age 18-20 (85.2%); 21-23 (14.8%). | Knowledge about PBSC donation. | N/A | Knowledge: 20 questions. | 69.1% had inadequate knowledge; 30.9% had moderately adequate knowledge; 0% had ‘adequate’ knowledge. |
| Vascon-cellos et al. (2011).  USA | Cross-sectional survey | Students (*N* = 606): not registered (65.6%); registered (32.7%); didn’t know (1.7%). 69% female. | Donation-related knowledge, attitudes, &  barriers. | Prevalence of beliefs in BMD-related ‘pervasive myths’.  Barriers to joining. | Willingness to join registry. | Those willing to join were > likely to identify myths^***^. Those unwilling were > likely to believe in or be unsure about the myths. Attitudes differed SIG between the groups: fear of pain^***^ was cited by 30% of willing vs 70% of unwilling individuals & 65% were unafraid of complications^***^ vs 28% of unwilling. |
| Vekaria et al. (2020)  USA | Cross-sectional survey | NBMP registrants (*N* = 729).  Newly registered (*n* = 516): *M* age = 21.29 (*SD* = 2.58); 58.9% female, 40.3% male, 0.78% non-binary. 72.7% WH; 8.7% MIX; 6.8% AS; 4.8% BLK/AA; 4.2% HIS; 0.58% NA.  CT (*n* = 213): *M* age = 23.97 (*SD* = 3.02).  Opt-in (*n* = 155): 43.8% female. 81.2% WH; 6.4% MIX; 5.1% AS; 1.9% BLK/AA; 2.6% HIS; 0.64% NA.  Opt-out (*n* = 58): 39.6% female. 59.6% WH; 3.5% MIX; 28% AS; 3.5% BLK/AA; 3.5% HIS. | Construal theory & HSC donation: the role of prospection & ambivalence in the CT stage decision. | SOC-D: age; gender; race; education.  Construals: participants imagined being asked to donate at a future time (1 year for new members & 1 week for matched participants) & then rated items as either positive or negative. No. of items listed, *M* valence of items & heterogeneity (Liberman et al., 2002).  Ambivalence (Switzer et al., 2005, 2013). | Decision of potential matched donors to proceed to CT [yes/no].  Intention to donate of new registrants: likelihood of donating if matched [7-point Likert]. | For the newly registered, more positively valanced construals were associated with > intention to donate (*r* = .272^***^).  Participants who opted in at CT were statistically indistinguishable to newly registered members for construal valence (*t* = –1.53) & construal heterogeneity (*t* = 1.13). But those who opted out of CT had > negative construal valence than new members (*t* = 7.69^***^) & > construal heterogeneity (t = –2.31^*^). Participants who opted in had > positive construal valence (*t* = –5.81^***^), & < construal heterogeneity (*t* = 2.95^**^) than those who opted out.  Negative construal valence was associated with > ambivalence among new members (*r* = –.38^***^) & at CT (*r* = –.49^***^). More construal heterogeneity was positively correlated with > ambivalence among new members (*r* = .30^***^) & at CT (*r* = .19^***^).  Ambivalence was negatively correlated with intention to donate for new members (*r* = –.629^***^) & was > for those who opted out at CT stage (*t* = 12.36^***^). |
| **Qualitative Studies** | | | | | | |
| **Study, country** | **Design** | **Participants** | **Phenomena of Interest** | **Variables of interest** | **Outcome**  **Measure(s)** | **Main Findings** |
| Billen et al. (2017)  UK | Telephone interviews, thematic analysis. | PBSC donors (*N* = 14). 21.4% female.  Ethnicity: 85.7% White Northern European;  7.1% Jewish; 7.1% other White. | Donor characteristics & motivations. | N/A | N/A | Most donors expressed a strong intrinsic motivation to help others or save someone’s life. This was most often expressed in altruistic terms but several donors cited an ‘exchange related motive’. Some donors felt they donated out of a sense of duty while others identified belonging to a religious or cultural group or specific personal circumstances as motivating factors. |
| Dasgupta  (2018)  USA | Qualitative observation, grounded theory. | Recruiters & potential registrants. | Motivations, barriers & facilitators to registration. | N/A | N/A | Concerns about the bone marrow donation process & fear of pain were commonly reported reasons for hesitating to register.  Fear of indefinite commitment caused apprehension among potential recruits. |
| Holroyd & Molas-siotis (2000)  Hong Kong | Semi-structured interviews, thematic analysis. | BM donors (*N* = 27).  Median age 29; 60% female. | Perceptions of Chinese donors including motivations & barriers. | N/A | N/A | Cultural beliefs heavily inform the decision to donate. Parents actively discouraged donation & reinforced false beliefs e.g. that health problems & bad fortune resulted from donation.  Donors reported that they were supported in their decision morally & emotionally by friends of their own generation & this spurred their decision to go through with the donation. Females were stigmatized & discouraged from donating by family & donation was linked to infertility, problems in childbirth & the inability to produce a male child.  A specific media campaign focussed on a little boy diagnosed with cancer was considered influential. Several donors reported that unfavourable media coverage of the safety of hospital procedures had caused them concern. However, several donors felt reassured that the procedure was carried out at a hospital with high hygiene & professional staff.  Work concerns were expressed by some donors though most thought employers were supportive.  Participants found satisfaction in the opportunity to save a life rather than the act of donation itself. Some donors perceived the act of donation in terms of a cost/benefit calculation. Three donors expressed an expectation of a reward such as good fortune. Four donors referred to their Christian beliefs as a motivation. |
| Kaster et al. (2014)  USA | Focus groups, thematic analysis. | Students (*N* = 76)  involved in on-campus recruitment for NMDP (some registered, some not).  Age 18-24. Majority female. | Factors facilitating & preventing registration & donation. | N/A | N/A | Participants highlighted the importance of explaining the donation process to potential registrants & addressing knowledge gaps. Participants felt that the message ‘saving lives & helping people’ was critical in encouraging registration. Identified facilitators included hearing from & about the bone marrow recipient & an easier & faster donation process.  Participants felt that a lack of knowledge was a barrier to recruitment & retention & contributed to fear about the process. Past negative experiences of other forms of donation were also felt to deter people from joining. Other barriers included inconvenience/lack of time, apathy, & monetary cost. |
| La Casta et al. (2019)  UK | Semi-structured interviews. Data coded & motive themes categorised. | Potential donors at VT stage (*N* = 357).  84.9% opted in: *M* age = 34.16; 43.4% female. Ethnicity: White 42.6%, Asian 19.1%, Black 15.2%, Jewish 18.8%, Mediterranean 4.3%.  15.1% opted out: *M* age = 31.48; 42.6% female. Ethnicity: 77.8% White, 5.6% Asian, 7.4% Black, 7.4% Jewish, 1.9% Mediterranean. | Internal & external motivations for joining the registry. | N/A | N/A | Distinct motive categories identified were: “sense of duty”; ‘responded to a public appeal; ‘personal values; ‘desire to save a life’; ‘ethnic background’; ‘to help a specific patient’; ‘easy to donate’; ‘encouraged by friends/family’; ‘why not?’; ‘inspired by illness of someone they knew’; ‘it was convenient’; ‘would want someone to be there for me or my family’; ‘anticipated positive feelings’; ‘to make a difference’; ‘being pressured’; ‘alternative to blood/organ donation’; ‘non-monetary incentive’. |
| Simmons et al. (1993)  USA | Telephone interviews at 3 time intervals: before, shortly after, & 1 year post BMD. | BM donors (*N* = 52). | Donor motivations, self-image & self-esteem. | N/A | N/A | Many participants reported they felt they had special traits. The majority of participants saw themselves as altruistic & believed that their donation was typical for them. However, some donors felt this trait was learned in childhood rather than inherent. Some donors did not see themselves as being ‘altruistic’ but instead were motivated by particular circumstances.  Some donors mentioned moral or normative considerations such as being motivated by a sense of duty. For some participants, donation was seen as an indirect reciprocation for perceived good health or fortune. Some believed their good health obliged them to morally donate.  Many donors saw their religious or social group membership as an influence. Some donors linked their decision to donate to their identity as a helping professional. |
