# Supplementary material for: Factors influencing unrelated stem cell donation a mixed‐methods integrated systematic review
Source: Br J Health Psychol. 2024 Oct 24;30(1):e12758. doi: 10.1111/bjhp.12758 (PMC11586825; doi:10.1111/bjhp.12758)
Supplement: Supplementary file 4 — File S4. [file BJHP-30-0-s003.docx]

**Supplementary File 4**

Number of included studies by country of origin

| Country of origin | Number of studies |
| --- | --- |
| United States of America | 26 |
| United Kingdom | 5 |
| Poland | 4 |
| Australia | 3 |
| China | 3 |
| Saudi Arabia | 2 |
| Canada | 1 |
| Greece | 1 |
| India | 1 |
| Italy | 1 |
| Malaysia | 1 |
| Switzerland | 1 |
| Turkey | 1 |
