# Supplementary material for: Factors influencing unrelated stem cell donation a mixed‐methods integrated systematic review
Source: Br J Health Psychol. 2024 Oct 24;30(1):e12758. doi: 10.1111/bjhp.12758 (PMC11586825; doi:10.1111/bjhp.12758)
Supplement: Supplementary file 5 — File S5. [file BJHP-30-0-s002.docx]

**Supplementary File 5. JBI critical appraisal tables and checklists**

Critical Appraisal Results

*Table 1: JBI Critical Appraisal Results for Analytical Cross-Sectional Studies*

| **Study** | **Methodological item^#^** | | | | | | | |  |
| --- | --- | --- | --- | --- | --- | --- | --- | --- | --- |
|  | **Q1** | **Q2** | **Q3** | **Q4** | **Q5** | **Q6** | **Q7** | **Q8** | **Percentage of criteria met per study (%)** |
| Abdrbo et al., 2017. | N | Y | N | Y | Y | N | Y | N | 50 |
| Bagcivan et al., 2020. | Y | Y | Y | Y | N | N | Y | N | 62.5 |
| Bagozzi et al., 2001. | N | Y | Y | Y | N | N | Y | Y | 62.5 |
| Balassa K et al., 2019. | Y | Y | Y | Y | Y | Y | Y | Y | 100 |
| Bart T et al., 2014. | Y | Y | Y | Y | Y | Y | Y | Y | 100 |
| Beatty et al., 1989. | Y | Y | Y | Y | Y | Y | Y | U | 87.5 |
| Glasgow & Bello, 2007. | Y | Y | Y | Y | Y | Y | Y | Y | 100 |
| Hazzazi et al., 2019. | Y | Y | Y | Y | U | U | Y | Y | 75 |
| Hyde et al., 2014. | U | Y | Y | Y | Y | Y | Y | Y | 87.5 |
| Hyde & White, 2013. | Y | Y | Y | Y | Y | Y | Y | Y | 100 |
| Kwok et al., 2015. | Y | Y | U | Y | Y | Y | Y | Y | 87.5 |
| Laver et al., 2001. | N | Y | N | Y | N | N | U | Y | 37.5 |
| Li et al., 2021. | Y | Y | N | Y | Y | Y | Y | Y | 87.5 |
| McCullough et al., 1986. | Y | Y | Y | Y | N | N | Y | Y | 75 |
| Milaniak et al., 2020. | N | Y | Y | Y | Y | Y | Y | Y | 87.5 |
| Narayanan et al., 2016 | U | Y | N | Y | Y | Y | Y | Y | 75 |
| Onitilo et al., 2004. | Y | Y | U | Y | Y | Y | Y | Y | 87.5 |
| O’Donnell & Guidry, 2020. | Y | Y | N/A | N/A | N/A | N/A | Y | Y | 50 |
| Switzer et al., 2005. | Y | Y | Y | Y | Y | Y | Y | Y | 100 |
| Switzer et al., 2003. | Y | Y | Y | Y | Y | Y | Y | Y | 100 |
| Ting et al., 2020. | Y | Y | Y | Y | Y | Y | Y | Y | 100 |
| Tuszynska-Bogucka W. 2019. | Y | Y | Y | Y | Y | Y | U | Y | 87.5 |
| Vasconcellos et al., 2011. | Y | N | N | Y | N | N | Y | U | 37.5 |
| Vekaria et al., 2020. | Y | Y | Y | Y | Y | Y | Y | Y | 100 |

^#^Methodological items

1. Were the criteria for inclusion in the sample clearly defined?
2. Were the study subjects and the setting described in detail?
3. Was the exposure measured in a valid and reliable way?
4. Were objective, standard criteria used for measurement of the condition?
5. Were confounding factors identified?
6. Were strategies to deal with confounding factors stated?
7. Were the outcomes measured in a valid and reliable way?
8. Was appropriate statistical analysis used?

*Table 2: JBI Critical Appraisal Results for Case Control Studies*

| **Study** | **Methodological item^#^** | | | | | | | | | |  |
| --- | --- | --- | --- | --- | --- | --- | --- | --- | --- | --- | --- |
|  | **Q1** | **Q2** | **Q3** | **Q4** | **Q5** | **Q6** | **Q7** | **Q8** | **Q9** | **Q10** | **Percentage of criteria met per study (%)** |
| Anthias et al., 2020. | Y | Y | Y | Y | Y | Y | N | Y | Y | U | 80 |
| Galanis et al., 2008. | U | U | Y | U | Y | Y | Y | U | Y | Y | 60 |
| Lown et al., 2014. | Y | Y | Y | Y | Y | Y | Y | Y | Y | Y | 100 |
| Monaghan et al., 2021. | Y | Y | Y | Y | Y | Y | Y | Y | Y | Y | 100 |
| Norvilitis & Riley, 2001. | Y | Y | Y | Y | Y | Y | U | Y | Y | N | 80 |
| Switzer et al., 2013. | Y | Y | Y | Y | Y | Y | Y | Y | Y | Y | 100 |
| Switzer et al., 2004. | Y | Y | Y | Y | Y | Y | Y | Y | Y | Y | 100 |
| Switzer et al., 1999. | Y | Y | Y | Y | Y | Y | U | Y | Y | Y | 90 |

^#^Methodological items

1.Were the groups comparable other than the presence of disease in cases or the absence of disease in controls?

2.Were cases and controls matched appropriately?

3.Were the same criteria used for identification of cases and controls?

4.Was exposure measured in a standard, valid and reliable way?

5.Was exposure measured in the same way for cases and controls?

6.Were confounding factors identified?

7.Were strategies to deal with confounding factors stated?

8.Were outcomes assessed in a standard, valid and reliable way for cases and controls?

9.Was the exposure period of interest long enough to be meaningful?

10.Was appropriate statistical analysis used?

*Table 3: JBI Critical Appraisal Results for Cohort Studies*

| **Study** | **Methodological item^#^** | | | | | | | | | | |  |
| --- | --- | --- | --- | --- | --- | --- | --- | --- | --- | --- | --- | --- |
|  | **Q1** | **Q2** | **Q3** | **Q4** | **Q5** | **Q6** | **Q7** | **Q8** | **Q9** | **Q10** | **Q11** | **Percentage of criteria met per study (%)** |
| Switzer et al., 1997. | N/A | N/A | N/A | Y | Y | N/A | Y | Y | N | Y | Y | 54.5 |

^#^Methodological items

1. Were the two groups similar and recruited from the same population?

2. Were the exposures measured similarly to assign people to both exposed and unexposed groups?

3. Was the exposure measured in a valid and reliable way?

4.Were confounding factors identified?

5.Were strategies to deal with confounding factors stated?

6. Were the groups/participants free of the outcome at the start of the study (or at the moment of exposure)?

7. Were the outcomes measured in a valid and reliable way?

8. Was the follow up time reported and sufficient to be long enough for outcomes to occur?

9. Was follow-up complete, and if not, were the reasons to loss to follow-up described and explored?

10. Were strategies to address incomplete follow-up utilized?

11. Was appropriate statistical analysis used?

*Table 4: JBI Critical Appraisal Results for Prevalence Studies*

| **Study** | **Methodological item^#^** | | | | | | | | |  |
| --- | --- | --- | --- | --- | --- | --- | --- | --- | --- | --- |
|  | **Q1** | **Q2** | **Q3** | **Q4** | **Q5** | **Q6** | **Q7** | **Q8** | **Q9** | **Percentage of criteria met per study (%)** |
| Aurelio et al., 2011. | Y | U | Y | Y | Y | U | Y | Y | Y | 77.8 |
| Branach et al., 2018. | Y | U | U | Y | U | U | Y | U | U | 33.3 |
| Sikora et al., 2014. | Y | U | Y | Y | Y | N | Y | Y | Y | 77.7 |
| Stroncek et al., 1989. | U | N | N | Y | Y | U | Y | N | Y | 44.4 |
| Varghese & Hem, 2015. | U | Y | U | N | Y | U | Y | N | U | 33.3 |

^#^Methodological items

1.Was the sample frame appropriate to address the target population?

2.Were study participants sampled in an appropriate way?

3.Was the sample size adequate?

4.Were the study subjects and the setting described in detail?

5.Was the data analysis conducted with sufficient coverage of the identified sample?

6.Were valid methods used for the identification of the condition?

7.Was the condition measured in a standard, reliable way for all participants?

8.Was there appropriate statistical analysis?

9.Was the response rate adequate, and if not, was the low response rate managed appropriately?

*Table 5: JBI Critical Appraisal Results for Qualitative Research*

| **Study** | **Methodological item^#^** | | | | | | | | | |  |
| --- | --- | --- | --- | --- | --- | --- | --- | --- | --- | --- | --- |
|  | **Q1** | **Q2** | **Q3** | **Q4** | **Q5** | **Q6** | **Q7** | **Q8** | **Q9** | **Q10** | **Percentage of criteria met per study (%)** |
| Billen et al., 2017. | Y | Y | Y | Y | Y | N | N | Y | Y | Y | 80 |
| Dasgupta, 2018. | Y | Y | Y | Y | Y | Y | Y | Y | U | Y | 90 |
| Holroyd & Molassiotis, 2000. | Y | Y | Y | Y | Y | N | N | Y | U | Y | 70 |
| Kaster et al., 2014. | Y | Y | Y | Y | Y | N | N | Y | Y | Y | 80 |
| La Casta et al., 2019. | Y | Y | Y | Y | Y | Y | N | Y | Y | Y | 90 |
| Simmons et al., 1993. | U | Y | U | Y | Y | N | N | Y | Y | Y | 60 |

^#^Methodological items

1. Is there congruity between the stated philosophical perspective and the research methodology?
2. Is there congruity between the research methodology and the research question or objectives?
3. Is there congruity between the research methodology and the methods used to collect data?
4. Is there congruity between the research methodology and the representation and analysis of data?
5. Is there congruity between the research methodology and the interpretation of results?
6. Is there a statement locating the researcher culturally or theoretically?
7. Is the influence of the researcher on the research, and vice-versa, addressed?
8. Are participants, and their voices, adequately represented?
9. Is the research ethical according to current criteria or, for recent studies, and is there evidence of ethical approval by an appropriate body?
10. Do the conclusions drawn in the research report flow from the analysis, or interpretation, of the data?

*Table 6: JBI Critical Appraisal Results for Quasi-Experimental Studies*

| **Study** | **Methodological item^#^** | | | | | | | | |  |
| --- | --- | --- | --- | --- | --- | --- | --- | --- | --- | --- |
|  | **Q1** | **Q2** | **Q3** | **Q4** | **Q5** | **Q6** | **Q7** | **Q8** | **Q9** | **Percentage of criteria met per study (%)** |
| Briggs et al., 1986. | Y | U | Y | N | N | Y | Y | Y | U | 55.6 |

^#^Methodological items

1. Is it clear in the study what is the ‘cause' and what is the 'effect' (i.e., there is no confusion about which variable comes first)?
2. Were the participants included in any comparisons similar?
3. Were the participants included in any comparisons receiving similar treatment/care, other than the exposure or intervention of interest?
4. Was there a control group?
5. Were there multiple measurements of the outcome both pre and post the intervention/exposure?
6. Was follow up complete and if not, were differences between groups in terms of their follow up adequately described and analysed?
7. Were the outcomes of participants included in any comparisons measured in the same way?
8. Were outcomes measured in a reliable way?
9. Was appropriate statistical analysis used?

*Table 7: JBI Critical Appraisal Results for Randomized Controlled Trials*

| **Study** | **Methodological item^#^** | | | | | | | | | | | | |  |
| --- | --- | --- | --- | --- | --- | --- | --- | --- | --- | --- | --- | --- | --- | --- |
|  | **Q1** | **Q2** | **Q3** | **Q4** | **Q5** | **Q6** | **Q7** | **Q8** | **Q9** | **Q10** | **Q11** | **Q12** | **Q13** | **Percentage of criteria met per study (%)** |
| Lee-Won et al., 2016. | Y | Y | Y | Y | Y | Y | Y | U | Y | Y | Y | Y | Y | 92.3 |
| Lindsey, 2005. | Y | Y | U | Y | Y | Y | Y | Y | Y | Y | Y | U | Y | 84.6 |
| Mclaren et al., 2012. | Y | Y | N | Y | Y | Y | Y | Y | Y | Y | Y | Y | Y | 92.3 |
| Sarason et al., 1993. | Y | Y | Y | Y | Y | Y | Y | Y | Y | Y | U | U | Y | 84.6 |
| Studts et al., 2010. | Y | U | Y | Y | Y | Y | Y | Y | Y | Y | Y | Y | Y | 92.3 |

^#^Methodological items

1. Was true randomization used for assignment of participants to treatment groups?
2. Was allocation to treatment groups concealed?
3. Were treatment groups similar at the baseline?
4. Were participants blind to treatment assignment?
5. Were those delivering treatment blind to treatment assignment?
6. Were outcomes assessors blind to treatment assignment?
7. Were treatments groups treated identically other than the intervention of interest?
8. Was follow up complete and if not, were differences between groups in terms of their follow up adequately described and analyzed?
9. Were participants analysed in the groups to which they were randomized?
10. Were outcomes measured in the same way for treatment groups?
11. Were outcomes measured in a reliable way?
12. Was appropriate statistical analysis used?
13. Was the trial design appropriate, and any deviations from the standard RCT design (individual randomization, parallel groups) accounted for in the conduct and analysis of the trial?
