# Supplementary material for: Factors influencing unrelated stem cell donation a mixed‐methods integrated systematic review
Source: Br J Health Psychol. 2024 Oct 24;30(1):e12758. doi: 10.1111/bjhp.12758 (PMC11586825; doi:10.1111/bjhp.12758)
Supplement: Supplementary file 6 — File S6. [file BJHP-30-0-s001.docx]

**Supplementary File 6**

1. Based on the mapping of facilitators, barriers, demographics, and COM-B components to the ‘Interventions Functions’ and ‘Policy Categories’ of the behaviour change wheel. What are the theory- and evidence-based recommendations to improve the design of future interventions to elicit targeted behaviour change in individuals and groups to increase USCD and improve retention on the register?

***Table 1. Behavioural analysis and diagnosis.***

| **Target Behaviour:** Increase the amount of people on the USCD register and reduce attrition from the register. | | |
| --- | --- | --- |
| **COM-B components** | **What needs to happen for the target behaviour to occur?** | **Is there a need for change?** |
| ***Physical capability*** | Have the physical skills to sign up to the register. | No change needed as potential donors have these skills. |
| ***Psychological capability*** | Know the correct procedure to sing up to the register. | Change needed as knowledge has been identified as a key facilitator of registration. Improving knowledge before registration may enable USCD registration to increase. |
|  | Know how to create ‘once signed’ rules to prompt donation. | Change needed as registries may face attrition from potential donors ‘once signed’ to register due to lack of knowledge eg: about procedure. This leads to a loss of potential donors. |
| ***Physical opportunity*** | Where possible a convenient process and location is important to facilitate joining the register and USCD. | Change needed, as perceived convenience is a facilitator of registration. Barriers to registration included time, cost, and inconvenient location. |
| ***Social opportunity*** | Social influences, religious beliefs and positive social norms promote intention to donate, joining the register and donation. | Change is not needed as potential donors are influenced positively by social norms and religious beliefs. It may be possible to use the positive effects of social influences, religious beliefs and positive social norms to reach more potential donors. |
| ***Reflective motivation*** | Potential donors most commonly cited altruism as the primary motivation to donation related behaviour. | Change is not needed as many people recognize and cite this belief as a reason for donation. It may be possible to cultivate altruism in potential donors to increase the chances of the targeted donation related behaviour |
|  | Fear and health based concerns are barriers to USCD. | Change needed by addressing and alleviating potential donors fear and health based concerns may increase potential donors joining register and reduce attrition once registered. |
| ***Automatic motivation*** | Have established routines for donation. | Unable to comment. This systematic review primarily focused on first time donation. |
| **Behavioural diagnosis of the relevant COM-B components:** | Psychological capability and physical opportunity need to change in order to elicit the targeted behaviour. Enhancing positive features of social opportunity and reflective motivation may allow further donors to be reached. | |

***Table 2. BCW intervention functions***

| **Intervention function** | **Definition** | ***Example of intervention function in relation to USCD.*** |
| --- | --- | --- |
| **Education** | Increasing knowledge or understanding | *Providing information to promote USCD* |
| **Persuasion** | Using communication to induce positive or stimulate action communication to induce positive or negative feelings or stimulate action | *Using communication methods to cultivate altruism in potential donors, appealing to donors by focusing on sense of duty, religious beliefs and positive social norms to motivate potential donors to join the register.* |
| **Incentivisation** | Creating an expectation of reward | *No literature found incentivization as a facilitator of registration. This may adversely affect donors with altruistic or sense of duty motives.* |
| **Coercion** | Creating an expectation of punishment or cost | *This is an inappropriate method for use. It is vital that donors choose and consent to joining the register.* |
| **Training** | Imparting skills | *Increasing the skills of registry workers to encourage and promote USCD.* |
| **Restriction** | Using rules to reduce the opportunity to engage in the target behaviour (or to increase the target behaviour by reducing the opportunity to engage in competing behaviours) | *This is not applicable to USCD. The target behaviour would not benefit from being restricted.* |
| **Environmental restructuring** | Changing the physical or social context | *Providing prompts at other social activities or health care activities to ask about USCD.* |
| **Modelling** | Providing an example for people to aspire to or imitate | *Using TV drama scenes involving USCD to encourage donation.* |
| **Enablement** | Increasing means/reducing barriers to increase capability (beyond education and training) or opportunity (beyond environmental restructuring) | *The majority of barriers identified can be addressed through education, training and where possible environmental restructuring. An example of enablement would be providing information leaflets in alternative languages.* |

***Table 3. Identifying appropriate intervention functions for an intervention to promote potential donors joining the register and donating and reducing attrition from the register.***

| **Candidate Intervention functions** | **Does the intervention function meet the APEASE criteria (affordability, practicability, effectiveness/cost-effectiveness, acceptability, side-effects/safety, equity) in the context of increasing USCD and reducing attrition?** |
| --- | --- |
| **Education** | Yes. Knowledge regarding USCD increases positive donation related behaviours. |
| **Persuasion** | Yes. Appealing and cultivating altruism, sense of duty and positive social norms regarding donation may influence potential donors to join the register. |
| **Incentivisation** | No. There is no evidence that this would improve donation related behaviours. |
| **Coercion** | Not acceptable to donors. |
| **Training** | Yes. Better training of staff may increase knowledge and decrease attrition from the register. |
| **Restriction** | Not practicable as there are no options to restrict in this context |
| **Environmental restructuring** | Not practicable to restructure the environment on some occasions eg: donor sites will generally be healthcare settings to manage the donation and look after patients. It may be unsafe to choose a more casual setting.  It may be possible to change the sites of donor recruitment drives. |
| **Modelling** | Yes. Positive social norms and social influence have been shown to increase donation related behaviors. |
| **Enablement** | Yes. Although most identified barriers mainly fall into categories that can be addressed by education / training and / or environmental restructuring. |
| **Selected Intervention functions:** | Education, persuasion, training, donor environmental restructuring, modelling and enablement. |

***Table 4. BCW policy categories with examples in relation to USCD.***

| **Policy Category** | **Definition** | **Example** |
| --- | --- | --- |
| **Communication/ marketing** | Using print, electronic, telephonic or broadcast media | *Conducting mass media campaigns to increase knowledge regarding USCD among potential donors.* |
| **Guidelines** | Creating documents that recommend or mandate practice. This includes all changes to service provision | *There are existing protocols and guidelines worldwide that donor registries follow regarding recruitment and retention.* |
| **Fiscal measures** | Using the tax system to reduce or increase the financial cost | *Not related to USCD.* |
| **Regulation** | Establishing rules or principles of behaviour or practice | *Regulation of donor registries already takes place.* |
| **Legislation** | Making or changing laws | *Use of legislation that has been used in other types of organ donation eg: soft opt in. This means that all of the population would be automatically considered a donor instead of having to sign up to the register.* |
| **Environmental/social planning** | Designing and/or controlling the physical or social environment | *Using town planning to ensure that registries and donation centers can be placed in convenient locations where possible.* |
| **Service provision** | Delivering a service | *During the provision and treatment of potential donors. This relates mainly to attrition from the register. Lack of information regarding the procedure and lack of contact from the registry has been shown to increase attrition. Targeting specific barriers identified will help improve service and reduce attrition.* |

***Table 5. Identifying appropriate policy categories for an intervention to promote joining the USCD register, donation and reducing loss from the register.***

| **Intervention function** | **COM-B component** | **Potentially useful policy categories** | **Does the policy category meet the APEASE criteria (affordability, practicability, effectiveness/cost-effectiveness, acceptability, side-effects/safety, equity) in the context of changing behaviours regarding USCD?** |
| --- | --- | --- | --- |
| Education | Psychological capability  Reflective motivation | Communication/marketing | Yes. Communication to increase knowledge may be beneficial to increase USCD donors as knowledge has been related to positive donation related behaviours. |
|  |  | Guidelines  Regulation | Current guidelines regarding USCD and regulation were not identified as barriers to donation. Therefore, new guidelines are unlikely to add further value. |
|  |  | Legislation | Not appropriate to improve communication. This is not practical or affordable in relation to APEASE criteria. |
|  |  | Service provision | Education of staff on specific barriers (lack of registry contact and lack of information about procedure) will help improve service and reduce attrition. |
| Persuasion | Automatic motivation  Reflective motivation | Communication / marketing | Yes. Cultivating altruism, and sense of duty in potential donors will hep recruitment. These factors were the most commonly cited motivations for joining the register and donation. |
|  |  | Guidelines  Regulation | Current guidelines regarding USCD and regulation were not identified as barriers to donation. Therefore, new guidelines is unlikely to add further value. |
|  |  | Legislation | Not practicable in this context. |
|  |  | Service provision | It is important for potential donors to consent for joining the register or donation without undue influence. |
| Modelling | Social Opportunity  Automatic motivation | Communication / marketing | Yes. Positive social influences and norms increase positive donation related behaviours. |
|  |  | Service Provision | Not practicable in this context. |
| Training | Physical capability  Psychological capability    Physical opportunity  Automatic motivation | Guidelines  Regulation | As above |
|  |  | Fiscal provision | Not practicable in this context. |
|  |  | Legislation | As above. |
|  |  | Service Provision | Yes. See explanation in education. |
| Environment Restructure | Physical Opportunity  Social Opportunity  Automatic motivation | Guidelines  Regulation | As above |
|  |  | Fiscal measures | As above |
|  |  | Legislation | Yes. A ‘soft opt in’ approach used in other types of donations mainly organ donation may help recruit and retain more donors. Developing legislation such as ‘opt in’ would require consultations with numerous bodies, patient groups and public consultation which is outside of the scope of the study and not practicable in regards to APEASE. |
|  |  | Environmental / social planning | Some environmental aspects such as location of recruitment may drives may be changed. This study did not find any specific location to be of benefit to recruitment. Inconvenient location was cited as a barrier to donation, but a ‘convenient location’ was not specifically defined.  Other environmental locations such as donor sites and admission to hospital for donation are fixed primarily for safety, logistical and cost effective reasons and are unable to be changed. |
| Enablement | Psychological capability  Social opportunity  Automatic motivation | Guidelines  Regulation | As above |
|  |  | Fiscal provision | As above |
|  |  | Legislation | See ‘soft opt in’ approach detailed in environment restructuring. |
|  |  | Environmental/social planning | See approach detailed in environment restructuring |
|  |  | Service provision | Not practicable in this context. The majority of service provision improvements to change behaviour fall under education / training or environmental restructuring. |
| Policy category selected: Communication/marketing, modelling and service provision are the primary policy categories that can be used to change donation related behaviours. Consideration is given to how other policy categories legislation and environmental restructuring can be used to improve positive donation related behaviours. | | | |

Tables adapted from Michie, S., Atkins, L., & West, R. (2014). The behaviour change wheel. *A guide to designing interventions. 1st ed. Great Britain: Silverback Publishing*, *1003*, 1010.
