# Supplementary material for: Factors influencing unrelated stem cell donation a mixed‐methods integrated systematic review
Source: Br J Health Psychol. 2024 Oct 24;30(1):e12758. doi: 10.1111/bjhp.12758 (PMC11586825; doi:10.1111/bjhp.12758)
Supplement: Supplementary file 7 — Data S1. [file BJHP-30-0-s007.docx]

**Supporting material**

**Contents**

S1. PRISMA reporting checklist……………………………………………………………………………………………… 2-5

S2. Extracted data items………………………………………………………………………………………………………….6

S3. List of included studies………………………………………………………………………………………………………7-13

S4. List of excluded studies including reasons for exclusion…………………………………………………….14-27

| **Section and Topic** | **Item #** | **Checklist item** | **Reported (Yes/No)** |
| --- | --- | --- | --- |
| **TITLE** | | |  |
| Title | 1 | Identify the report as a systematic review. | Yes |
| **BACKGROUND** | | |  |
| Objectives | 2 | Provide an explicit statement of the main objective(s) or question(s) the review addresses. | Yes |
| **METHODS** | | |  |
| Eligibility criteria | 3 | Specify the inclusion and exclusion criteria for the review. | Yes |
| Information sources | 4 | Specify the information sources (e.g. databases, registers) used to identify studies and the date when each was last searched. | Yes |
| Risk of bias | 5 | Specify the methods used to assess risk of bias in the included studies. | Yes |
| Synthesis of results | 6 | Specify the methods used to present and synthesise results. | Yes |
| **RESULTS** | | |  |
| Included studies | 7 | Give the total number of included studies and participants and summarise relevant characteristics of studies. | Yes |
| Synthesis of results | 8 | Present results for main outcomes, preferably indicating the number of included studies and participants for each. If meta-analysis was done, report the summary estimate and confidence/credible interval. If comparing groups, indicate the direction of the effect (i.e. which group is favoured). | Yes |
| **DISCUSSION** | | |  |
| Limitations of evidence | 9 | Provide a brief summary of the limitations of the evidence included in the review (e.g. study risk of bias, inconsistency and imprecision). | Yes |
| Interpretation | 10 | Provide a general interpretation of the results and important implications. | Yes |
| **OTHER** | | |  |
| Funding | 11 | Specify the primary source of funding for the review. | N/A |
| Registration | 12 | Provide the register name and registration number. | Yes |

**S1. PRISMA for abstracts reporting checklist**

| **Section and Topic** | **Item #** | **Checklist item**  **S1. PRISMA reporting checklist** | **Location where item is reported** |
| --- | --- | --- | --- |
| **TITLE** | | |  |
| Title | 1 | Identify the report as a systematic review. | Pg. 1 |
| **ABSTRACT** | | |  |
| Abstract | 2 | See the PRISMA 2020 for Abstracts checklist. | Pg. 1-2 |
| **INTRODUCTION** | | |  |
| Rationale | 3 | Describe the rationale for the review in the context of existing knowledge. | Pg. 3-4 |
| Objectives | 4 | Provide an explicit statement of the objective(s) or question(s) the review addresses. | Pg. 6 |
| **METHODS** | | |  |
| Eligibility criteria | 5 | Specify the inclusion and exclusion criteria for the review and how studies were grouped for the syntheses. | Pg. 6 |
| Information sources | 6 | Specify all databases, registers, websites, organisations, reference lists and other sources searched or consulted to identify studies. Specify the date when each source was last searched or consulted. | Pg. 7 |
| Search strategy | 7 | Present the full search strategies for all databases, registers and websites, including any filters and limits used. | Pg. 7 |
| Selection process | 8 | Specify the methods used to decide whether a study met the inclusion criteria of the review, including how many reviewers screened each record and each report retrieved, whether they worked independently, and if applicable, details of automation tools used in the process. | Pg. 7 |
| Data collection process | 9 | Specify the methods used to collect data from reports, including how many reviewers collected data from each report, whether they worked independently, any processes for obtaining or confirming data from study investigators, and if applicable, details of automation tools used in the process. | Pg. 9 |
| Data items | 10a | List and define all outcomes for which data were sought. Specify whether all results that were compatible with each outcome domain in each study were sought (e.g. for all measures, time points, analyses), and if not, the methods used to decide which results to collect. | Pg. 9 |
|  | 10b | List and define all other variables for which data were sought (e.g. participant and intervention characteristics, funding sources). Describe any assumptions made about any missing or unclear information. | Pg. 9 |
| Study risk of bias assessment | 11 | Specify the methods used to assess risk of bias in the included studies, including details of the tool(s) used, how many reviewers assessed each study and whether they worked independently, and if applicable, details of automation tools used in the process. | Pg. 8 |
| Effect measures | 12 | Specify for each outcome the effect measure(s) (e.g. risk ratio, mean difference) used in the synthesis or presentation of results. | Pg. 9 |
| Synthesis methods | 13a | Describe the processes used to decide which studies were eligible for each synthesis (e.g. tabulating the study intervention characteristics and comparing against the planned groups for each synthesis (item #5)). | Pg. 9 |
|  | 13b | Describe any methods required to prepare the data for presentation or synthesis, such as handling of missing summary statistics, or data conversions. | Pg. 7 |
|  | 13c | Describe any methods used to tabulate or visually display results of individual studies and syntheses. | Pg.10 |
|  | 13d | Describe any methods used to synthesize results and provide a rationale for the choice(s). If meta-analysis was performed, describe the model(s), method(s) to identify the presence and extent of statistical heterogeneity, and software package(s) used. | Pg.10 |
|  | 13e | Describe any methods used to explore possible causes of heterogeneity among study results (e.g. subgroup analysis, meta-regression). | N/A |
|  | 13f | Describe any sensitivity analyses conducted to assess robustness of the synthesized results. | N/A |
| Reporting bias assessment | 14 | Describe any methods used to assess risk of bias due to missing results in a synthesis (arising from reporting biases). | Pg.8 |
| Certainty assessment | 15 | Describe any methods used to assess certainty (or confidence) in the body of evidence for an outcome. | Pg.8 |
| **RESULTS** | | |  |
| Study selection | 16a | Describe the results of the search and selection process, from the number of records identified in the search to the number of studies included in the review, ideally using a flow diagram. | Pg. 11 |
|  | 16b | Cite studies that might appear to meet the inclusion criteria, but which were excluded, and explain why they were excluded. | Pg.12 |
| Study characteristics | 17 | Cite each included study and present its characteristics. | Pg. 79-110 (S6) |
| Risk of bias in studies | 18 | Present assessments of risk of bias for each included study. | Pg.13 |
| Results of individual studies | 19 | For all outcomes, present, for each study: (a) summary statistics for each group (where appropriate) and (b) an effect estimate and its precision (e.g. confidence/credible interval), ideally using structured tables or plots. | Pg. 79-110 (S6) |
| Results of syntheses | 20a | For each synthesis, briefly summarise the characteristics and risk of bias among contributing studies. | Pg.12 |
|  | 20b | Present results of all statistical syntheses conducted. If meta-analysis was done, present for each the summary estimate and its precision (e.g. confidence/credible interval) and measures of statistical heterogeneity. If comparing groups, describe the direction of the effect. | Pg. 14-19 |
|  | 20c | Present results of all investigations of possible causes of heterogeneity among study results. | Pg. 13 |
|  | 20d | Present results of all sensitivity analyses conducted to assess the robustness of the synthesized results. | N/A |
| Reporting biases | 21 | Present assessments of risk of bias due to missing results (arising from reporting biases) for each synthesis assessed. | Pg. 111-117 (S7) |
| Certainty of evidence | 22 | Present assessments of certainty (or confidence) in the body of evidence for each outcome assessed. | Pg.111-117 (S7) |
| **DISCUSSION** | | |  |
| Discussion | 23a | Provide a general interpretation of the results in the context of other evidence. | Pg. 30 |
|  | 23b | Discuss any limitations of the evidence included in the review. | Pg. 32 |
|  | 23c | Discuss any limitations of the review processes used. | Pg. 32 |
|  | 23d | Discuss implications of the results for practice, policy, and future research. | Pg. 32 |
| **OTHER INFORMATION** | | |  |
| Registration and protocol | 24a | Provide registration information for the review, including register name and registration number, or state that the review was not registered. | Pg. 6 |
|  | 24b | Indicate where the review protocol can be accessed, or state that a protocol was not prepared. | Pg. 6 |
|  | 24c | Describe and explain any amendments to information provided at registration or in the protocol. | Pg. 33 |
| Support | 25 | Describe sources of financial or non-financial support for the review, and the role of the funders or sponsors in the review. | Pg. 33 |
| Competing interests | 26 | Declare any competing interests of review authors. | Pg. 33 |
| Availability of data, code and other materials | 27 | Report which of the following are publicly available and where they can be found: template data collection forms; data extracted from included studies; data used for all analyses; analytic code; any other materials used in the review. | Pg. 33 |

**S2. Extracted data items**

**Article characteristics** (title, authors, year, country)

**Study aims** (relevant to the review objectives)

**Study Design** (cross sectional / longitudinal / RCT /Quasi)

**Data collection method**

**Country** [of samples not academic institute]

**Settings and Other Context-Related information** (e.g. cultural, geographical)

**Donation Type** – PBSC /bone marrow / both?

**Donation Stage** – e.g. not yet registered / registered / typing/matched / donated already

**Phenomena of Interest (qualitative studies only)**

**Variables of Interest (quantitative studies only)**

Independent/predictor variables. Include brief detail on measures/scales where appropriate

**Outcome Measures (quantitative only)**– dependent / outcome variables and how they were measured.

**Number of participants**

**Characteristics of participants** – sex (% female) / ethnicity/socio-economic/marital status etc. Report mean and SD where possible.

**Results**

Outcomes or findings of significance to the review objectives.

**For quantitative studies:**

- report all effect sizes (e.g χ^2^, odds-ratio, Pearson’s correlation coefficient (r), t-test etc)

to two decimal places. Mean, SD, Degrees of freedom.

- report p values where available to three decimal places. Drop 0 before the decimal point e.g p=.003.
- ANOVA – report f value, degrees of freedom.
- For purely descriptive studies, report percentages + confidence intervals (if possible)

**For qualitative studies:** describe themes (also a finding when the theme does not convey meaning) and provide illustrative example

**S3. List of included studies**

Abdrbo, A., Hassanein, S., Albajhan, G., & Alsabi, F. (2017). Factors Influencing Hematopoietic Stem Cell Donation. *J Nurs Health Sci*, *6*, 54-61. Retrieved from www.researchgate.net/publication/317658722_Factors_Influencing_Hematopoietic_Stem_Cell_Donation.

Anthias, C., Shaw, B. E., Bruce, J. G., Confer, D. L., Abress, L. K., Dew, M. A., Billen, A., O'Leary, A., Braund, H., & Switzer, G. E. (2020). Role of Race/Ethnicity in Donor Decisions about Unrelated Hematopoietic Progenitor Cell Donation: Exploring Reasons for Higher Attrition among Racial/Ethnic Minorities. *Biology of Blood and Marrow Transplantation*, *26*(3), 593-599. https://doi.org/10.1016/j.bbmt.2019.10.012.

Aurelio, M. T., Aniasi, A., Haworth, S. E., Colombo, M. B., Dimonopoli, T., Mocellin, M. C., Poli, F., Torelli, R., Crespiatico, L., Serafini, M., & Scalamogna, M. (2011). Analysis of the motivation for hematopoietic stem cell donation. *Transplantation Proceedings*, *43*(4), 981-984. https://doi.org/10.1016/j.transproceed.2011.01.128.

Bagcivan, G., Ozen, N., Bayrak, D., & Cinar, F. (2020). Does Being A Cancer Patient or Family Caregiver of A Cancer Patient Affect Stem Cell Donation Awareness? *Asia-Pacific Journal of Oncology Nursing*, *7*(1), 36-43. doi:10.4103/apjon.apjon_22_19.

Bagozzi, R. P., Lee, K., & Van Loo, F. (2001). Decisions to donate bone marrow: the role of attitudes and subjective norms across cultures. *Psychology & Health*, *16*(1), 29-56. https://doi.org/10.1080/08870440108405488.

Balassa, K., Griffiths, A., Winstone, D., Li, Y., Rocha, V., & Pawson, R. (2019). Attrition at the final donor stage among unrelated haematopoietic stem cell donors: the British Bone Marrow Registry experience. *Transfusion Medicine*, *29*(5), 332-337. https://doi.org/10.1111/tme.12613.

Bart, T., Volken, T., Fischer, Y., & Mansouri Taleghani, B. (2014). Giving blood and enrolling on the stem cell donor registry: Ranking of obstacles and motives in Switzerland. *Transfusion Medicine and Hemotherapy*, *41*(4), 264-272. https://doi.org/10.1159/000365457.

Beatty, P. G., Atcher, C., Hess, E., Meyer, D. M., & Slichter, S. J. (1989). Recruiting blood donors into a local bone marrow donor registry. *Transfusion*, *29*(9), 778-782. https://doi.org/10.1046/j.1537-2995.1989.29990070181.x.

Billen, A., Madrigal, J. A., Scior, K., Shaw, B. E., & Strydom, A. (2017). Donation of peripheral blood stem cells to unrelated strangers: A thematic analysis. *PLoS ONE*, *12*(10), -Arte Number: e0186438. https://doi.org/10.1371/journal.pone.0186438.

Branach, B., Tadla, M., & Nurzyńska-Flak, J. (2018). Evaluation of factors affecting the decision to register as a potential bone marrow donor. *Journal of Education, Health and Sport*, *8*(2), 186-195. http://dx.doi.org/10.5281/zenodo.1173111.

Briggs, N. C., Piliavin, J. A., Lorentzen, D., & Becker, G. A. (1986). On willingness to be a bone marrow donor. *Transfusion*, *26*(4), 324-330. https://doi.org/10.1046/j.1537-2995.1986.26486262738.x.

Dasgupta, K. (2018). Generosity and compliance: Recruitment-work and the pathways to participation in bone marrow donation. *Social science & medicine*, *206*, 86-92. https://doi.org/10.1016/j.socscimed.2018.04.012.

Galanis, P. A., Sparos, L. D., Katostaras, T., Velonakis, E., & Kalokerinou, A. (2008). Factors That Influence Greeks' Decision to Register as Potential Bone Marrow Donors. *Transplantation Proceedings*, *40*(5), 1271-1274. https://doi.org/10.1016/j.transproceed.2008.03.139.

Glasgow, M. E. S., & Bello, G. (2007). Bone marrow donation: factors influencing intentions in African Americans. *Oncology Nursing Forum*, *34*(2), 369-377. https://doi.org/10.1016/j.profnurs.2009.01.004.

Hazzazi, A. A., Ageeli, M. H., Alfaqih, A. M., Zakri, A. K., & Elmakki, E. E. (2019). Knowledge and attitude towards hematopoietic stem cell transplantation among medical students at Jazan University, Saudi Arabia. *Saudi medical journal*, *40*(10), 1045-1051. doi:10.15537/smj.2019.10.24294.

Holroyd, E., & Molassiotis, A. (2000). Hong Kong Chinese perceptions of the experience of unrelated bone marrow donation. *Social science & medicine*, *51*(1), 29-40. https://doi.org/10.1016/S0277-9536(99)00427-X.

Hyde, M. K., Mclaren, P. J., & White, K. M. (2014). Identifying belief targets to increase bone marrow registry participation among students who have never donated blood. *Psychology, health & medicine*, *19*(1), 115-125. https://doi.org/10.1080/13548506.2013.775467.

Hyde, M. K., & White, K. M. (2013). Testing an extended theory of planned behavior to predict young people's intentions to join a bone marrow donor registry. *Journal of Applied Social Psychology*, *43*(12), 2462-2467. https://doi.org/10.1111/jasp.12195.

Kaster, E. C., Rogers, C. R., Kwon, C. J., & Rosen, B. (2014). Getting to the Heart of Being the Match: A Qualitative Analysis of Bone Marrow Donor Recruitment and Retention among College Students. *Health Educator*, *46*(1), 14-19. Retrieved from https://www.ncbi.nlm.nih.gov/pmc/articles/PMC4306578/.

Kwok, J., Leung, E., Wong, W., Leung, K., Lee, C. K., Lam, W., & Ip, P. (2015). Factors influencing hematopoietic stem cell donation intention in Hong Kong: A web-based survey. *Annals of Transplantation*, *20*, 604-613. doi: 10.12659/AOT.894165.

La Casta, A. H., Shaw, B. E., Anthias, C., Bruce, J. G., Pastorek, G., Billen, A., O’Leary, A., & Switzer, G. E. (2019). Motives for joining an unrelated HSC donor registry: description, categorization, and association with donor availability. *Bone Marrow Transplantation*, *54*(3), 425-431. https://doi.org/10.1038/s41409-018-0278-2.

Laver, J. H., Hulsey, T. C., Jones, J. P., Gautreaux, M., Barredo, J. C., & Abboud, M. R. (2001). Assessment of barriers to bone marrow donation by unrelated African-American potential donors. *Biology of Blood and Marrow Transplantation*, *7*(1), 45-48. https://doi.org/10.1053/bbmt.2001.v7.pm11215698.

Lee-Won, R. J., Abo, M. M., Na, K., & White, T. N. (2016). More than numbers: Effects of social media virality metrics on intention to help unknown others in the context of bone marrow donation. *Cyberpsychology, Behavior, and Social Networking*, *19*(6), 404-411. https://doi.org/10.1089/cyber.2016.0080.

Li, X., Liu, S., Liu, C., Du, S., & Cong, Y.. (2021). Regional Differences Among Bone Marrow Registrants: The Results of a Cross-Sectional Telephone-Based Survey. *Transplantation Proceedings*, *53*(2), 724–729. https://doi.org/10.1016/j.transproceed.2021.01.029.

Lindsey, L. L. M. (2005). Anticipated Guilt as Behavioral Motivation An Examination of Appeals to Help Unknown Others Through Bone Marrow Donation. *Human Communication Research*, *31*(4), 453-481. https://doi.org/10.1111/j.1468-2958.2005.tb00879.x.

Lown, R., Marsh, S., Switzer, G., Latham, K., Madrigal, J., & Shaw, B. (2014). Ethnicity, length of time on the register and sex predict donor availability at the confirmatory typing stage. *Bone Marrow Transplantation*, *49*(4), 525-531. https://doi.org/10.1038/bmt.2013.206.

McCullough, J., Rogers, G., & Dahl, R. (1986). Development and operation of a program to obtain volunteer bone marrow donors unrelated to the patient. *Transfusion*, *26*(4), 315-323. https://doi.org/10.1046/j.1537-2995.1986.26486262737.x

Mclaren, P. J., Hyde, M. K., & White, K. M. (2012). Exploring the role of gender and risk perceptions in people’s decisions to register as a bone marrow donor. *Health education research*, *27*(3), 513-522. https://doi.org/10.1093/her/cyr112.

Milaniak, I., Rużyczka, E. W., & Przybyłowski, P.. (2020). Factors Influencing Decision Making About Living Donation Among Medical Students. *Transplantation Proceedings*, *52*(7), 1994–2000. https://doi.org/10.1016/j.transproceed.2020.03.049.

Monaghan, M., Yi, Q. L., Green, M., Campbell, T., Weiss, J. T., Dibdin, N., Mercer, D., Elmoazzen, H., & Allan, D. S. (2021). Factors associated with registrant availability for unrelated adult donor hematopoietic stem cell donation: Analysis of the stem cell registry at Canadian Blood Services. *Transfusion*, *61*(1), 24-28. https://doi.org/10.1111/trf.16129.

Narayanan, P., Wolanskyj, A., Ehlers, S. L., Litzow, M. R., Patnaik, M. S., Hogan, W. J., & Hashmi, S. K. (2016). Medical Students' Knowledge, Familiarity, and Attitudes towards Hematopoietic Stem Cell Donation: Stem Cell Donation Behaviors. *Biology of Blood and Marrow Transplantation*, *22*(9), 1710-1716. https://doi.org/10.1016/j.bbmt.2016.06.014.

Norvilitis, J. M., & Riley, T. M. (2001). Exploring the motivations of bone marrow typing donors. *Journal of Psychosocial Oncology*, *19*(1), 49-62. https://doi.org/10.1300/J077v19n01_04.

O’Donnell, N. H., & Guidry, J. P. (2020). # BeTheMatch: Assessing How Testimonials on Reddit Promote the Importance of Donating Bone Marrow. *Journal of Health Communication*, 1-11. https://doi.org/10.1080/10810730.2020.1836088.

Onitilo, A. A., Lin, Y. H., Okonofua, E. C., Afrin, L. B., Ariail, J., & Tilley, B. C. (2004). Race, education, and knowledge of bone marrow registry: Indicators of willingness to donate bone marrow among African Americans and Caucasians. *Transplantation Proceedings*, *36*(10), 3212-3219. https://doi.org/10.1016/j.transproceed.2004.10.019.

Sarason, I. G., Sarason, B. R., Slichter, S. J., Beatty, P. G., Meyer, D. M., & Bolgiano, D. C. (1993). Increasing participation of blood donors in a bone-marrow registry. *Health Psychology*, *12*(4), 272-276. https://doi.org/10.1037/0278-6133.12.4.272.

Sikora, A., Wiorkowski, K., Szara, P., & Drabko, K. (2014). Knowledge and attitude of Lublin universities students' toward the opportunity of becoming unrelated bone marrow donor. *Folia medica Cracoviensia*, *54*(2), 27-33. Retrieved from http://www.fmc.cm-uj.krakow.pl/pdf/54_2_27.pdf.

Simmons, R. G., Schimmel, M., & Butterworth, V. A. (1993). The self-image of unrelated bone marrow donors. *Journal of Health and Social Behavior*, 285-301. https://doi.org/10.2307/2137368.

Stroncek, D., Strand, R., Scott, E., Kamstra-halvorson, L., Halagan, N., Rogers, G., & Mccullough, J. (1989). Attitudes and physical condition of unrelated bone marrow donors immediately after donation. *Transfusion*, *29*(4), 317-322. https://doi.org/10.1046/j.1537-2995.1989.29489242797.x.

Studts, J. L., Ruberg, J. L., McGuffin, S. A., & Roetzer, L. M. (2010). Decisions to register for the National Marrow Donor Program: Rational vs emotional appeals. *Bone Marrow Transplantation*, *45*(3), 422-428. https://doi.org/10.1038/bmt.2009.174.

Switzer, G. E., Bruce, J. G., Myaskovsky, L., Dimartini, A., Shellmer, D., Confer, D. L., Abress, L. K., King, R. J., Harnaha, A. G., Ohngemach, S., & Dew, M. A. (2013). Race and ethnicity in decisions about unrelated hematopoietic stem cell donation. *Blood*, *121*(8), 1469-1476. https://doi.org/10.1182/blood-2012-06-437343.

Switzer, G. E., Dew, M. A., Butterworth, V. A., Simmons, R. G., & Schimmel, M. (1997). Understanding donors' motivations: a study of unrelated bone marrow donors. *Social science & medicine*, *45*(1), 137-147. https://doi.org/10.1016/S0277-9536(96)00327-9.

Switzer, G. E., Dew, M. A., Goycoolea, J. M., Myaskovsky, L., Abress, L., & Confer, D. L. (2004). Attrition of potential bone marrow donors at two key decision points leading to donation. *Transplantation*, *77*(10), 1529-1534. doi:10.1097/01.TP.0000122219.35928.D6.

Switzer, G. E., Dew, M. A., Harrington, D. J., CrowleyMatoka, M., Myaskovsky, L., Abress, L., & Confer, D. L. (2005). Ethnic differences in donation-related characteristics among potential hematopoietic stem cell donors. *Transplantation*, *80*(7), 890-896. doi:10.1097/01.TP.0000173648.60978.30.

Switzer, G. E., Dew, M. A., Stukas, A. A., Goycoolea, J. M., Hegl, , J., & Simmons, R. G. (1999). Factors associated with attrition from a national bone marrow registry. *Bone Marrow Transplantation*, *24*(3), 313-319. https://doi.org/10.1038/sj.bmt.1701884.

Switzer, G. E., Myaskovsky, L., Goycoolea, J. M., Dew, M. A., Confer, D. L., & King, R. (2003). Factors associated with ambivalence about bone marrow donation among newly recruited unrelated potential donors. *Transplantation*, *75*(9), 1517-1523. doi:10.1097/01.TP.0000060251.40758.98.

Ting, C. Y., Lee, Y. F., Lim, C. J., Ting, R. S. K., Zahrin, M. M. M., Ahmad, A. S., Wong, J. S. Y., Arip, M., Zakaria, Z., & Adruce, S. A. Z. (2020). Factors Associated with Intention to Donate Hematopoietic Stem Cells among Blood Donors. *Transfusion Medicine and Hemotherapy*, 1-7. https://doi.org/10.1159/000512197.

Tuszynska-Bogucka, W. (2019). 'Be the match'. Predictors of decisions concerning registration in potential bone marrow donor registry in a group of Polish young adults as an example of prosocial behaviour. *Current Psychology: A Journal for Diverse Perspectives on Diverse Psychological Issues*, *38*(4), 1042-1061. https://doi.org/10.1007/s12144-019-00319-5.

Varghese, S., & Hem, M. (2015). Peripheral blood stem cell donation awareness among college students. *International Journal of Science, Technology & Management*. Retrieved from http://www.ijstm.com/images/short_pdf/1433173321_P1-5.pdf.

Vasconcellos, A., Nunes, A., & Feller, E. (2011). Knowledge, attitudes, and behaviors regarding the bone marrow registry among college and medical students in Rhode Island. *Medicine and health, Rhode Island*, *94*(10), 302-305. Retrieved from http://www.rimed.org/medhealthri/2011-10/2011-10-302.pdf.

Vekaria, K. M., Hammell, A. E., Vincent, L., Smith, M., Rogers, T., Switzer, G. E., & Marsh, A. A. (2020). The Role of Prospection in Altruistic Bone Marrow Donation Decisions. *Health Psychology*, *39*(4), 316-324. https://doi.org/10.1037/hea0000819.

**S4:**

A list of all studies excluded after full text examination including the explicit reasons for exclusion.

| **Study** | **Reason for Exclusion** |
| --- | --- |
| Adebiyi, S., Okoye, I., & Ocheni, S. (2013). Bone Marrow Registry In Nigeria: Building The Road to Greater HLA Diversity: P19. *Tissue Antigens*, *81*(5), 308-309. | Wrong publication type |
| Akiyama, H., Hara, M., Hino, M., Sao, H., Hoshi, Y., Ohto, H., & Kai, S. (2006). Second donation of bone marrow: results from the Japan Marrow Donor Program (JMDP). *Bone marrow transplantation*, *37*(8), 795-796. | Wrong publication type |
| Alfraidy, O. B., Alharbi, H. H., Alrehaili, M. N., Alfridy, M. B., Alkabli, R. S., & Alwasaidi, T. A. (2016). Rising of public medical awareness is the most important motivating factor for hematopoietic stem cell donation in Saudi Arabia. | Wrong population |
| Almaeen, A., Wani, F. A., & Thirunavukkarasu, A. (2021). Knowledge and attitudes towards stem cells and the significance of their medical application among healthcare sciences students of Jouf University. *PeerJ*, *9*, e10661. | Wrong outcome |
| Altiok, M., Gozudeli, G., Durmaz, S., Yeral, M., Boga, C., & Ozdogu, H. (2012). The efficacy of the audovisual method used on the subject of stem cell transplantation for patient/donor informatory meetings: P831. *Bone Marrow Transplantation*, *47*. | Wrong publication type |
| Altobelli, L. (2012). An altruistic living donor’s story. *Narrative inquiry in bioethics*, *2*(1), 7-10. | Wrong population |
| Alvarez, D. H., & Hewitt, J. B. (1995). Bone Marrow Screening: Successful Program. *AAOHN Journal*, *43*(7), 376-378. | Wrong outcome |
| Alves, H. (2009). Education for health in schools promoting bone marrow donation: P86. *Tissue Antigens*, *73*(5). | Wrong publication type |
| Alves, H., Mota, J., Godinho, F., & Reis, R. (2011). Education for health on bone marrow donation-the Oporto model: P232. *Tissue Antigens*, *77*(5), 485-486. | Wrong publication type |
| Alves, H., & Reis, R. P. (2012). Virtual Municipal Banks of Cell Donors-An innovative concept to promote Education for Donation in Transplantation and Disease Prevention: 2642. *Transplantation*, *94*(10S), 530. | Wrong publication type |
| Alves, H., Reis, R., & Mota, J. (2011). Education For Health In Schools, Introducing Transplantation-The Oporto Model: RO-306. *Transplant International*, *24*. | Wrong publication type |
| Ami, S. B., Zinger, R., & Ashkenazi, T. (2003, March). Is the motivation level of volunteers active in organ donation promotion affected by the training method? In *Transplantation proceedings* (Vol. 35, No. 2, pp. 596-597). | Wrong publication type |
| Anderson, M. F. (1992). Encouraging Bone Marrow Transplants from Unrelated Donors: Some Proposed Solutions to a Pressing Social Problem. *U. Pitt. L. Rev.*, *54*, 477. | Wrong publication type |
| Andev, R. S., & Field, M. (2015). Knowledge and opinions held by British and Canadian Sikhs regarding solid organ and stem cell transplantation. *International Journal of Surgery*, *1*(23), S108. | Wrong publication type |
| Andev, R., Field, M., & Nath, J. (2015). A Comparative Analysis Of British And Canadian Sikh Opinion And Knowledge Regarding Organ And Stem Cell Donation: P14. *Transplant International*, *28*. | Wrong publication type |
| Jacob Arriola, K. R., Perryman, J. P., Doldren, M. A., Warren, C. M., & Robinson, D. H. (2007). Understanding the role of clergy in African American organ and tissue donation decision-making. *Ethnicity and Health*, *12*(5), 465-482. | Wrong population |
| Assari, S. (2014). Celebrities as role models to increase national organ donation in Iran. *Exp Clin Transplant*, *12*, 388-389. | Wrong publication type |
| Atiemo, K., & Ladner, D. P. (2015). Active education, keeping it personal and making it easy: a potential path to increasing donors. *Transplantation*, *99*(12), 2448. | Wrong publication type |
| Bagcivan, G., Ozen, N., Bayrak, D., & Cınar, F. I. (2018). Does being cancer patient or family caregiver of cancer patient effect bone marrow donation awareness? *Annals of Oncology*, *29*, viii691. | Wrong publication type |
| Baker, F., Curbow, B., & Wingard, J. R. (1991). Role retention and quality of life of bone marrow transplant survivors. *Social science & medicine*, *32*(6), 697-704. | Wrong population |
| Bakken, R., Van Walraven, A. M., & Egeland, T. (2004). Donor commitment and patient needs. *Bone Marrow Transplantation*, *33*(2), 225-230. | Wrong publication type |
| Balassa, K., Griffiths, A., Winstone, D., Li, Y., Rocha, V., & Pawson, R. (2017, June). Unrelated donor attrition at a late stage: the British bone marrow registry experience. In *Haematologica* (Vol. 102, pp. 625-625). | Wrong publication type |
| Ballen, K. K., Hicks, J., Dharan, B., Ambruso, D., Anderson, K., Bianco, C., ... & Wingard, J. R. (2002). Racial and ethnic composition of volunteer cord blood donors: comparison with volunteer unrelated marrow donors. *Transfusion*, *42*(10), 1279-1284. | Wrong population |
| Bart, T (2012). Motivation for Blood Stem Donation. *Proceedings of the Transfusion Medicine and Hemotherapy Conference 2012,* Austria, 39 (0), 15. | Wrong publication type |
| Bennett, R., & Savani, S. (2004). Factors influencing the willingness to donate body parts for transplantation. *Journal of health & social policy*, *18*(3), 61-85. | Wrong population |
| Beom, S. H., Kim, E. J., Kim, M., & Kim, T. G. (2016). Unrelated hematopoietic stem cell registry and the role of the Hematopoietic Stem Cell Bank. *Blood research*, *51*(2), 107-112. | Wrong publication type |
| Bergstrom, T. C., Garratt, R. J., & Sheehan-Connor, D. (2009). One chance in a million: Altruism and the bone marrow registry. *American Economic Review*, *99*(4), 1309-34. | Wrong publication type |
| Bhengu, B. R., & Uys, H. H. M. (2004). Organ donation and transplantation within the Zulu culture. *Curationis*, *27*(3), 24-33. | Wrong population |
| Bonaiuto, F., Cancellieri, U. G., Ariccio, S., Molinario, E., Pierelli, L., & Bonaiuto, M. (2020). Why donate stem cells? A pilot validation of new measures for studying antecedents of stem cell donation intention. RICERCHE DI PSICOLOGIA. | Wrong outcome |
| Boulware, L., Ratner, L. E., Cooper, L. A., Sosa, J. A., LaVeist, T. A., & Powe, N. R. (2002). Understanding disparities in donor behavior: race and gender differences in willingness to donate blood and cadaveric organs. *Medical care*. | Wrong population |
| Boulware, L. E., Ratner, L. E., Sosa, J. A., Cooper, L. A., LaVeist, T. A., & Powe, N. R. (2002). Determinants of willingness to donate living related and cadaveric organs: identifying opportunities for intervention. *Transplantation*, *73*(10), 1683-1691. | Wrong population |
| Bräuninger, S., Thorausch, K., Luxembourg, B., Schulz, M., Chow, K. U., Seifried, E., & Bonig, H. (2014). Deferrals of volunteer stem cell donors referred for evaluation for matched-unrelated stem cell donation. *Bone marrow transplantation*, *49*(11), 1419-1425. | Wrong outcome |
| Bredeson, C., Leger, C., Couban, S., Simpson, D., Huebsch, L., Walker, I., ... & Lipton, J. (2004). An evaluation of the donor experience in the Canadian multicenter randomized trial of bone marrow versus peripheral blood allografting. *Biology of Blood and Marrow Transplantation*, *10*(6), 405-414. | Wrong outcome |
| Buhler, S., Nunes, J. M., Nicoloso, G., Tiercy, J. M., & Sanchez-Mazas, A. (2012). The Analysis Of The Swiss Bone Marrow Donor Registry And Its Applications: Software Development For Handling Ambiguous HLA Data, Optimizing Donor Recruitment Strategies And Defining Reliable Reference Data Panels For Population Genetics And Epidemiology: P323. *Tissue Antigens*, *79*(6), 564-565. | Wrong publication type |
| Butterworth, V. A., Simmons, R. G., Bartsch, G., Randall, B., Schimmel, M., & Stroncek, D. F. (1993). Psychosocial effects of unrelated bone marrow donation: experiences of the National Marrow Donor Program. | Wrong outcome |
| Butterworth, V. A., Simmons, R. G., & Schimmel, M. (1993). When altruism fails: reactions of unrelated bone marrow donors when the recipient dies. *OMEGA-Journal of Death and Dying*, *26*(3), 161-173. | Wrong outcome |
| Cacioppo, J. T., & Gardner, W. L. (1993). What underlies medical donor attitudes and behavior? *Health Psychology*, *12*(4), 269. | Wrong publication |
| Chabalwski, F., & Norris, M. G. (1994). The gift of life: talking to families about organ and tissue donation. *AJN The American Journal of Nursing*, *94*(6), 28-33. | Wrong population |
| Chen, S. H., Yang, S. H., Chu, S. C., Tsai, S. S., Chang, C. Y., Chiu, Y. W., ... & Wang, T. F. (2011). Second donation from volunteer hematopoietic stem cell donors in Taiwan. *Tzu Chi Medical Journal*, *23*(1), 16-19. | Wrong outcome |
| Confer, D. L. (2001). The National Marrow Donor Program: meeting the needs of the medically underserved. *Cancer: Interdisciplinary International Journal of the American Cancer Society*, *91*(S1), 274-278. | Wrong publication type |
| Confer, D. L., Abress, L. K., Navarro, W., & Madrigal, A. (2010). Selection of adult unrelated hematopoietic stem cell donors: beyond HLA. *Biology of Blood and Marrow Transplantation*, *16*(1), S8-S11. | Wrong publication type |
| De Vries, R. G., Tomlinson, T., Kim, H. M., Krenz, C. D., Ryan, K. A., Lehpamer, N., & Kim, S. Y. (2016). The moral concerns of biobank donors: the effect of non-welfare interests on willingness to donate. *Life sciences, society and policy*, *12*(1), 1-15. | Wrong population |
| Dedhia, L., & Parekh, S. (2014). Challenges faced by bone marrow registries in India. *Indian Journal of Transplantation*, *8*(3), 80-83. | Wrong publication type |
| Edinger, W. (1990). Respect for donor choice and the Uniform Anatomical Gift Act. *Journal of Medical Humanities*, *11*(3), 135-142. | Wrong publication type |
| Edwards, N. E., Ellingwood, A., Hebdon, M., Foli, K. J., & Freeman, J. L. (2014). Guiding patient decision-making regarding bone marrow donation. *The Journal for Nurse Practitioners*, *10*(2), 113-119. | Wrong publication type |
| El Fergougui, S., Bordoni, C., Fettah, O., Blaise, C., Chafai, S., Coustaud, E., ... & Di Cristofaro, J. (2014). Registry Genetic Diversity: How To Increase Bone Marrow Donation In Marseilles?: P238. *Tissue Antigens*, *84*(1). | Wrong publication type |
| Ejiugwo, M., Shaw, G., Barry, F., Krawczyk, J., & McInerney, V. (2019). The motivational factors and adverse events experienced by healthy volunteers donating bone marrow for research. | Wrong population |
| Fingrut, W., Messner, H. A., & Allan, D. (2020). Targeted recruitment of optimal donors for unrelated hematopoietic cell transplantation: The Stem Cell Club process. *Hematology/oncology and stem cell therapy*, *13*(4), 220-231. | Wrong publication type |
| Fingrut, W., Rikhraj, K., & Allan, D. (2018). Targeted recruitment of male donors for allogeneic haematopoietic cell transplantation: A review of the evidence. *Vox sanguinis*, *113*(4), 307-316. | Wrong publication type |
| Fingrut, W., Parmar, S., Cuperfain, A., Rikhraj, K., Charman, E., Ptak, E., ... & Messner, H. (2017). The Stem Cell Club: a model for unrelated stem cell donor recruitment. *Transfusion*, *57*(12), 2928-2936. | Wrong publication type |
| Foster, T., Moore, L., & Gammon, R. (2012). Optimizing Recruitment Of Potential Volunteer Hematopoietic Progenitor Cell Donors At Large Blood Drives: 54. *Journal of Clinical Apheresis*, *27*(1), 34-35. | Wrong publication type |
| Freytes, C. O., & Beatty, P. G. (1996). Representation of Hispanics in the National Marrow Donor Program. *Bone marrow transplantation*, *17*(3), 323-327. | Wrong outcome |
| Gandini, A., Roata, C., Franchini, M., Agostini, E., Guizzardi, E., Giacometti, P., ... & Aprili, G. (2001). Unrelated allogeneic bone marrow donation: short-and long-term follow-up of 103 consecutive volunteer donors. *Bone marrow transplantation*, *28*(4), 369-374. | Wrong outcome |
| Garcia, M. C., Chapman, J. R., Shaw, P. J., Gottlieb, D. J., Ralph, A., Craig, J. C., & Tong, A. (2013). Motivations, experiences, and perspectives of bone marrow and peripheral blood stem cell donors: thematic synthesis of qualitative studies. *Biology of Blood and Marrow Transplantation*, *19*(7), 1046-1058. | Wrong publication type |
| Garratty, G., Glynn, S. A., McEntire, R., & Retrovirus Epidemiology Donor Study. (2004). ABO and Rh (D) phenotype frequencies of different racial/ethnic groups in the United States. *Transfusion*, *44*(5), 703-706. | Wrong population |
| Garrett, D., & Yoder, L. H. (2007). An overview of stem cell transplant as a treatment for cancer. *MedSurg Nursing*, *16*(3), 183. | Wrong publication type |
| Gillespie, I. (2018). *Increasing Blacks’ Representation and Utilization on the Bone Marrow Registry: An Action-oriented Needs Assessment* (Doctoral dissertation, University of the Pacific). | Wrong publication type |
| Gutierrez-Aguirre, C. H., Cantu Rodriguez, O. G., De la Garza-Salazar, F., Salazar Riojas, R., Gomez De Leon, A., Colunga Pedraza, P. R., ... & Mancias, C. (2019). Moral distress, anxiety, and others physical symptoms related to hematopoietic stem cell donation. | Wrong population |
| Haberman, M. R. (1988, February). Psychosocial aspects of bone marrow transplantation. In *Seminars in oncology nursing,* 4 (1), ,55-59. | Wrong population |
| Hayre-Edwards, R. R. (2020). Increasing minority bone marrow donors: a transmedia storyworld to increase donor populations and spread awareness. | Wrong publication type |
| Heath, A. (2012). The essence of giving—a transplant story. *Narrative inquiry in bioethics*, *2*(1), 14-17. | Wrong publication type |
| Heemskerk, M. B. A., Van Walraven, S. M., Cornelissen, J. J., Barge, R. M. Y., Bredius, R. G. M., Egeler, R. M., ... & Oudshoorn, M. (2005). How to improve the search for an unrelated haematopoietic stem cell donor. Faster is better than more! *Bone marrow transplantation*, *35*(7), 645-652. | Wrong outcome |
| Heinemann, F. M., Wagner, B., Riebschlaege, S., Heinold, A., Baumgart, C., Kordelas, L., Klingberg, C. J., Gebhardt, K., Fischer, J., Enczmann, J., Balz, V., Zeiler, T., Reimer, T., Lenz, V., Horn, P. A. (2019). Recruiting refugees and migrants as new potential blood stem cell donors: Status update of the BluStar. NRW project in North Rhine Westphalia. Oral Sessions. *HLA*, *94*(S1), 9–16. <https://doi.org/10.1111/tan.13634>. | Wrong publication type |
| Herz, S. E. (1999). Two steps to three choices: a new approach to mandated choice. *Cambridge Quarterly of Healthcare Ethics*, *8*(3), 340-347. | Wrong publication type |
| Hessing, D. J., & Elffers, H. (1985). General and physical self-esteem and altruistic behavior. *Psychological reports*. | Wrong publication type |
| Houri, L. F., de Oliveira, C. D., de Souza, C. V., de Moura, M. R., de Araújo Ferreira, L. M., Oliveira, V. D. M. M., & Pereira, W. A. (2012, October). Intentionality of organ/tissues donation for transplantation within a Brazilian hospital complex. In *Transplantation* proceedings, 44 (8), 2272-2275. | Wrong population |
| Hyde, M. K., Knowles, S. R., & White, K. M. (2013). Donating blood and organs: using an extended theory of planned behavior perspective to identify similarities and differences in individual motivations to donate. *Health education research*, *28*(6), 1092-1104. | Wrong population |
| Hyde, M. K., & White, K. M. (2010). Exploring donation decisions: beliefs and preferences for organ donation in Australia. *Death Studies*, *34*(2), 172-185. | Wrong population |
| Iuliani, O., Passeri, C., Papola, F., & Accorsi, P. (2020). The withdrawal of consent to haematopoietic stem cells (HSC) donation: The complicated balance between donor’s rights and patient’s protection. *Transfusion and Apheresis Science*, *59*(4), 102813. | Wrong publication type |
| Kabaila, R. (2016). Donating to save others. *Australian Nursing and Midwifery Journal*, *23*(11), 37. | Wrong publication type |
| Kaya, Z., Gültekin, K. E., Demirtaş, O. K., Karadeniz, D., Çalapkulu, Y., & Tap, Ö. (2015). Effects of targeted education for first-year university students on knowledge and attitudes about stem cell transplantation and donation. *Experimental and Clinical Transplantation*, *13*(1), 76-81. | Wrong study design |
| Kim, M., & Shin, M. (2019). Effect of educational program on knowledge, Attitude, and willingness of nursing students for hematopoietic stem-cell donation. *International journal of environmental research and public health*, *16*(19), 3696. | Wrong study design |
| Kim, M., Kim, T. G., & Beom, S. H. (2020). Physical and Psychological Discomfort Experienced by Hematopoietic Stem-Cell Donors. International journal of environmental research and public health, 17(7), 2316. | Wrong outcome |
| Kita, Y., Ueshima, H., Okayama, A., & Yamakawa, M. (1993). Factors influencing self-registration in a bone-marrow bank: analysis of data from a population survey in Shiga Prefecture. [Nihon koshu eisei zasshi] Japanese journal of public health, 40(11), 1038-1046. | Wrong language |
| Kisch, A. M., Forsberg, A., Fridh, I., Almgren, M., Lundmark, M., Lovén, C., ... & Lennerling, A. (2018). The meaning of being a living kidney, liver, or stem cell donor—a meta-ethnography. *Transplantation*, *102*(5), 744-756. | Wrong publication type |
| Kong, J. H., Hu, Y., Shim, H., Lee, E., Lee, H., Eom, H. S., ... & Kong, S. Y. (2020). Analysis of factors associated with successful allogeneic peripheral blood stem cell collection in healthy donors. *Transfusion and Apheresis Science*, *59*(2), 102679. | Wrong outcome |
| Korbling, M., Przepiorka, D., Gajewski, J., Champlin, R. E., & Chan, K. W. (1995). With first successful allogeneic transplantations of apheresis-derived hematopoietic progenitor cells reported, can the recruitment of volunteer matched, unrelated stem cell donors be expanded substantially? | Wrong publication type |
| Kwon, S. Y., Lee, M. J., Lee, J. H., & Cho, N. S. (2016). Unrelated hematopoietic stem cell donor recruitment of the Korean Red Cross: 20 years experience. The Korean Journal of Blood Transfusion, 27(3), 285-295. | Wrong language |
| Lacetera, N., Macis, M., & Stith, S. S. (2014). Removing financial barriers to organ and bone marrow donation: The effect of leave and tax legislation in the US. *Journal of health economics*, *33*, 43-56. | Wrong outcome |
| Lee, M. H., Jang, J. H., Min, H. J., Jang, H. I., Nah, J. H., Lyu, C. J., ... & Kim, I. (2017). Predictors of general discomfort, limitations in activities of daily living and intention of a second donation in unrelated hematopoietic stem cell donation. *Bone marrow transplantation*, *52*(2), 258-263. | Wrong outcome |
| Lenz, V., Wagner, B., Baumgart, C., Kordelas, L., Klingberg, J. C., Gebhardt, K., ... & Horn, P. A. (2019, January). BluStar. NRW-a project for typing refugees and migrants as potential blood and stem cell donors. In *Oncology Research And Treatment* (Vol. 42, pp. 234-234). | Wrong publication type |
| Li, E. W., Lee, A., Vaseghi-Shanjani, M., Anagnostopoulos, A., Jagelaviciute, G., Kum, E., ... & Fingrut, W. (2020). Development and evaluation of a whiteboard video series to support the education and recruitment of committed unrelated donors for hematopoietic stem cell transplantation. *Biology of Blood and Marrow Transplantation*, *26*(11), 2155-2164. | Wrong study design |
| Li, P. K., Lin, C. K., Lam, P. K., Szeto, C. C., Lau, J. T., Cheung, L., ... & Ko, W. M. (2001). Attitudes about organ and tissue donation among the general public and blood donors in Hong Kong. *Progress in transplantation*, *11*(2), 98-103. | Wrong population |
| Li, Y., Masiliune, A., Winstone, D., Gasieniec, L., Wong, P., Lin, H., ... & Hadley, A. (2020). Predicting the Availability of Hematopoietic Stem Cell Donors Using Machine Learning. *Biology of Blood and Marrow Transplantation*, *26*(8), 1406-1413. | Wrong outcome |
| Li, E. W., Lee, A., Vaseghi‐Shanjani, M., Anagnostopoulos, A., Jagelaviciute, G., Kum, E., ... & Fingrut, W. (2021). Multimedia resources to support the recruitment of committed hematopoietic stem cell donors: Perspectives of the most‐needed donors. Transfusion, 61(1), 274-285. | Wrong outcome |
| Liao, G., Gilmore, K., Steed, A., Elmoazzen, H., & Allan, D. S. (2020). Willingness of volunteers from Canadian Blood Service’s Stem Cell Registry to donate blood, marrow, and other tissues for regenerative therapy. *Transfusion*, *60*(3), 582-587. | Wrong outcome |
| Lindsey, L. L. M., Yun, K. A., & Hill, J. B. (2007). Anticipated guilt as motivation to help unknown others: An examination of empathy as a moderator. *Communication Research*, *34*(4), 468-480. | Wrong outcome |
| Lim, S., Cho, S., & Yang, E. (2020). The Intetions of University Students Regarding Donating Hematopoietic Stem Cells Based on the Theory of Planned Behavior. Journal of muscle and joint health, 27(2), 153-159. | Wrong language |
| Loginova, M., Malysheva, N., Minaeva, N., Paramonov, I. (2019). Assessment of the efficiency of the activity of the bone marrow donor registry. Proceedings of the 13^th^ East-West Immunogenetics Conference Ewic 2019 – Building Bridges. *HLA,  94(S2), 49–76.* [*https://doi.org/10.1111/tan.13752*](https://doi.org/10.1111/tan.13752). | Wrong publication type |
| Manley, H., Sprinks, J., & Breedon, P. (2019). Menstrual Blood-Derived Mesenchymal Stem Cells: Women’s Attitudes, Willingness, and Barriers to Donation of Menstrual Blood. *Journal of Women’s Health*, *28*(12), 1688-1697. | Wrong population |
| McElligott, M. C., Menitove, J. E., & Aster, R. H. (1986). Recruitment of unrelated persons as bone marrow donors. A preliminary experience. *Transfusion*, *26*(4), 309-314. | Wrong publication type |
| Mc Kenzie, L. (2013). Psychosocial factors that influence sibling donors during allogeneic bone marrow transplantation (Doctoral dissertation, Stellenbosch: Stellenbosch University). | Wrong language |
| Mengling, T., Cotta, L., Schmidt, A. H., & Ehninger, G. (2011). Why Work-Up Requests for HSCT Donors Fail–Reasons and Ways to Improve from a Donor Center Perspective. *Biology of Blood and Marrow Transplantation*, *17*(2), S307-S308. | Wrong publication type |
| Molassiotis, A., & Holroyd, E. (1999). Assessment of psychosocial adjustment in Chinese unrelated bone marrow donors. Bone marrow transplantation, 24(8), 903-910. | Wrong outcome |
| Mueller, C. R., Feldmann, U., Bochtler, W., Morsch, S., & Schmidt, A. (2012). 114-P: The Effect Of Age, Gender And Typing Resolution On The Probability Of Stem Cell Donation. *Human immunology*, *73*, 121. | Wrong publication type |
| Murata, M., Haneda, M., Nishida, T., Kanie, T., Hamaguchi, M., Minami, S., & Kodera, Y. (1998). Unrelated donor bone marrow transplantation in Japanese patients is facilitated by the national marrow donor program of the United States. In *Transplantation* proceedings, 1 (30), 150-152. | Wrong outcome |
| Murphy, E. A., Ferguson, S. S., Omondi, N. A., Getzendaner, L. C., Gajewski, J. L., Goldstein, G. A., ... & Mahjail, N. S. (2010). The National Marrow Donor Program’s symposium on patient advocacy in cellular transplantation therapy: addressing barriers to hematopoietic cell transplantation. *Biology of Blood and Marrow Transplantation*, *16*(2), 147-156. | Wrong publication type |
| Muzaffar, M., Ghose, A., Shah, S., & Chaudhary, R. (2012). Bone marrow donation perceptions among healthcare workers: a survey at University of Toledo Medical Center. *Hematological oncology*, *30*(1), 53-55. | Wrong publication type |
| Myaskovsky, L., Switzer, G. E., Dew, M. A., Goycoolea, J. M., Confer, D. L., & Abress, L. (2004). The association of donor center characteristics with attrition from the national marrow donor registry. *Transplantation*, *77*(6), 874-880. | Wrong population |
| Narayanan, P., & Hashmi, S. K. (2016). Knowledge, Experience, and Attitudes of Medical Students Towards the Bone Marrow Registry. *Biology of Blood and Marrow Transplantation*, *22*(3), S371. | Wrong publication type |
| Narayanan, P., Litzow, M., Hogan, W., Patnaik, M., Wolanskyj, A., Ehlers, S., Hashmi, S. (2015). Knowledge and attitudes of medical students towards hematopoietic stem cell transplant and willingness to donate: A survey-based study. *Bone Marrow Transplantation Conference: 41^st^ Annual Meeting of the European Society for Blood and Marrow Transplantation, EBMT 2015*.Turkey. Conference Publication, 50, S364-S365. | Wrong publication type |
| Nicoloso, G., Kürsteiner, O., Bussmann, F., Marbacher, M., & Tiercy, J. M. (2019). A study of selected hematopoietic stem cell donors provided by an intermediate size registry. *European journal of haematology*, *103*(4), 426-432. | Wrong outcome |
| Pahnke, S., Fischer-Nielsen, A., Haastrup, E., Heldal, D., Itala-Remes, M., Kauppila, M., ... & Hagglund, H. (2016). Differences in side effects, sick leave and the will to donate again: the Nordic Register of Haematopoietic Stem Cell Donors. In *Bone Marrow* Transplantation, 51, S328-S329. | Wrong publication type |
| Pahnke, S., Larfors, G., Axdorph‐Nygell, U., Fischer‐Nielsen, A., Haastrup, E., Heldal, D., ... & Hägglund, H. (2018). S hort‐term side effects and attitudes towards second donation: A comparison of related and unrelated haematopoietic stem cell donors. *Journal of clinical apheresis*, *33*(3), 226-235. | Wrong outcome |
| Park, M. A. (1998). A statewide assessment of attitudes, beliefs, and behaviors among blacks toward donation. *Journal of Transplant Coordination*, *8*(1), 25-29. | Wrong population |
| Pawson, R., Griffin, J., Chapman, C., & Rocha, V. (2015, March). Minimising Donor Attrition at Work-Up: the BBMR Experience. In *Bone Marrow* Transplantation, 50, S366-S366. | Wrong publication type |
| Pulsipher, M. A., Logan, B. R., Kiefer, D. M., Chitphakdithai, P., Pedersen, T. L., Stroncek, D. F., ... & Shaw, B. E. (2015). The effect of race, socioeconomic status, and collection center size on bone marrow (BM) and peripheral blood stem cell (PBSC) donor experiences at National Marrow Donor Program (NMDP) collection centers. *Biology of Blood and Marrow Transplantation*, *21*(2), S40-S41. | Wrong publication type |
| Pulsipher, M. A., Logan, B. R., Chitphakdithai, P., Kiefer, D. M., Riches, M. L., Rizzo, J. D., ... & Confer, D. L. (2019). Effect of aging and predonation comorbidities on the related peripheral blood stem cell donor experience: report from the related donor safety study. *Biology of Blood and Marrow Transplantation*, *25*(4), 699-711. | Wrong population |
| Pulsipher, M. A., Logan, B. R., Kiefer, D. M., Chitphakdithai, P., Riches, M. L., Rizzo, J. D., ... & Shaw, B. E. (2019). Related peripheral blood stem cell donors experience more severe symptoms and less complete recovery at one year compared to unrelated donors.  *Haematologica*, *104*(4), 844. | Wrong outcome |
| Richter, E. P., Macher, K., Schultz, U., Schmidt, A. H., Schmidt, H., Ehninger, G., ... & Huetter, G. (2017, October). Quality of life of German stem cell donors-a prospective analysis. In *Transfusion Medicine And* Hemotherapy, 44, 33. | Wrong publication type |
| Ritt, A. (1988). National Bone Marrow Donor Registry to begin recruiting in general population this summer. *JAMA*, *259*(21), 3099-3101. | Wrong publication type |
| Rock, G., Decary, F., McCombie, N., Smiley, R. K., Aye, M. T., & Huebsch, L. (1987). Registry of unrelated bone marrow donors. CMAJ: Canadian Medical Association Journal, 137(4), 294. | Wrong outcome |
| Rosales, M., Ferreira, S., Torres, P., Roncon, S., Barbosa, I., Amado, F., ... & Carvalhais, A. (2010). Satisfaction of unrelated donor after haematopoietic stem cell collection. *Transfusion and Apheresis Science*, (43), S21-S22. | Wrong publication type |
| Rowley, S. D., Donaldson, G., Lilleby, K., Bensinger, W. I., & Appelbaum, F. R. (2001). Experiences of donors enrolled in a randomized study of allogeneic bone marrow or peripheral blood stem cell transplantation. *Blood, The Journal of the American Society of Hematology*, *97*(9), 2541-2548. | Wrong outcome |
| Rueesch, M., el Dusouqui, S. A., Buhrfeind, E., Buser, A., Guengoer, T., Chalandon, Y., ... & Halter, J. (2015, March). Prospective donor outcome follow-up: results and challenges of the first 6 years of Swiss experience. In *Bone Marrow* Transplantation, 50, S362-S363. | Wrong publication type |
| Ruff, P., Schlaphoff, T., Du Toit, E., & Heyns, A. (2008). The South African Bone Marrow Registry (SABMR) and allogeneic bone marrow transplantation: SAMJ forum. *South African Medical Journal*, *98*(7), 516-520. | Wrong publication type |
| Schwartz, S. H. (1970). Elicitation of moral obligation and self-sacrificing behavior: an experimental study of volunteering to be a bone marrow donor. *Journal of personality and social psychology*, *15*(4), 283. | Wrong year |
| Schmidt, A. H. (2014). Potential for increased stem-cell donor recruitment in India. *The Lancet Haematology*, *1*(2), e48-e49. | Wrong publication type |
| Schmidt, A. H., Mengling, T., Hernández-Frederick, C. J., Rall, G., Pingel, J., Schetelig, J., & Ehninger, G. (2017). Retrospective analysis of 37,287 observation years after peripheral blood stem cell donation. *Biology of Blood and Marrow Transplantation*, *23*(6), 1011-1020. | Wrong outcome |
| Schmidt, A. H., Mengling, T., Pingel, J., Rall, G., & Ehninger, G. (2010). Follow-up of 12,559 unrelated donors of peripheral blood stem cells or bone marrow. *Blood*, *116*(21), 365. | Wrong outcome |
| Schutte, L., & Kappel, D. (1997). Barriers to donation in minority, low-income, and rural populations. In *Transplantation Proceedings,* 29 (8), 3746-3747. | Wrong population |
| Seeloff, S., & Botos, L. (2010). Blood Center Based Targeted Recruitment Campaign Increases Minority Blood Donations & Marrow Registrations: SP128. *Transfusion*, *50*. | Wrong publication type |
| Smith, M. E. (2002). *Bone marrow donation: Factors influencing intention in African-Americans*. Duquesne University School of Nursing. | Wrong publication type |
| Snarski, E., Kawka, P., Torosian, T., Achremczyk, M., Skwierawska, K., Waszczuk-Gajda, A., ... & Wiktor-Jedrzejczak, W. (2015, March). Hematopoietic stem cell donors-analysis of motivation and satisfaction throughout the donation process. In *Bone Marrow Transplantation*, 50, S157-S157. | Wrong publication type |
| Stone, S. R. (2019). *Perception of Hematopoietic Stem Cell Donation of Vietnamese-Americans: A Mixed Methods Study* (Doctoral dissertation, Texas Woman’s University). | Wrong publication type |
| Stroncek, D., Shaw, B. E., Logan, B., Kiefer, D. M., Chitphakdithai, P., Switzer, G. E., ... & Pulsipher, M. A. (2016). Pain, Symptoms, Aes, and Recovery after Second Unrelated Donor Collection of Marrow/Peripheral Blood Stem Cells Are Similar to Those of the First Donation. *Blood*, *128*(22), 2177. | Wrong outcome |
| Stroncek, D. F., Shaw, B. E., Logan, B. R., Kiefer, D. M., Savani, B. N., Anderlini, P., ... & Pulsipher, M. A. (2018). Donor experiences of second marrow or peripheral blood stem cell collection mirror the first, but CD34+ yields are less. *Biology of blood and marrow transplantation*, *24*(1), 175-184. | Wrong outcome |
| Stukas, A. A., Dew, M. A., Switzer, G. E., & Simmons, R. G.. (1999). Potential Bone-Marrow Donors and Their Spouses: The Effects of Volunteerism on Distress Levels1. Journal of Applied Social Psychology, 29(1), 1–22. <http://doi.org/10.1111/j.1559-1816.1999.tb01372.x> | Wrong outcome |
| Suluhan, D., Eker, I., Yilmaz, S., Yildiz, D., Kiziler, E., ÇETİNKAYA, R., ... & BESIRBELLIOGLU, B. (2016). Can Voluntary Blood Donors Be a Good Resource for Hematopoietic Stem Cell Donation? Analysis of the Knowledge and Motivation of Voluntary Blood Donors for Hematopoietic Stem Cell Donation: P067. *Bone Marrow Transplantation*, *51*. | Wrong publication type |
| Switzer, G. E., Goycoolea, J. M., Dew, M. A., Graeff, E. C., & Hegland, J. (2001). Donating stimulated peripheral blood stem cells vs bone marrow: do donors experience the procedures differently? *Bone marrow transplantation*, *27*(9), 917-923. | Wrong outcome |
| Switzer, G.E., Simmons, R.G., Dew, M.A., 1996. Helping unrelated strangers: physical and psychological reactions to the bone marrow donation process among anonymous donors. Journal of Applied Social Psychology 26 (6), 469 | Wrong outcome |
| Switzer, G. E., Harrington, D., Haagenson, M. D., Drexler, R., Foley, A., Confer, D. L., ... & Wingard, J. R. (2010). Health-related quality-of-life among adult matched unrelated stem cell donors: a Blood and Marrow Transplant Clinical Trials Network (BMT CTN) randomized trial of marrow versus PBSC donation. *Blood*, *116*(21), 366. | Wrong outcome |
| Switzer, G., Harrington, D., Haagenson, M., Drexler, R., Foley, A., Confer, D., ... & Wingard, J. (2011). Early post-donation health-related quality-of-life and recovery among adult unrelated stem cell donors: a planned subgroup analysis of a randomized trial of marrow versus PBSC donation: O403. *Bone Marrow Transplantation*, *46*. | Wrong publication type |
| Switzer, G. E., Bruce, J. G., Harrington, D., Haagenson, M., Drexler, R., Foley, A., ... & Wingard, J. R. (2014). Health-related quality of life of bone marrow versus peripheral blood stem cell donors: A prespecified subgroup analysis from a phase III RCT—BMTCTN protocol 0201. *Biology of Blood and Marrow Transplantation*, *20*(1), 118-127. | Wrong outcome |
| Switzer, G. E., Bruce, J., Kiefer, D. M., Kobusingye, H., Drexler, R., Besser, R. M., ... & Pulsipher, M. A. (2017). Health-related quality of life among older related hematopoietic stem cell donors (> 60 years) is equivalent to that of younger related donors (18 to 60 years): a related donor safety study. *Biology of Blood and Marrow Transplantation*, *23*(1), 165-171. | Wrong population |
| Szer, J., Elmoazzen, H., Fechter, M., Hwang, W., Korhonen, M., Miller, J., ... & Stein, J. (2016). Safety of living donation of hematopoietic stem cells. *Transplantation*, *100*(6), 1329-1331. | Wrong publication type |
| Usha, S. (2017). KAB Study among Voluntary Blood Donors Regarding their Awareness and Willingness to Join Stem Cell Registry and to Donate Hematopoietic Stem Cells (Doctoral dissertation, The Tamil Nadu Dr. MGR Medical University, Chennai). | Wrong publication type |
| Van Walraven, S. M., Heemskerk, M. B. A., Lie, J. L. W. T., Barge, R. M. Y., Cornelissen, J. J., Egeler, R. M., … Oudshoorn, M.. (2005). The importance of identifying a back-up donor for unrelated stem cell transplantation. Bone Marrow Transplantation, 35(5), 437–440. <http://doi.org/10.1038/sj.bmt.1704812> | Wrong outcome |
| Veldhuizen, I., Nillesen, S., Fechter, M., & van Kraaij, M. (2013). Motivational Differences Between Stem Cell Donors And Regular Blood Donors: P-083. *Vox Sanguinis*, *105*. | Wrong publication type |
| Wanner, M., Bochert, S., Schreyer, I. M., Rall, G., Rutt, C., & Schmidt, A. H. (2009). Losing the genetic twin: donor grief after unsuccessful unrelated stem cell transplantation. *BMC health services research*, *9*(1), 1-10. | Wrong outcome |
| Ware, K. M. (2014). Increasing bone marrow donation (Doctoral dissertation, The College of St. Scholastica). | Wrong publication type |
| Yu, S., Kim, M., Kim, T. G., & Beom, S. H. (2019). Experiences of unrelated hematopoietic stem-cell donors and experts of relevant institutions. Korean Journal of Adult Nursing, 31(5), 522-539. | Wrong language |
| Zaini, R., & Al-Thagafi, A. (2020). Medical Students’ knowledge, attitude towards hematopoietic stem cell transplantation and donation behaviour at Taif university. *Health*, *5*, 1-4. | Wrong population |

Total number of studies excluded: 143

Reasons:

Wrong publication type = 70

Wrong outcome = 38

Wrong population = 26

Wrong Study Design = 3

Wrong language= 5

Wrong year= 1
